# The Potential Role of Epigenetic Mechanisms in the Development of Retinitis Pigmentosa and Related Photoreceptor Dystrophies

**DOI:** 10.3389/fgene.2022.827274

**Published:** 2022-03-11

**Authors:** Galina Dvoriantchikova, Karin Rose Lypka, Dmitry Ivanov

**Affiliations:** ^1^ Department of Ophthalmology, Bascom Palmer Eye Institute, University of Miami Miller School of Medicine, Miami, FL, United States; ^2^ Department of Microbiology and Immunology, University of Miami Miller School of Medicine, Miami, FL, United States

**Keywords:** DNA methylation, histone modifications, DNA demethylation pathway, retina, retinitis pigmentosa, photoreceptor dystrophies

## Abstract

Retinitis pigmentosa and related photoreceptor dystrophies (RPRPD) are rare retinal diseases caused by hereditary gene mutations resulting in photoreceptor death, followed by vision loss. While numerous genes involved in these diseases have been identified, many cases have still not been associated with any gene, indicating that new mechanisms may be involved in the pathogenesis of these photoreceptor dystrophies. Many genes associated with RPRPD regulate photoreceptor specification and maturation in the developing retina. Since retinal development begins with a population of equivalent, proliferating retinal progenitor cells (RPCs) having a specific “competence” in generating all types of retinal neurons, including cone and rod photoreceptors, we tested the epigenetic changes in promoters of genes required for photoreceptor development and genes associated with RPRPD during RPC differentiation into cone and rod photoreceptors. We found that promoters of many of these genes are epigenetically repressed in RPCs but have no epigenetic restrictions in photoreceptors. Our findings also suggest that DNA methylation as an epigenetic mark, and DNA demethylation as a process, are more important than other epigenetic marks or mechanisms in the pathogenesis of these diseases. Most notably, irregularities in the DNA demethylation process during the RPC-to-photoreceptor transition may significantly contribute to retinitis pigmentosa (RP) pathogenesis since genes with hypermethylated promoters in RPCs account for at least 40% of autosomal recessive RP cases and at least 30% of autosomal dominant RP cases. Thus, we proposed an epigenetic model according to which unsuccessful demethylation of regulatory sequences (e.g., promoters, enhancers) of genes required for photoreceptor development, maturation, and function during the RPC-to-photoreceptor transition may reduce or even eliminate their activity, leading to RPRPD without any inheritable mutations in these genes.

## Introduction

Retinitis pigmentosa (RP), cone and cone-rod dystrophy (CCRD), congenital stationary night blindness (CSNB), Leber congenital amaurosis (LCA), and juvenile macular degenerations (MD; e.g., Stargardt disease and Best vitelliform macular dystrophy) are characterized by progressive rod and/or cone photoreceptor loss resulting in poor vision or even blindness (Hartong et al., 2006; Zeitz et al., 2015; Altschwager et al., 2017; Tsang and Sharma, 2018; Gill et al., 2019). There’s no cure for these diseases and they are caused by hereditary mutations in various genes, many of which regulate rod and cone photoreceptor specification, maturation, and function in the developing retina (e.g., *CRX, NRL, NR2E3, PDE6A, PRPH2, USH2A, RHO*, etc.) (Hartong et al., 2006; Swaroop et al., 2010; Zeitz et al., 2015; Altschwager et al., 2017; Tsang and Sharma, 2018; Gill et al., 2019). While numerous genes involved in these diseases have already been identified, many cases have not yet been associated with any gene (Hartong et al., 2006; Zeitz et al., 2015; Altschwager et al., 2017; Tsang and Sharma, 2018; Gill et al., 2019). For example, identified RP genes account for only about 60% of all patients (Hartong et al., 2006). The unsuccessful search for mutated genes in the era of next-generation sequencing and increasing international collaborative research suggest that different mechanisms may be involved in the pathogenesis of RP, CCRD, CSNB, LCA, and MD.

Epigenetic changes, like DNA methylation at the cytosine bases and histone modifications in gene promoters, regulate expression of the corresponding genes (Zhou et al., 2011; Corso-Diaz et al., 2018). These processes are essential to tissue development and any irregularities in these processes can lead to pathology (Zhou et al., 2011; Corso-Diaz et al., 2018). We discovered recently that the promoters of genes such as *Nr2e3, Pde6a, Pde6b, Pde6g, Pde6c, Pde6h, Cnga1, Cngb1*, and *Rho* were highly methylated (hypermethylated) in DNA isolated from retinal progenitor cells (RPCs)—progenitors which differentiate to generate all retinal cell types including photoreceptors (Dvoriantchikova et al., 2019b; Dvoriantchikova et al., 2019c). The methylation of these promoters was significantly reduced during RPC differentiation into photoreceptors and accompanied by an increased expression of the corresponding genes (Dvoriantchikova et al., 2019b; Dvoriantchikova et al., 2019c). Similar results were obtained in two other studies (Merbs et al., 2012; Kim et al., 2016). All genes above are not only required for photoreceptor development and function, but also mutations in these genes lead to RP and related photoreceptor dystrophies (Hartong et al., 2006; Zeitz et al., 2015; Altschwager et al., 2017; Tsang and Sharma, 2018; Gill et al., 2019). It is generally accepted that DNA methylation in promoter regions silences gene expression, while DNA demethylation should occur to allow gene expression (Corso-Diaz et al., 2018). Hence, unsuccessful demethylation of the promoters of these genes during RPC differentiation into photoreceptors may reduce or even eliminate their activity, leading to rod and/or cone photoreceptor dystrophies. Thus, we hypothesize that not only mutations in DNA but also retina-specific epigenetic changes in the DNA may contribute to the pathogenesis of RP and related inherited retinal diseases. Since not only DNA methylation but also other permissive and repressive (temporally or permanent) epigenetic marks may contribute to the pathogenesis of RP, CCRD, CSNB, LCA, and MD, we performed in this study an in-depth analysis of epigenetic states of promoters of all known genes involved in photoreceptor development, function, and pathology during the RPC-to-photoreceptor transition to collect evidence supporting this hypothesis. Our findings suggest that RP and related photoreceptor dystrophies may be not only genetic disorders but also epigenetic disorders. A similar situation transpired 20 years ago in cancer research, when the role of gene modifications (mutations, deletions, etc.) was seen as a key contributor (cancer as a genetic disease) (Balmain, 2001; Feinberg and Tycko, 2004). A vast amount of data obtained in recent years made it possible to confirm that many types of cancers are epigenetic diseases (Feinberg and Tycko, 2004; Herceg and Hainaut, 2007; Sharma et al., 2010; Issa, 2017; Cheng et al., 2019). Thus, if our hypothesis is correct and RP, CCRD, CSNB, LCA, and MD are epigenetic disorders, it may significantly change current approaches to diagnosing and treating these diseases.

### Promoters of Genes, Whose Mutations Lead to Retinitis Pigmentosa, Cone and Cone-Rod Dystrophy, Congenital Stationary Night Blindness, Leber Congenital Amaurosis, and MD, Are Highly Methylated in RPCs and Show DNA Demethylation During Differentiation of RPCs Into Photoreceptors

**FIGURE 1:**
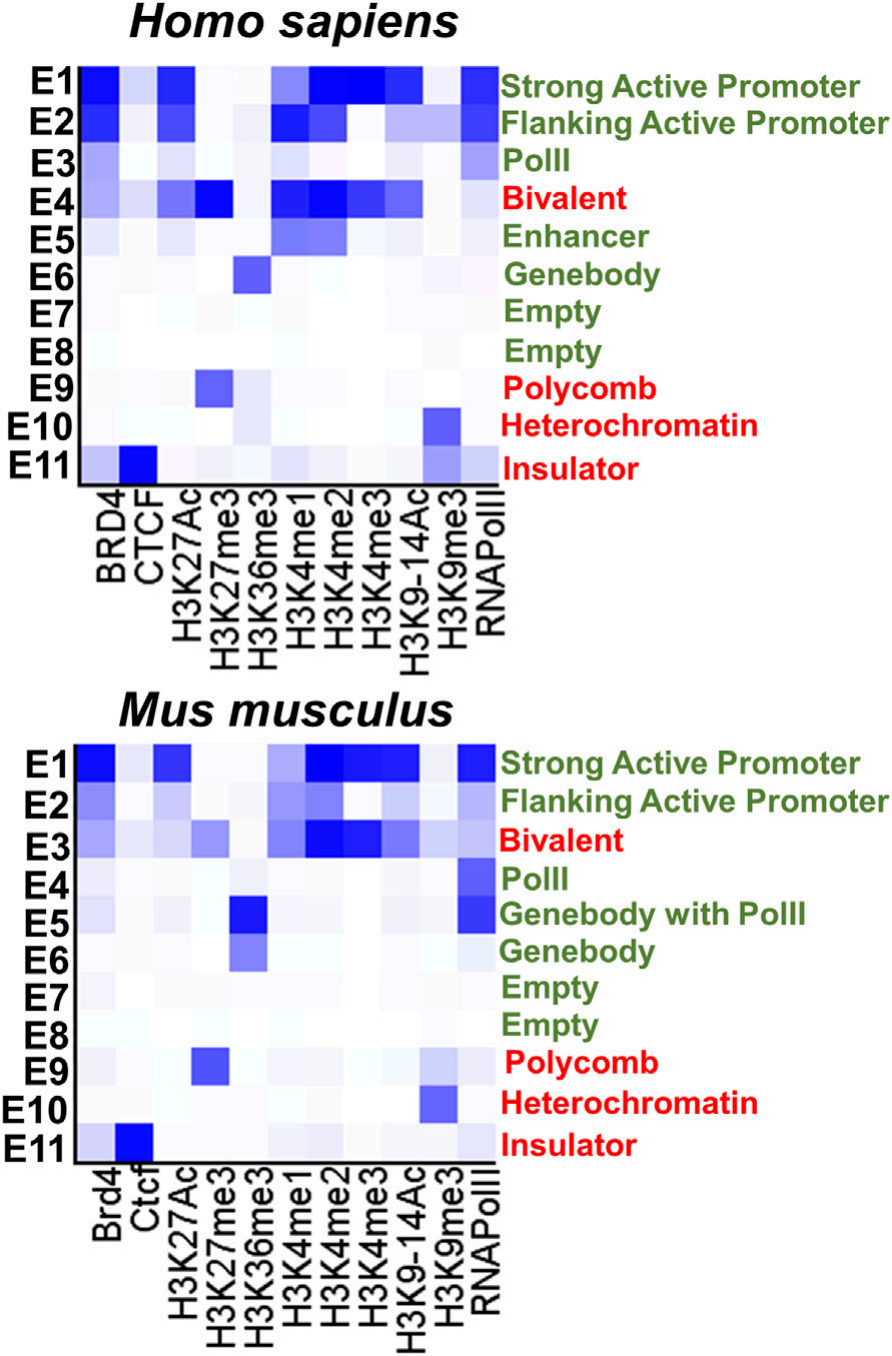
The eleven chromatin states (permissive states are marked in green, repressive states are marked in red) were identified with the chromHMM software package using human and mouse retinal ChIP-seq data. The darker blue color in the heat maps labels abundant ChIP-seq marks in the chromatin state.

To test the epigenetic changes in promoters of genes associated with RP, CCRD, CSNB, LCA, and MD during the RPC-to-photoreceptor transition, we first acquired the lists of corresponding genes using the RetNet database (https://sph.uth.edu/retnet/). We studied promoters of **1**) 83 genes involved in retinitis pigmentosa - RP, **2**) 32 genes involved in cone and cone-rod dystrophy—CCRD, **3**) 13 genes involved in congenital stationary night blindness—CSNB, **4**) 25 genes involved in Leber congenital amaurosis - LCA, and **5**) 17 genes involved in juvenile macular degeneration—MD (Supplementary Data S1 and S2). To characterize the epigenetic states of studied promoters we used **1**) human and mouse genome-wide H3K4me1, H3K4me2, H3K4me3, H3K36me3, H3K27Ac, H3K27me3, H3K9-14Ac, H3K9me3 histone modification, BRD4, CTCF transcription factor, and RNA PolII ChIP-seq retinal data, and **2**) whole-genome bisulfite sequencing (WGBS) data from (**a**) human retinas at fetal week (FW) 10, 14, 21, and 23 (the majority of cells in human fetal retinas at these time points are RPCs), (**b**) mouse developing retinas starting from embryonic (E) day 14.5 (the majority of cells are RPCs) till postnatal (P) day 21 (the majority of cells are rod photoreceptors) (NCBI-GEO GSE87064) (Aldiri et al., 2017). We also used WGBS data from mouse retinas at E11.5 and E12.5 (the majority of cells in murine retinas at these time points are RPCs) and RPCs isolated from P0 and P3 mouse retinas (NCBI-GEO GSE126474) (Dvoriantchikova et al., 2019b). To study the methylome of mouse rod and cone photoreceptors, we used WGBS data (NCBI-GEO GSE84589) (Hartl et al., 2017). Histone modifications alone do not carry essential information, while certain combinations of these modifications characterize the chromatin epigenetic states of studied promoters. Thus, to identify the chromatin states, we used computational, multivariate Hidden Markov Models (chromHMM) with all of ChIP-seq data above following annotation (Ernst and Kellis, 2012; Cavalcante and Sartor, 2017). Using this approach, we identified 11 chromHMM chromatin states of studied human and mouse genes (Figure 1). We considered that “bivalent” and “polycomb” chromatin states are temporally repressive and can be transitioned into a permissive state under the right circumstances. Meanwhile, “insulator” and “heterochromatin” states are permanently repressive. We considered the remaining chromatin states as permissive states. The “empty” chromatin state is one of the permissive chromatin states characterized by the absence of **1**) studied histone modifications (not excluding the presence of unmodified histones), **2**) studied transcription factors, and **3**) RNA PolII. WGBS data were analyzed using the Hidden Markov Model and change-point based methods (the Bioconductor R packages “methylKit” and “MethylSeekR”) to identify differentially methylated regions (methylome states) following annotation (Akalin et al., 2012; Burger et al., 2013; Cavalcante and Sartor, 2017). Using these data, we identified methylome (hypomethylated and hypermethylated) states of each promoter of studied human and mouse genes.

**FIGURE 2:**
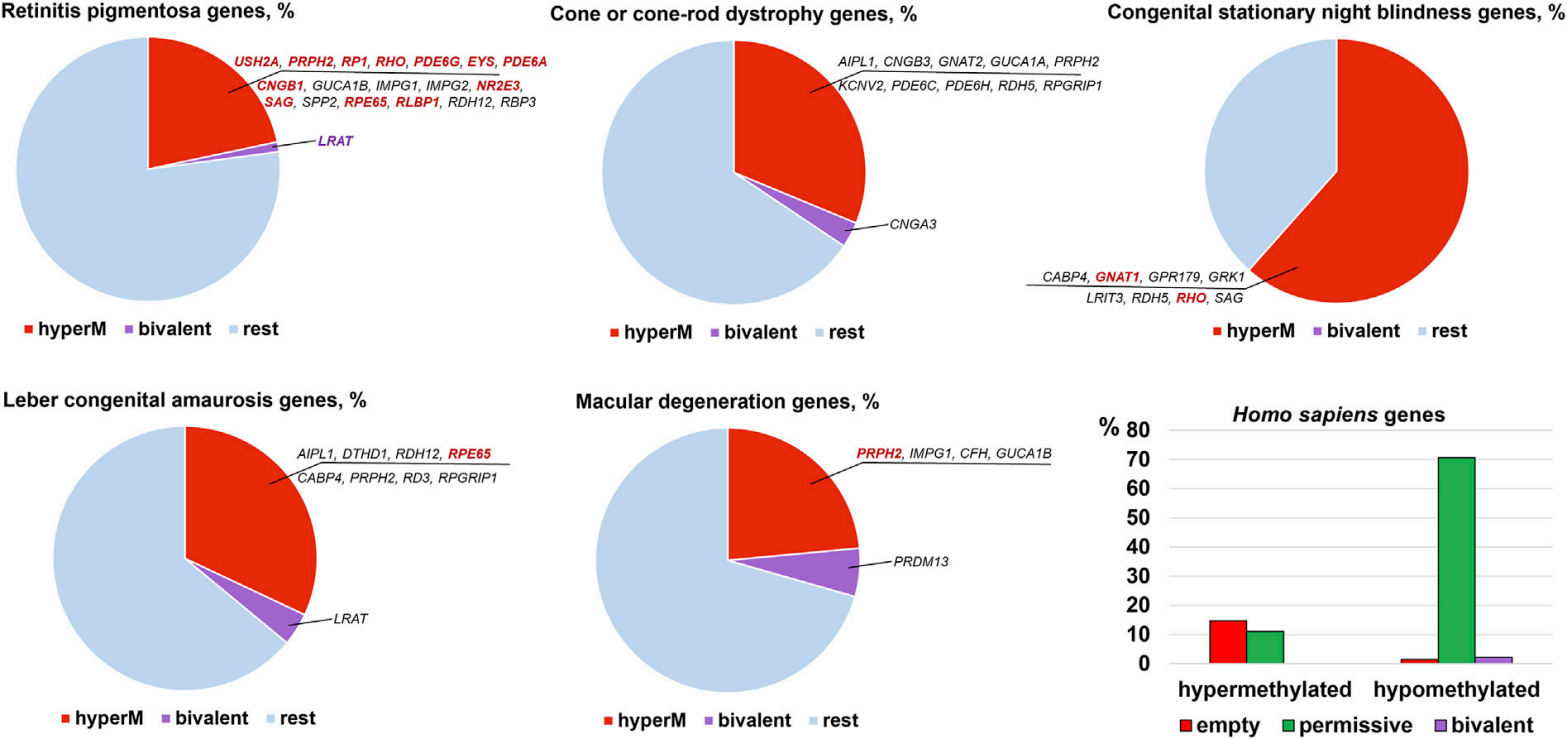
Promoters of many genes associated with retinal inherited diseases are hypermethylated in human embryonic retinas at FW10-FW23, time points at which the majority of cells are RPCs. Meanwhile, the number of genes with bivalent promoters associated with the studied disease was 1 or 0. Many hypermethylated promoters were in an “empty” chromatin state. Mutations in the genes colored bright red account for the majority of cases of the respective diseases.

**FIGURE 3:**
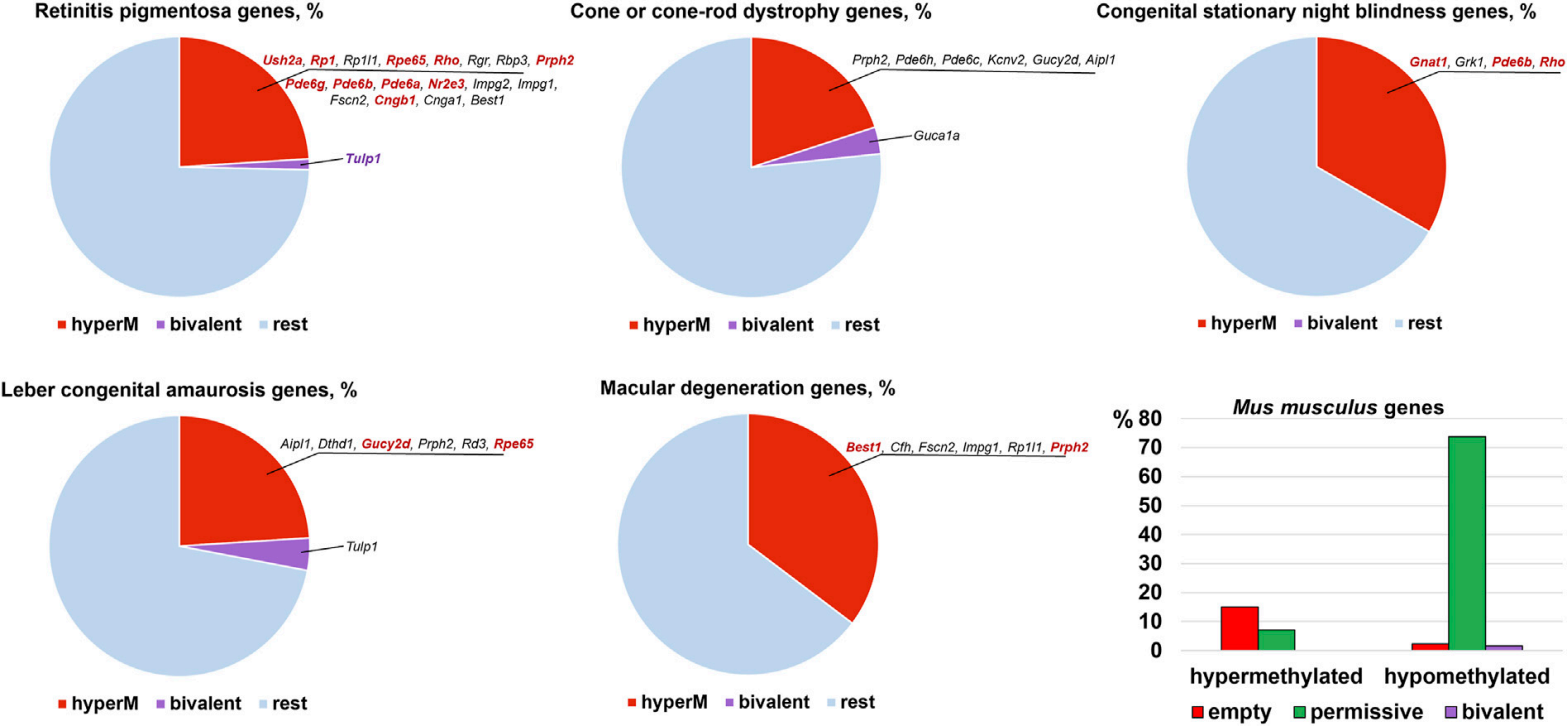
The many mouse homologs of human genes whose mutations lead to RP, CCRD, CSNB, LCA, and MD have the same hypermethylated promoters in embryonic retinas/RPCs as human genes, while genes with bivalent promoters were different. Many promoters in an “empty” chromatin state were hypermethylated. Mutations in the genes colored bright red account for the majority of cases of the respective diseases.

**FIGURE 4:**
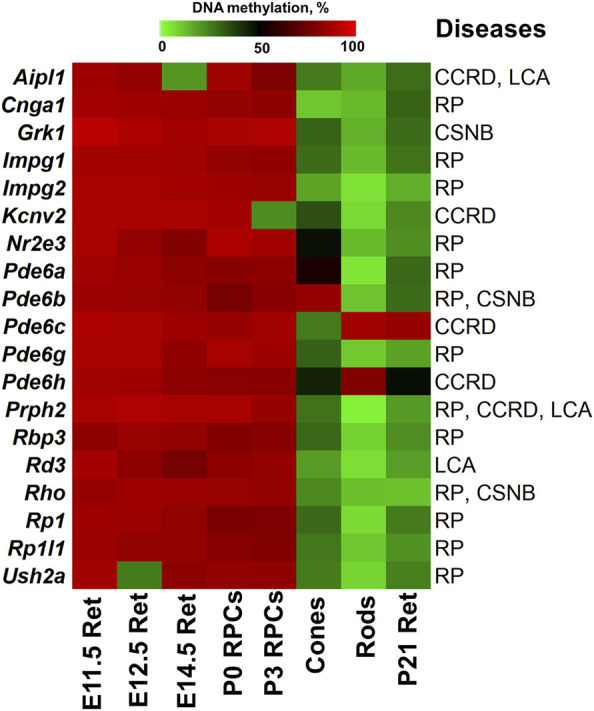
The generated heat map reflects DNA demethylation in the promoters of genes associated with RP, CCRD, CSNB, LCA and MD during the differentiation of RPCs into rod and cone photoreceptors. E11.5 Ret, E12.5 Ret, E14.5 Ret correspond to embryonic day (E) 11.5, 12.5 and 14.5 mouse retinas; P0 RPCs and P3 RPCs correspond to RPCs isolated from P0 and P3 retinas; cones and rods correspond to mature cone and rod photoreceptors; P21 Ret—postnatal (P) day 21 retinas. (retinitis pigmentosa—RP, cone and cone-rod dystrophy - CCRD, congenital stationary night blindness—CSNB, Leber congenital amaurosis—LCA, and macular (Stargardt and vitelliform) degeneration—MD).

The results of our analysis can be found in Supplementary Data S1, S2, S3, S4, and S5 and summarized in Figures 2, 3. Our data indicate that promoters of 18 genes associated with RP were hypermethylated in human (Figure 2) and mouse (Figure 3) RPCs. While promoters of some genes were hypermethylated in one species and hypomethylated in another, the majority of hypermethylated genes were the same in the RPCs of both species. These genes include *CNGB1, IMPG1, IMPG2, NR2E3, PDE6A, PDE6G, PRPH2, RBP3, RHO, RP1, RPE65*, and *USH2A*. It should be noted that mutations in human *CNGB1, NR2E3, PDE6A, PDE6G, PRPH2, RHO, RP1, RPE65*, *USH2A,* and in human *EYS* (no homolog was identified in mouse genome) account for at least 40% of autosomal recessive RP (e.g., *EYS*/10–20%, *USH2A*/17%) and at least 30% of autosomal dominant RP (e.g., *RHO*/25%) cases (Supplementary Data S2) (Hartong et al., 2006). It should be noted that many hypermethylated promoters of these genes were in an “empty” chromatin state (Supplementary Data S2, S4). The majority of genes whose promoters were hypermethylated in mice RPCs had hypomethylated promoters in mice rod and cone photoreceptors, as expected (Figure 4; Supplementary Data S4). Meanwhile, we found that only two genes, *LRAT,* in human RPCs (Figure 2) and *Tulp1* in murine RPCs (Figure 3), were in a “bivalent” (temporally repressive) chromatin state (Supplementary Data S2, S4). The promoters of these genes were hypomethylated. Mutations in human *LRAT* and *TULP1* are associated with 1% of autosomal recessive RP cases (Hartong et al., 2006). *Tulp1* promoter, “bivalent” in mice RPCs, was in a permissive chromatin state in mature photoreceptors (Supplementary Data S4, S5).

The analysis of genes associated with CCRD revealed that promoters of 10 genes in human RPCs (Figure 2) and six genes in murine RPCs (Figure 3) were hypermethylated (Supplementary Data S2, S4). The genes whose promoters were hypermethylated in both species include *AIPL1, KCNV2, PDE6C, PDE6H*, and *PRPH2*. We found only two genes, *CNGA3* in human RPCs (Figure 2) and *Guca1a* in murine RPCs (Figure 3), in a “bivalent” chromatin state. Mutations in these genes may not account for a substantial number of CCRD cases (Supplementary Data S2). Our analysis of CSNB genes indicate that promoters of seven genes in human RPCs (Figure 2) and four genes in murine RPCs (Figure 3) were hypermethylated (Supplementary Data S2, S4). *GNAT1*, *GRK1*, and *RHO* promoters were hypermethylated in both species. No promoters in studied genes were found in repressive chromatin states. It should be noted that mutations in *GNAT1*, and *RHO* cause many cases of autosomal recessive CSNB (Supplementary Data S2). The examination of genes whose mutations lead to LCA showed that promoters of eight genes in human RPCs (Figure 2) and six genes in murine RPCs (Figure 3) were hypermethylated. The genes whose promoters were hypermethylated in human and murine RPCs include *AIPL1, DTHD1, RD3, RPE65*, and *PRPH2* (Figures 2, 3). Similar to RP, we found that only two genes, *LRAT* in human RPCs and *Tulp1* in murine RPCs, were in a “bivalent” chromatin state. Except *RPE65*, mutations in the genes may not account for a substantial number of LCA cases (Supplementary Data S2). Finally, we found that genes associated with MD have hypermethylated promoters in human (4 genes) and murine (6 genes) RPCs. The genes whose promoters were hypermethylated in both species include *CFH, IMPG1,* and *PRPH2*. While we found one “bivalent” gene, *PRDM13*, in human RPCs, no genes with repressive promoters were detected in murine RPCs (Figures 2, 3). It should be noted that mutations in *PRPH2* account for 25% of Best vitelliform macular dystrophy cases (Supplementary Data S2) (Altschwager et al., 2017). The many promoters of genes associated with CCRD, CSNB, LCA, and MD, hypermethylated or “bivalent” in RPCs, were hypomethylated and permissive in mature cone or rod photoreceptors (Figure 4; Supplementary Data S2, S4). We also noted that many hypermethylated promoters were also in an “empty” chromatin state (Figures 2, 3).

### Promoters of Genes Essential for Rod and Cone Photoreceptor Specification and Maturation Are Hypermethylated in RPCs and Show DNA Demethylation During the RPC-To-Photoreceptors Transition

**FIGURE 5:**
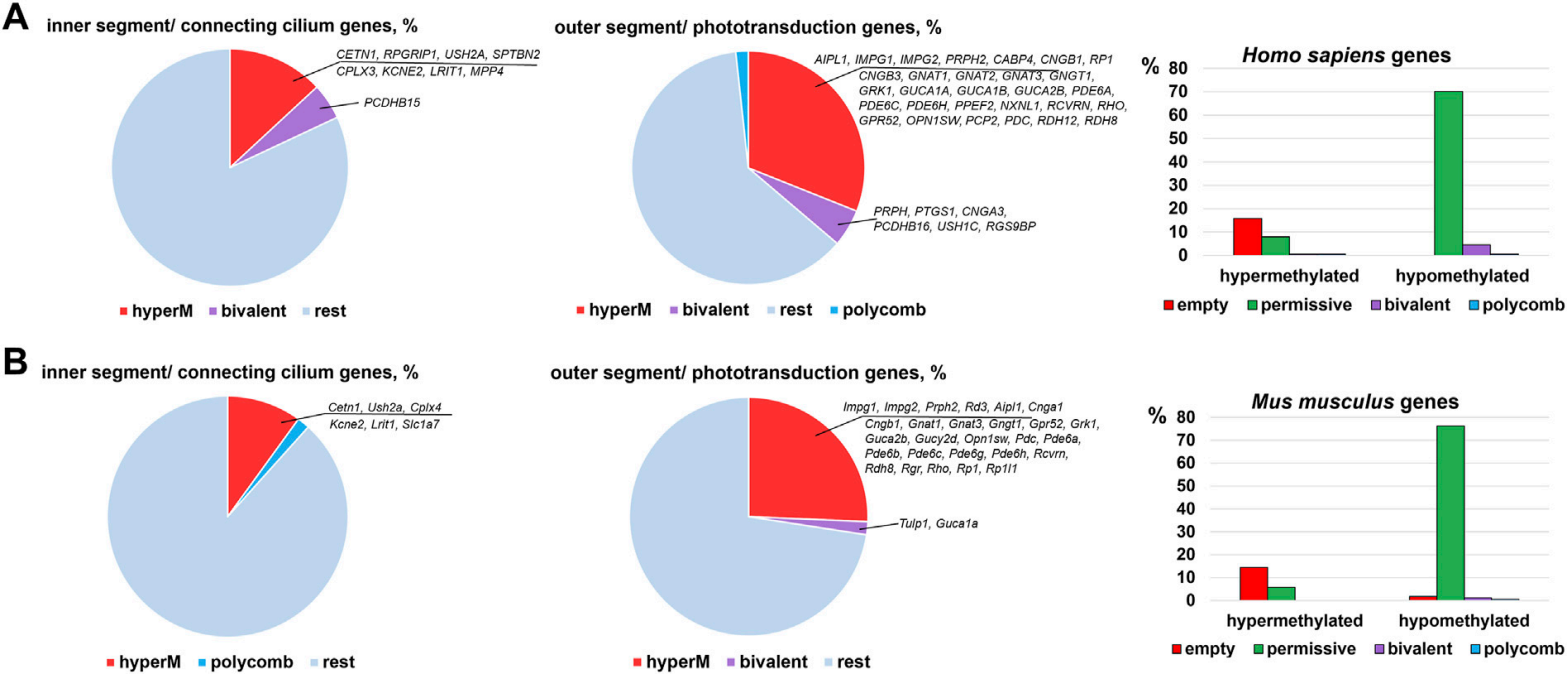
Many promoters of genes required for photoreceptor inner and outer segment function are hypermethylated in human **(A)** and mouse **(B)** embryonic retinas/RPCs.

**FIGURE 6:**
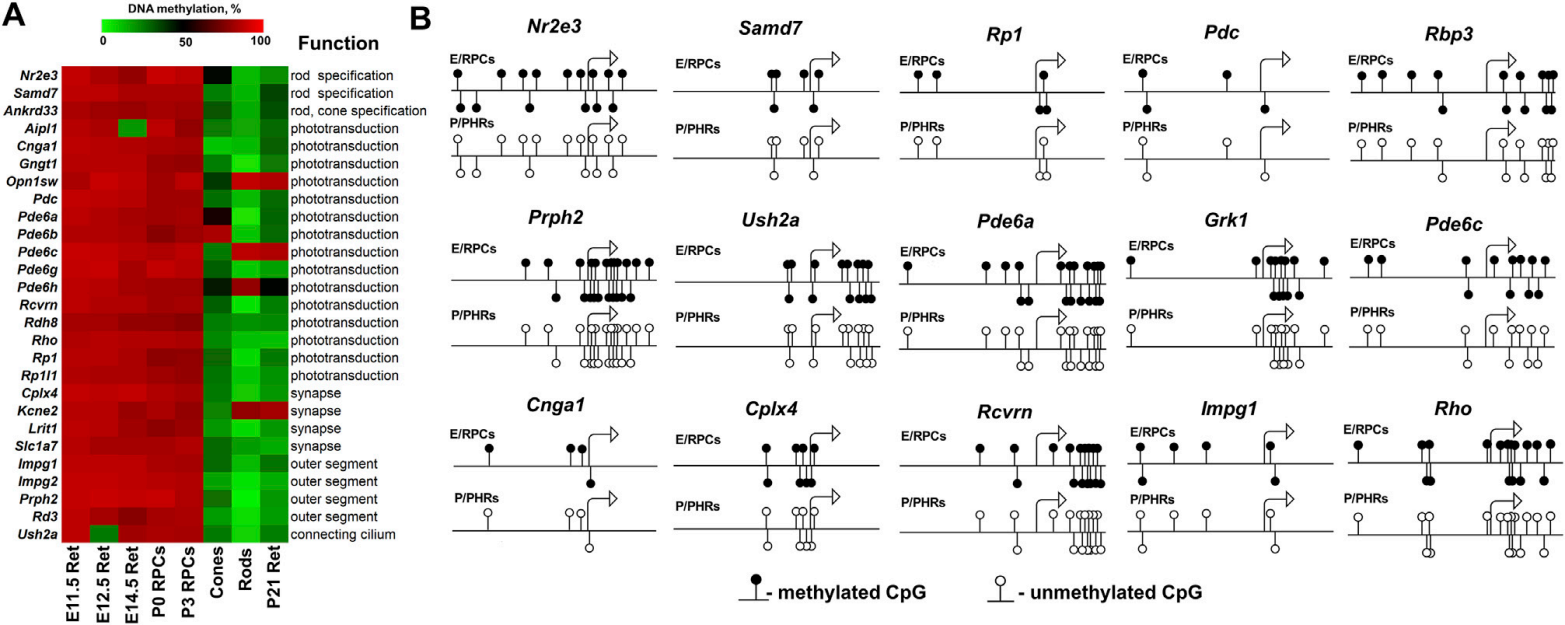
The DNA demethylation process is associated with photoreceptor development. **(A)** Hypermethylated promoters of genes required for photoreceptor specification/maturation/function show DNA demethylation during the differentiation of RPCs into photoreceptors. **(B)** The analysis of the methylation dynamics of individual cytosine bases in the promoter (1,000 bp) and first exon (500 bp) regions of photoreceptor genes revealed that cytosines close to a transcription start site (TSS) were mostly affected. (E/RPCs—embryonic retina/RPCs; P/PHRs—postnatal mature retina/photoreceptors).

Many genes associated with the RP, CCRD, CSNB, LCA, and MD are critical for photoreceptor specification (*CRX, NRL, NR2E3*) and maturation/function (*PDE6A, PDE6G, PDE6C, PDE6H, GNAT1, RHO, USH2A, PRPH2*, etc.) (Swaroop et al., 2010). Hence, it is not surprising that these diseases typically affect younger people, since pathological photoreceptor development may lead to rod and/or cone death and retinal degeneration at an early age. Our findings above suggest that the activity of many of these genes are epigenetically repressed in RPCs but do not have epigenetic restrictions in mature photoreceptors. Since many RP, CCRD, CSNB, LCA, and MD cases have not been associated with any gene yet and epigenetic repression of genes critical for photoreceptor specification and maturation/function may lead to these diseases, we analyzed DNA methylome and chromatin states in the promoters of genes involved in these processes using the results of our analysis above. The lists of photoreceptor inner segment/connecting cilium genes and outer segment/phototransduction genes were acquired from the Gene Ontology (GO) knowledgebase (http://geneontology.org/). We found a small number of genes whose promoters were in “bivalent” or “polycomb” chromatin states; these “bivalent” and “polycomb” promoters were species specific (Figure 5, Supplementary Data S6, S7). Meanwhile, we found that DNA methylation is a stable epigenetic mark in both species. We also found that the number of outer segment/phototransduction genes, whose promoters were hypermethylated in human and murine RPCs, was twice the number of inner segment/connecting cilium genes, whose promoters were hypermethylated in these progenitors (Figure 5, Supplementary Data S6, S7). Meanwhile, the promoters of all of these genes were hypomethylated in mature photoreceptors (Figure 6A, Supplementary Data S6, S7). Our data indicate that the majority of the genes whose promoters were demethylated during the RPC-to-photoreceptor transition are involved in the phototransduction process (Figure 6A). It should be noted that three genes (*Nr2e3, Samd7* and *Anked33*) involved in photoreceptor specification during retinal development were hypermethylated in RPCs and hypomethylated in mature photoreceptors (Figure 6A). Similar to above, we observed that many hypermethylated promoters also had an “empty” chromatin state (Figure 5, Supplementary Data S6, S7). Since the methylKit and MethylSeekR R Bioconductor packages provide only the mean percent of methylation per genomic region, we collected data regarding the % methylation of individual cytosine bases in the promoter region and first exon of the studied murine genes to evaluate the methylation dynamics for these individual cytosine bases during RPC differentiation into photoreceptors (Supplementary Data S8). Our data indicate that the methylated individual cytosines in RPCs were mostly located close to the transcription start site (TSS) in many of the studied genes (Figure 6B; Supplementary Data S8). These cytosine bases were unmethylated in mature photoreceptors, suggesting that the methylation of these cytosines may affect the initiation of transcription of the studied genes.

The results of our analysis suggest that DNA methylation as an epigenetic mark plays a significant role in photoreceptor development and function in both studied species, while the role of “bivalent” promoters is less important and is species specific. To evaluate significance of this observation, we tried to identify all genes whose promoters were in a “bivalent” chromatin state or were hypermethylated in RPCs but were permissive/hypomethylated in mature photoreceptors. To identify “bivalent” genes, we collected only genes whose promoters were bivalent in E14.5, E17.5, P0 and P3 retinas, while the same promoters were in a permissive chromatin state in retinas of P10, P14, P21 and adult mice (Supplementary Data S9). We uploaded ChIP-seq data in Integrated Genome Browser to visually verify the chromatin state in promoters of identified genes (Supplementary Data S10). To identify “hypermethylated” genes, we collected only genes whose promoters were hypermethylated in retinas of E11.5, E12.5, and E14.5 mice as well as in RPCs isolated from P0 and P3 retinas, while the same promoters were hypomethylated in P21 retinas as well as in cones and rods isolated from retinas of adult animals (Supplementary Data S9). Using these strict criteria, we found only four genes with “bivalent” promoters and 35 genes whose promoters were hypermethylated in murine embryonic retinas/RPCs; these promoters were in a permissive state/hypomethylated in mature retinas/photoreceptors (Supplementary Data S9). While the function of identified genes with “bivalent” promoters has not been fully established, 26 out of 35 hypermethylated genes are involved in photoreceptor specification, maturation, and function (Supplementary Data S9). A substantial number of these genes are involved in the phototransduction process. Thus, trying to find any genes satisfying our conditions, we found that DNA methylation as an epigenetic mark and DNA demethylation as a process are mostly associated with RPC differentiation into photoreceptors.

### The Ten-Eleven Translocation Protein and Methyltransferase Families Are Expressed in RPCs, Developing, and Mature Photoreceptors

**FIGURE 7:**
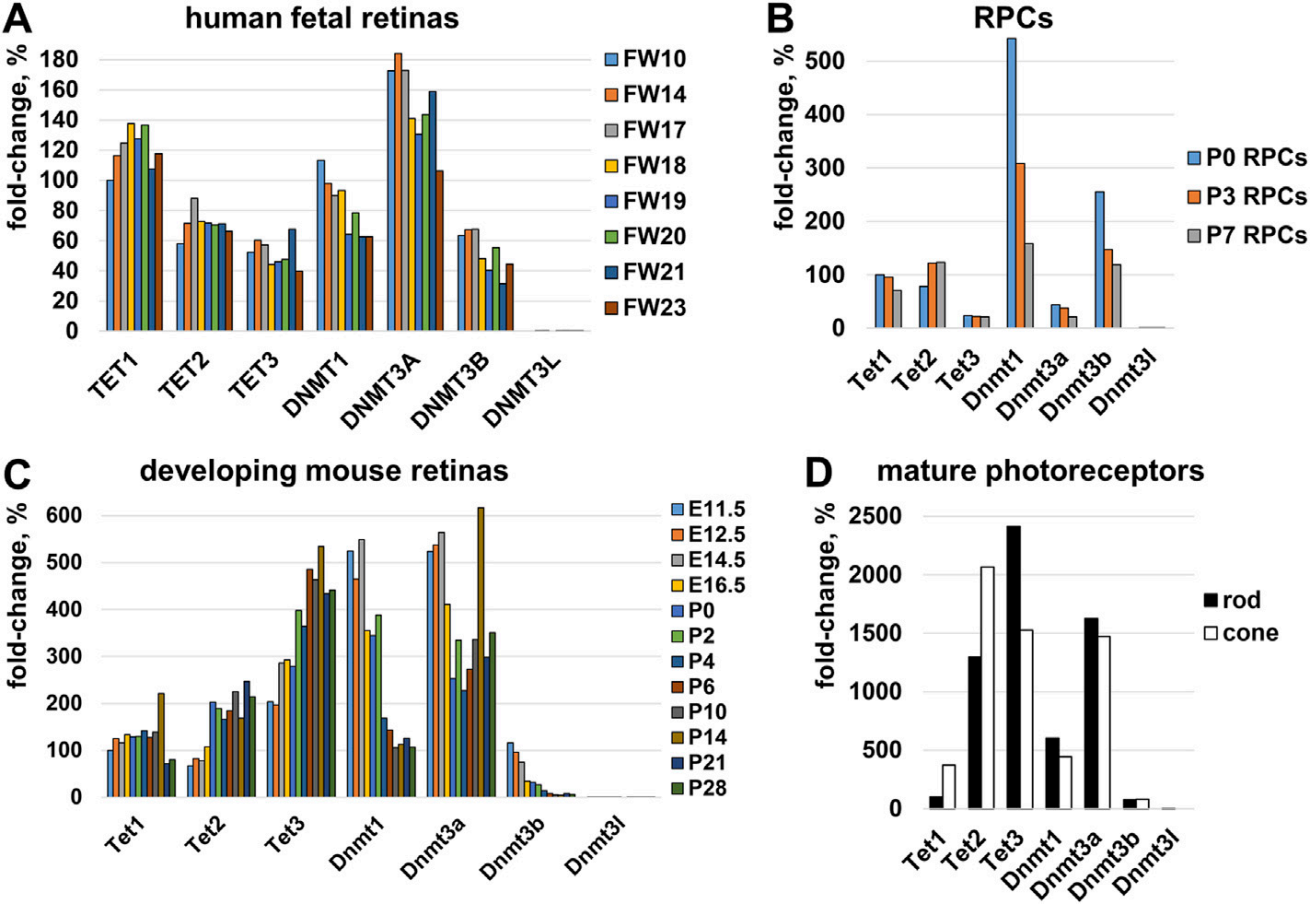
The expression of genes belonging to the TET and DNMT families was evaluated in **(A)** human embryonic retinas (FW-fetal week), **(B)** murine RPCs, **(C)** developing mouse retinas, and **(D)** mature rod and cone photoreceptors. For each gene, the results are expressed as a percentage of the corresponding values of Tet1 at the earliest time point in the studied tissue/cells.

DNA methylation—the epigenetic mechanism used by cells to modulate gene expression—is regulated by two juxtaposed biological processes: the DNA methylation and DNA demethylation pathways (Corso-Diaz et al., 2018). Patterns of methylated cytosines are established by the methyltransferase (DNMT) family, while the Ten-Eleven Translocation (TET) protein family promotes DNA demethylation (Corso-Diaz et al., 2018). Genome-wide DNA methylation/demethylation pathway analysis revealed that DNMTs have global activity, methylating DNA wherever possible until something interferes with them, while TETs have a local effect, demethylating regulatory sequences (e.g., promoters, enhancers) and then stay there to safeguard from *de novo* methylation by DNMT enzymes (Rasmussen et al., 2015; Wiehle et al., 2016; Verma et al., 2018; Lopez-Moyado et al., 2019; Charlton et al., 2020). Since both families establish DNA methylation patterns in cells, we evaluated the expression of the corresponding genes in: 1) human embryonic retinas using GSE87042 RNA-seq data; 2) developing mouse retinas using GSE101986 RNA-seq data; 3) mature rod and cone photoreceptors using GSE72550 RNA-seq data; and 4) RPCs using our published data (Dvoriantchikova et al., 2019a). We found that *Tet1*, *Tet2*, *Tet3*, *Dnmt1*, *Dnmt3a*, and *Dnmt3b* genes had high expression during photoreceptor development (Figure 7).

### Epigenetic Model and Facts That Support It

Emerging evidence suggests the critical role of the TET-dependent DNA demethylation pathway during eye development and retinal neurogenesis. Tet3 depletion in developing Xenopus led to malformation of the eye (eyeless) and neural abnormalities (Xu et al., 2012). However, due to functional redundancy from the TET family members (Tet1, Tet2, Tet3), single TET gene knockouts have lacked the pathological phenotype in the eye and retina in other species (Dawlaty et al., 2011; Li et al., 2016; Seritrakul and Gross, 2017). Meanwhile, double or triple TET knockouts demonstrated severe eye and retinal pathologies. Triple Tet1/Tet2/Tet3 murine embryo knockouts demonstrated a complete absence of the anterior neural plate from which eyes start development (Li et al., 2016). Combined mutations of Tet2/Tet3 in zebrafish led to smaller eyes and abnormal brain morphology (Li C. et al., 2015). In a separate study, Seritrakul and Gross found that all neurons in Tet2/Tet3 knockout zebrafish retinas failed to differentiate (Seritrakul and Gross, 2017). However, genetic ablation of TET enzymes had the most impact on RGCs and photoreceptors. The authors found that: 1) the majority of RGCs were undeveloped and did not form axons; 2) most photoreceptors were undeveloped and that the few photoreceptors that differentiate in the Tet2/Tet3 knockouts fail to form outer segments. Seritrakul and Gross found that the expression of many genes required for photoreceptor development and function was reduced in TET-deficient zebrafish retinas. Many human and mouse homologs of these genes have hypermethylated promoters in embryonic retinas/RPCs (Supplementary Data S11). Thus, if promoters of these genes are hypermethylated in zebrafish’s RPCs and stay hypermethylated in differentiating photoreceptors which lack TET activity, it may explain the reduced expression of these genes in TET-deficient zebrafish retinas observed in Seritrakul and Gross’s study, leading to photoreceptor abnormalities and then, degeneration. Thus, TET family activity is an integral part of photoreceptor development and function, while TET-deficiency leads to photoreceptor abnormalities and degeneration.

**FIGURE 8:**
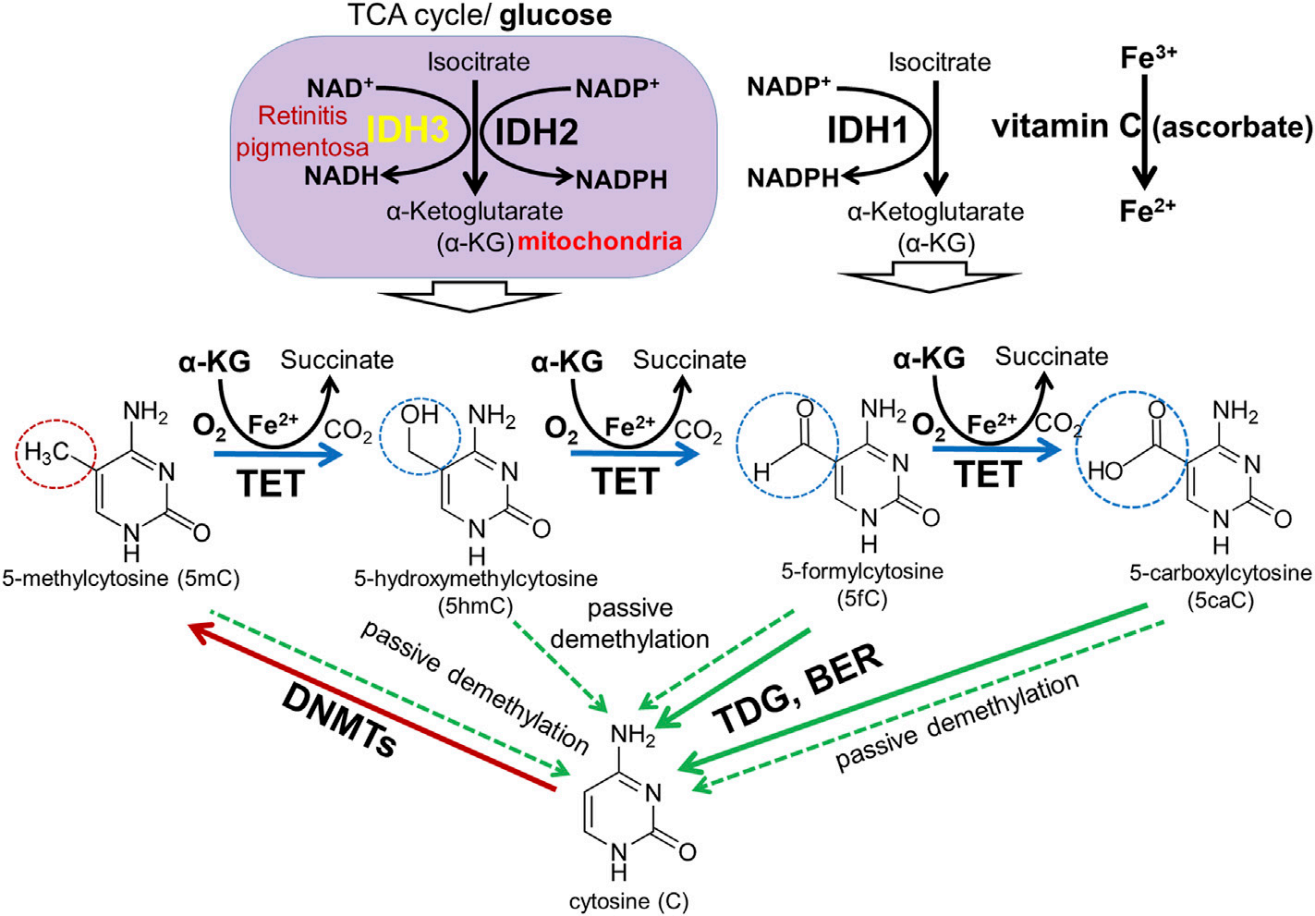
DNA methylation and demethylation pathways: During development, patterns of methylated cytosines are established by the *de novo* methyltransferases Dnmt3a/Dnmt3b, and subsequently preserved through cell divisions by Dnmt1. The TET family promotes DNA demethylation by oxidizing 5mC to produce 5hmC, 5fC, and 5caC in DNA. Oxidized derivatives of 5mC inhibit Dnmt1, promoting passive DNA demethylation (dashed lines). 5fC and 5caC are directly excised by thymine DNA glycosylase (TDG) to generate abasic sites triggering base excision repair (BER) pathway activation followed by replacement of the abasic sites with unmodified cytosines.

TET family dioxygenases depend on DNA (cytosine), oxygen (O2), and α-ketoglutarate (αKG, also known as 2-oxoglutarate, or 2OG) as required substrates and on iron and vitamin C (ascorbate) as cofactors (Vissers et al., 2014; Li et al., 2015b; Yin and Xu, 2016; Yue and Rao, 2020) (Figure 8). αKG is synthesized from glucose in the multistep TCA (Krebs) cycle by the isocitrate dehydrogenase (IDH1-3) family (Hartong et al., 2008; Tommasini-Ghelfi et al., 2019). Mutations in the *IDH3A* and *IDH3B* genes encoding α and β subunits of IDH3 lead to autosomal recessive RP (Hartong et al., 2008; Fattal-Valevski et al., 2017; Pierrache et al., 2017; Tommasini-Ghelfi et al., 2019). Mouse *Idh3a* mutations lead to retinal degeneration (Findlay et al., 2018). It was also proposed that IDH3 enzymes are more important in photoreceptor TCA (Krebs) cycle compared to IDH1 and IDH2 (Hartong et al., 2008). These data suggest that DNA demethylation during photoreceptor development and maturation is regulated by the TET/IDH3-dependent DNA demethylation pathway. Besides mutations in the DNA demethylation pathway, the environment may also play a significant role. Since oxygen and/or glucose levels can affect the activity of TET enzymes, low intracellular oxygen/glucose levels during hypoxic/ischemic stresses may reduce TET enzyme activity, affecting DNA methylation patterns and, in turn, vital for photoreceptor development, maturation, and function gene expression. This statement is consistent with the data according to which TET deficiency exacerbates ischemic brain injury, while increased TET activity protects the brain after stroke (Miao et al., 2015; Morris-Blanco et al., 2019). TET activation by vitamin C leads to significant protection and improved motor function recovery after stroke in young and aged mice of both sexes (Morris-Blanco et al., 2019). Thus, we propose an innovative epigenetic model according to which hereditary gene mutations in the DNA demethylation pathway or hypoxic/ischemic stresses during pregnancy lead to reduced TET enzyme activity in the developing retina and, as a result, unsuccessful demethylation of regulatory sequences (e.g., promoters, enhancers) of the genes required for photoreceptor development, maturation, and function, resulting in photoreceptor dystrophies. If our epigenetic model is correct, the genes involved in the TET/IDH3-dependent DNA demethylation pathway become targets for RP, CCRD, CSNB, LCA, and MD research considering that many RP, CCRD, CSNB, LCA, and MD cases are still not associated with any gene. Our results also offer new insights into the importance of vitamin C intake during pregnancy.

While our results suggest that not only mutations in DNA but also retina-specific epigenetic changes in the DNA (DNA methylation) contribute to the pathogenesis of RP and related photoreceptor dystrophies, the role of epigenetics was still not studied in patients suffering from these diseases. However, such study is complicated by a number of difficulties. Since epigenetic modifications are always tissue-specific, the study (analysis of gene expression and DNA methylation) should be done on retinas (the adult retina is made up of approximately 70% photoreceptors) collected from patients suffering from RP, CCRD, CSNB, LCA, or MD. Since all of these diseases, including RP, are rare diseases and affect mostly young people, patients who already passed away would likely be elderly and blind, and there would be nothing to analyze by the time the retinas could be collected. Meanwhile, obtaining retinal biopsy samples in mild to moderate stages of RP and related photoreceptor dystrophies from living patients would be difficult from an institutional review board (IRB) perspective given the invasive nature of the procedure. In addition, epigenetic mechanisms may contribute to some cases (e.g., 40% or less cases of RP, since 60% of cases are already associated with gene mutations), but not all of them. Hence, investigators need to analyze many samples. Since obtaining human retinal biopsy samples is impossible in the near future, *in vitro* and *in vivo* animal models should be used to study photoreceptor dystrophies as epigenetic disorders.

## Discussion

**FIGURE 9:**
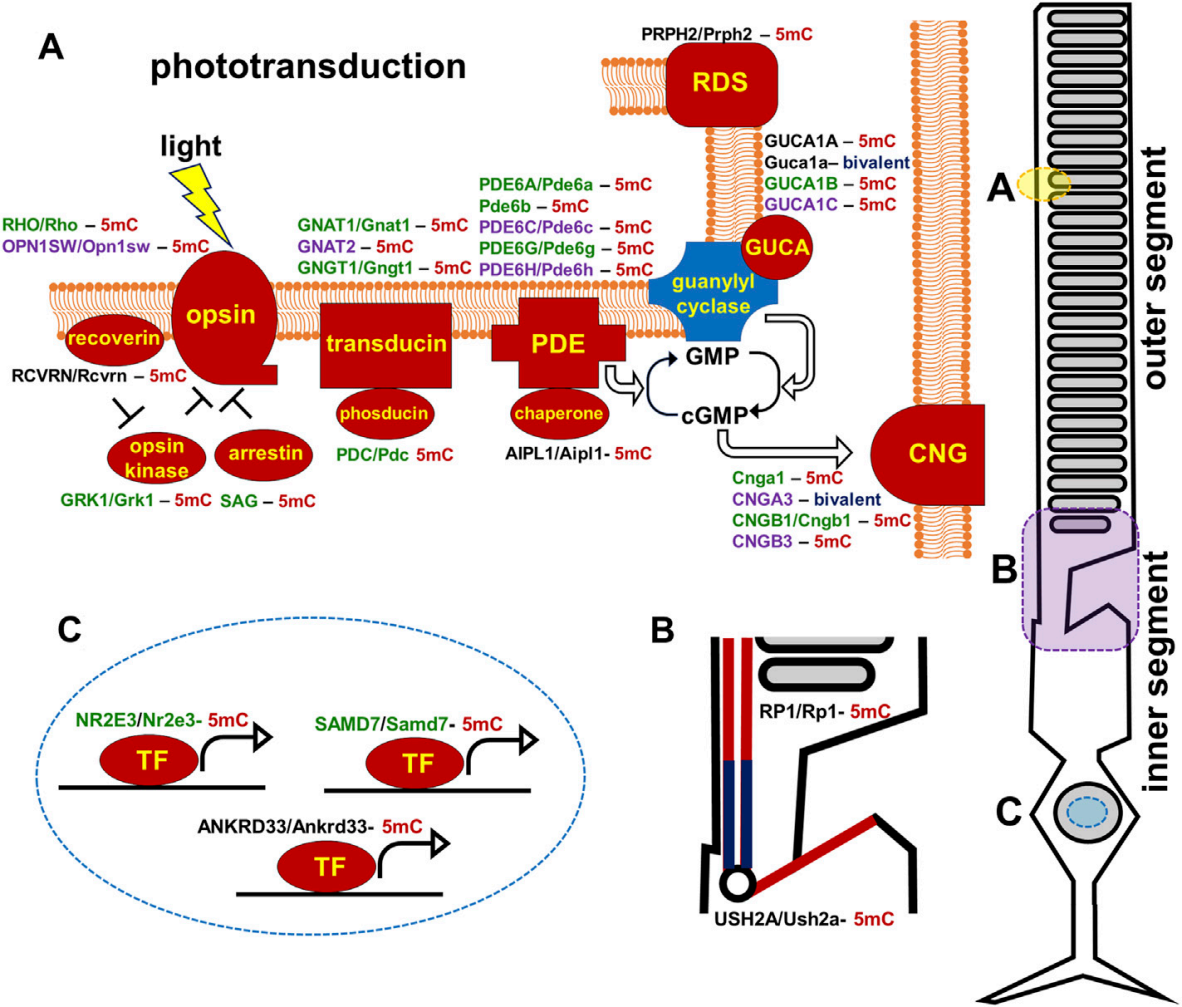
Promoters of human and mouse genes coding molecular components of the photoreceptor outer segment (phototransduction cascade) and inner segment are mostly hypermethylated in embryonic retinas/RPCs (the corresponding proteins are marked in red). The genes are colored green for rods, purple for cones, and black indicates expression in both rods and cones. (5mC- 5-methylcytosine, gene with hypermethylated promoter; bivalent-bivalent promoter).

A widely accepted concept in human genetics is that inherited diseases are caused by mutations, which reduce or eliminate expression of genes important for tissue activity (Badano and Katsanis, 2002). However, our data for retinal inherited diseases suggest the important role of epigenetic mechanisms in the pathogenesis of these diseases. We found that the promoters of many genes mutated in RP, CCRD, CSNB, LCA, and MD (e.g., *USH2A, RHO, PRPH2, EYS, AIPL1, CNGB1, IMPG1, IMPG2, NR2E3, PDE6A, PDE6G, PDE6C, PDE6H, RBP3, RP1*, etc.) were hypermethylated in human and mouse RPCs and were hypomethylated in rod and cone photoreceptors. Dr. Swaroop’s lab demonstrated that promoters of some of these genes were still hypermethylated in DNA isolated from rod precursors, while they were hypomethylated in mature rod photoreceptors (Kim et al., 2016). The levels of DNA methylation in the promoters of these genes showed an inverse correlation with their expression levels (Kim et al., 2016). Similar results were obtained by Dr. Zack’s lab (Merbs et al., 2012). Thus, these data suggest that both RPC and photoreceptor precursor locus-specific DNA demethylation are crucial to promote RPC differentiation into mature photoreceptors. Since DNA methylation silences gene expression, while DNA demethylation should occur to allow gene expression, we proposed an innovative epigenetic model according to which unsuccessful demethylation of regulatory sequences (e.g., promoters, enhancers) of the genes required for photoreceptor development, maturation, and function during the RPC-to-photoreceptor transition may reduce or even eliminate their activity, leading to photoreceptor dystrophies without any inheritable mutations in these genes (Figure 9).

The majority of genes mutated in RP, CCRD, CSNB, LCA, and MD regulate photoreceptor specification, maturation/function (Hartong et al., 2006; Swaroop et al., 2010; Zeitz et al., 2015; Altschwager et al., 2017; Tsang and Sharma, 2018; Gill et al., 2019). We found that, in addition to pathologic gene mutations, abnormalities in mechanisms regulating the DNA demethylation process during rod and cone photoreceptor development may affect photoreceptor function leading to RP, CCRD, CSNB, LCA, and MD (Figure 9). We also observed repressive chromatin marks (mostly bivalent chromatin) in promoters of some genes at the embryonic stage of retinal development, which were replaced with permissive chromatin marks in the mature retina. However, the number of such gene promoters was low compared to hypermethylated gene promoters and, while present in human retinas, they were absent in mice retinas (and *vice versa*). We also found methylated cytosines located close to the transcription start site (TSS) in many hypermethylated gene promoters (Figure 6). The role of methylated cytosine bases in close proximity to a TSS in silencing gene expression was shown previously (Corso-Diaz et al., 2018). Thus, our data suggest that the DNA demethylation process plays a more important role in photoreceptor development and function compared to chromatin modifications considering that DNA demethylation of promoters silenced by hypermethylation is essential to allow gene expression. Since we found that genes with hypermethylated promoters in embryonic retinas/RPCs account for at least 40% of autosomal recessive RP and at least 30% of autosomal dominant RP cases, understanding the significance of the DNA demethylation process in photoreceptor development and function is especially important for this inherited disease because irregularities in its activity may prevent the expression of these genes, leading to photoreceptor death and retinal degeneration in a substantial number of RP patients (Figure 9). DNA demethylation can be carried out passively, when a newly synthesized strand in proliferating cells is not methylated by Dnmt1 after DNA replication, or actively via the DNA demethylation pathway, which requires functioning members of the TET family (Dominguez and Shaknovich, 2014; Li et al., 2015b; Rasmussen and Helin, 2016; An et al., 2017; Bochtler et al., 2017) (Figure 8). However, RPCs are the only dividing cells in the developing retina. Our data also indicate that Dnmt1 has high expression in RPCs (Figure 7). Thus, a passive DNA demethylation process should significantly reduce methylation of regulatory sequences (e.g., promoters, enhancers) of the genes essential for photoreceptor development, maturation, and function in RPCs. This inference is contrary to our observations and, hence, an active DNA demethylation process is required to promote DNA demethylation during the RPC-to-photoreceptor transition. Emerging evidence suggests the critical role of the TET-dependent DNA demethylation pathway in neurogenesis and neurodegenerative diseases (Guo et al., 2011; Xu et al., 2012; Kaas et al., 2013; Rudenko et al., 2013; Zhang et al., 2013; Yao and Jin, 2014; Li et al., 2015b; Li et al., 2016; Wang et al., 2016; Li et al., 2017; Seritrakul and Gross, 2017; Beck et al., 2020; Cochran et al., 2020). TET enzymes require additional cofactors and substrates for their activity including alpha-ketoglutarate (αKG, also known as 2-oxoglutarate, or 2OG), which is generated in the TCA (Krebs) cycle by the isocitrate dehydrogenase (IDH1-3) family (Tommasini-Ghelfi et al., 2019) (Figure 8). It was shown that human *IDH3A* and *IDH3B* mutations are associated with severe early childhood-onset retinitis pigmentosa, and mouse *Idh3a* mutations lead to retinal degeneration (Hartong et al., 2008; Fattal-Valevski et al., 2017; Pierrache et al., 2017; Findlay et al., 2018; Tommasini-Ghelfi et al., 2019). Thus, these data suggest that DNA demethylation during photoreceptor development and maturation is most likely regulated by the TET and IDH3 enzymes.

In conclusion, our study revealed that not only mutations in DNA but also retina-specific epigenetic changes in the DNA may contribute to the pathogenesis of RP, CCRD, CSNB, LCA, and MD. Our findings suggest that the DNA demethylation pathway is more important than other epigenetic mechanisms for photoreceptor specification, maturation, and function since unsuccessful demethylation of regulatory sequences (e.g., promoters, enhancers) during RPC differentiation into photoreceptors should reduce or even eliminate the activity of corresponding genes, leading to RP, CCRD, CSNB, LCA, or MD without any inheritable mutations in these genes (Figure 9). Especially irregularities in the DNA demethylation pathway may contribute to RP pathogenesis (Figure 9). Thus, the results of our study suggest that RP and related photoreceptor dystrophies may not only be genetic disorders but also epigenetic disorders. In recent years, a gene therapy has been viewed as the most promising for treatment of various inherited diseases. However, gene therapy works if a small number of genes (one or two) is affected in one patient. If RP, CCRD, CSNB, LCA, and MD are also epigenetic diseases, activity of many genes (18 hypermethylated genes in the case of RP) may be affected and direct upregulation of all of these genes in one patient using gene therapy is impossible. Meanwhile, upregulation of a small number of genes regulating the DNA demethylation pathway using gene therapy may indirectly promote the expression of target genes and prevent vision loss in patients suffering from RP, CCRD, CSNB, LCA, or MD as epigenetic disorders. Thus, our study may open new avenues to treat these diseases.

## Data Availability

The original contributions presented in the study are included in the article/Supplementary Material, further inquiries can be directed to the corresponding author.
