# Supplementary material for: The Potential Role of Epigenetic Mechanisms in the Development of Retinitis Pigmentosa and Related Photoreceptor Dystrophies
Source: Front Genet. 2022 Mar 11;13:827274. doi: 10.3389/fgene.2022.827274 (PMC8961674; doi:10.3389/fgene.2022.827274)
Supplement: Supplementary file 1 [file DataSheet2.PDF]

Title: The potential role of epigenetic mechanisms in the development of retinitis pigmentosa and related photoreceptor dystrophies

Galina Dvoriantchikova, Bascom Palmer Eye Institute, Department of Ophthalmology, University of Miami Miller School of Medicine, Miami, FL, 33136, USA

Karin Rose Lypka, Bascom Palmer Eye Institute, Department of Ophthalmology, University of Miami Miller School of Medicine, Miami, FL, 33136, USA

Dmitry Ivanov , Bascom Palmer Eye Institute, Department of Ophthalmology, University of Miami Miller School of Medicine, Miami, FL, 33136, USA; Department of Microbiology and Immunology, University of Miami Miller School of Medicine, Miami, FL, 33136, USA; divanov@med.miami.edu.

Supplementary Data S3: ChIP-seq data in Integrated Genome Browser to visually verify the chromatin state in promoters of studied human genes, whose state was not clear

AHR

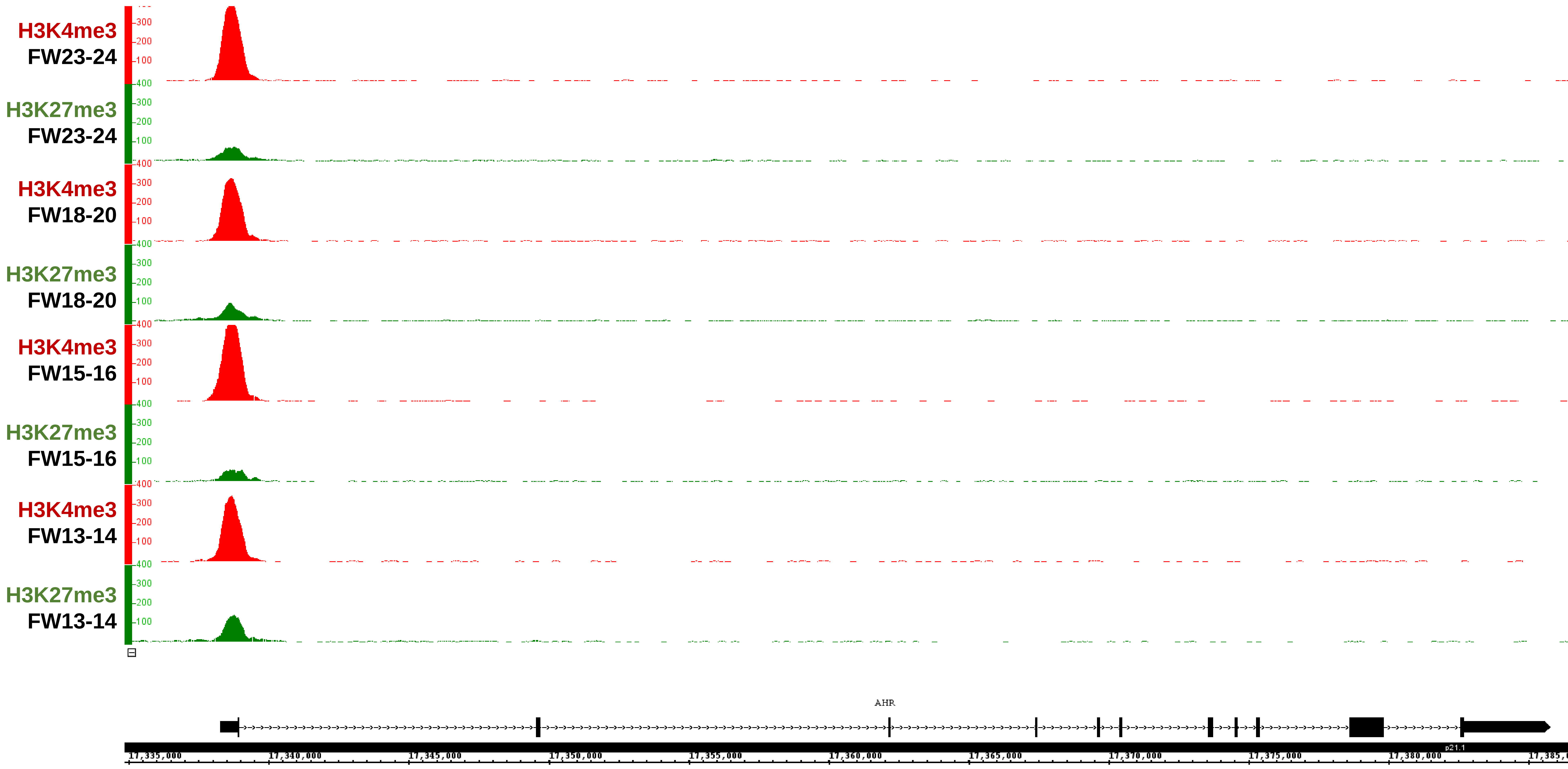

H3K4me3 FW23-24
H3K27me3 FW23-24
H3K4me3 FW18-20
H3K27me3 FW18-20
H3K4me3 FW15-16
H3K27me3 FW15-16
H3K4me3 FW13-14
H3K27me3 FW13-14
300
200
100
400
AHR
17,335,000
17,340,000
17,345,000
17,350,000
17,355,000
17,360,000
17,365,000
17,370,000
17,375,000
17,380,000
p21.1

ATP8A2

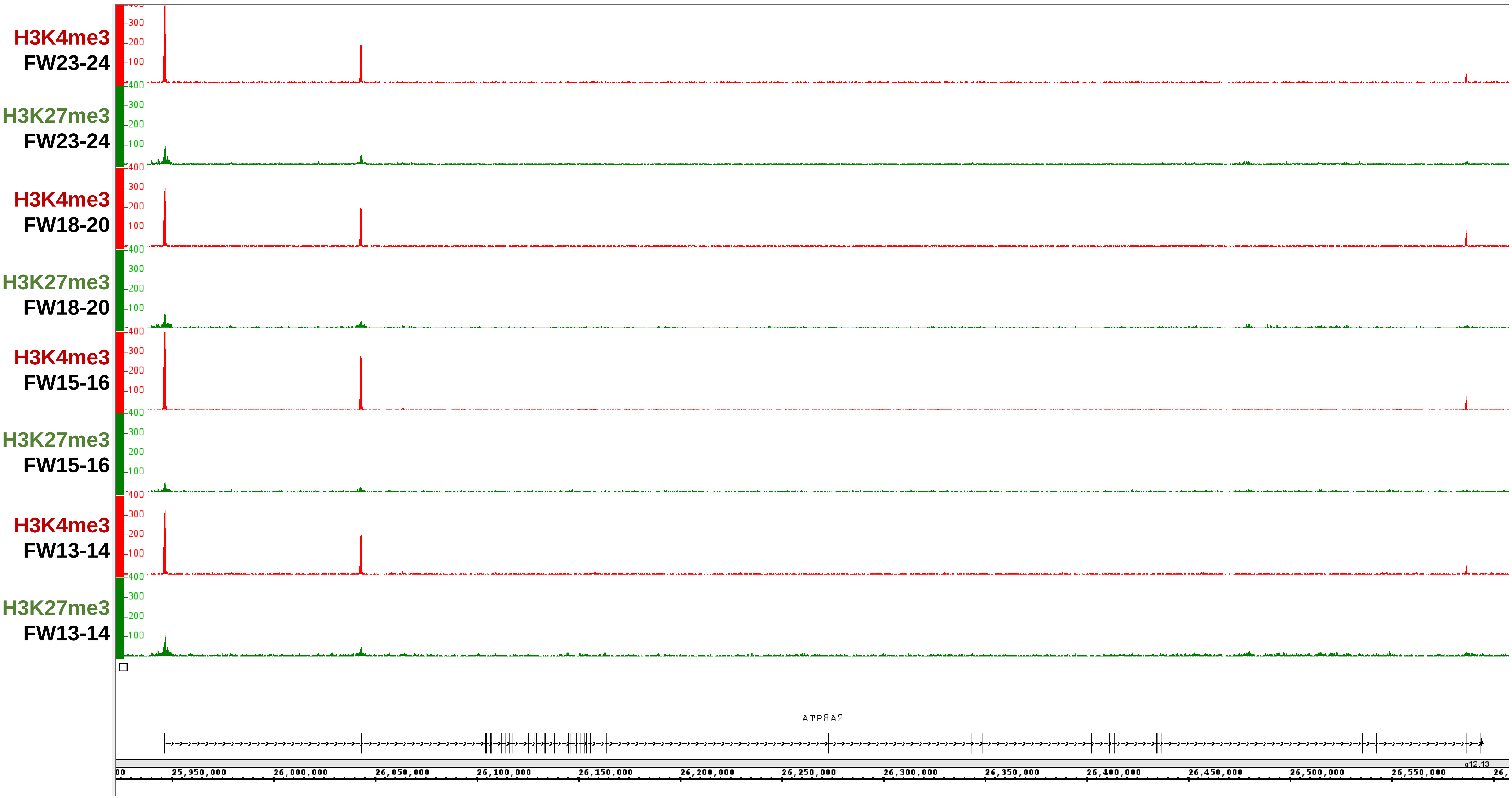

H3K4me3
FW23-24
H3K27me3
FW23-24
H3K4me3
FW18-20
H3K27me3
FW18-20
H3K4me3
FW15-16
H3K27me3
FW15-16
H3K4me3
FW13-14
H3K27me3
FW13-14
300
200
100
400
ATP8A2
25,950,000
26,000,000
26,050,000
26,100,000
26,150,000
26,200,000
26,250,000
26,300,000
26,350,000
26,400,000
26,450,000
26,500,000
26,550,000
q12.13

CDHR1

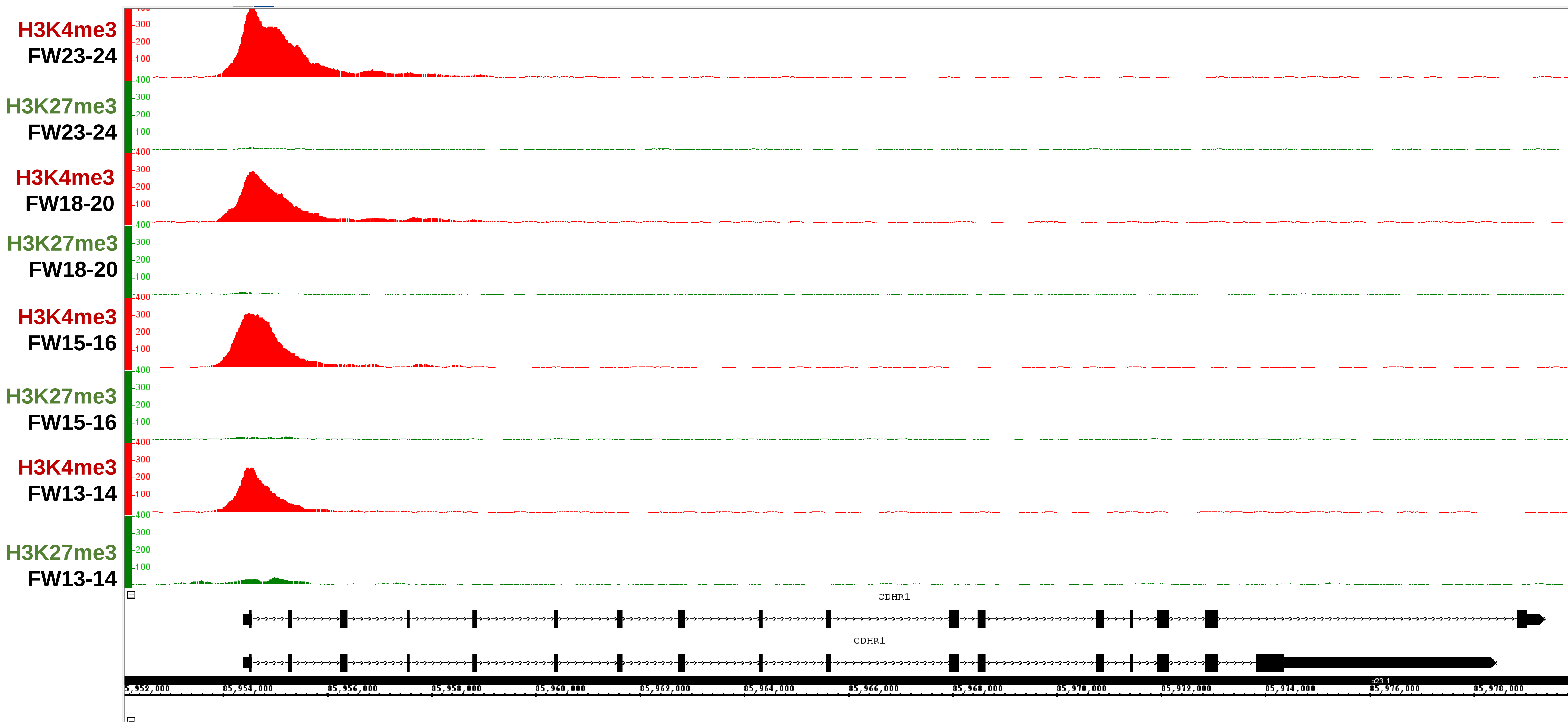
H3K4me3 FW23-24
H3K27me3 FW23-24
H3K4me3 FW18-20
H3K27me3 FW18-20
H3K4me3 FW15-16
H3K27me3 FW15-16
H3K4me3 FW13-14
H3K27me3 FW13-14
300
200
100
400
CDHR1
CDHR1
85,954,000
85,956,000
85,958,000
85,960,000
85,962,000
85,964,000
85,966,000
85,968,000
85,970,000
85,972,000
85,974,000
85,976,000
85,978,000

EGFLAM

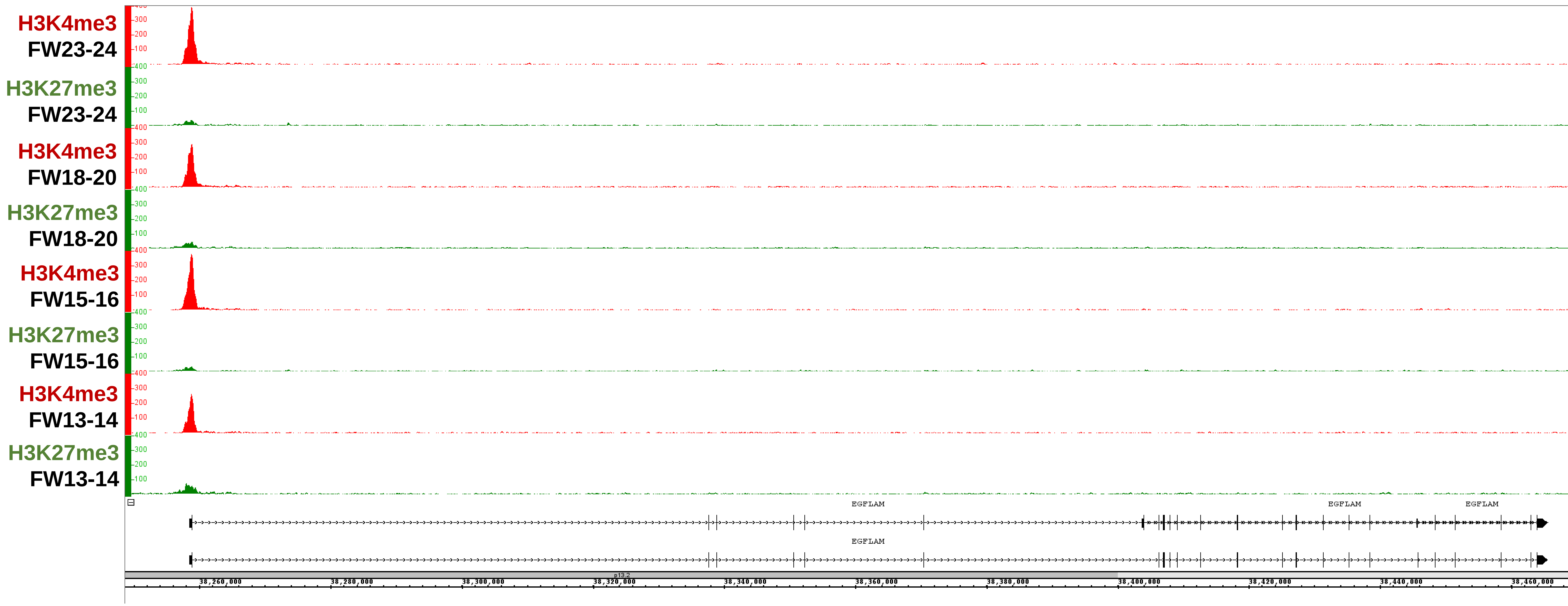
H3K4me3
FW23-24
H3K27me3
FW23-24
H3K4me3
FW18-20
H3K27me3
FW18-20
H3K4me3
FW15-16
H3K27me3
FW15-16
H3K4me3
FW13-14
H3K27me3
FW13-14
EGFLAM
EGFLAM
EGFLAM
EGFLAM
38,260,000
38,280,000
38,300,000
38,320,000
38,340,000
38,360,000
38,380,000
38,400,000
38,420,000
38,440,000
38,460,000

GDF6

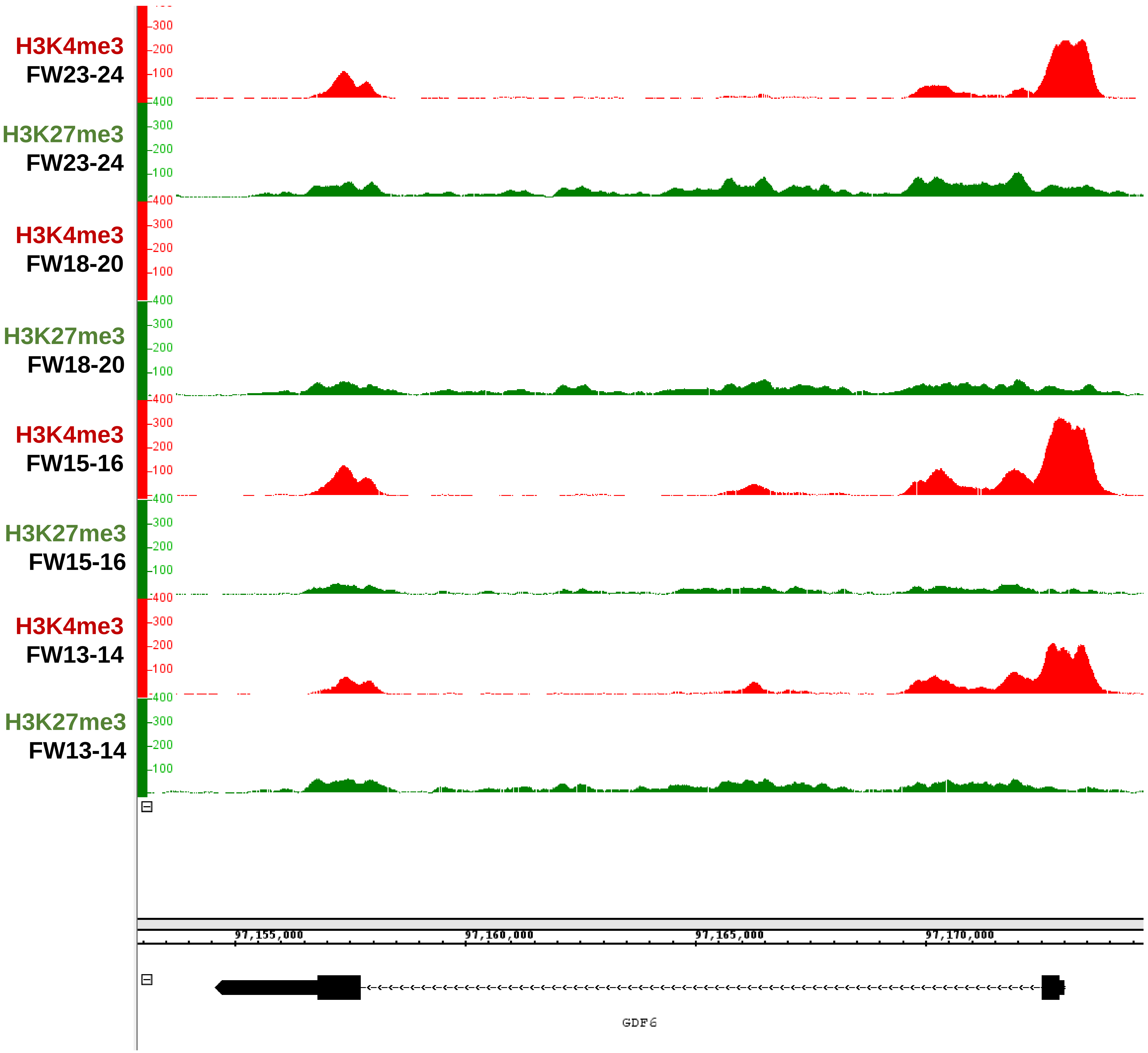
H3K4me3
FW23-24
H3K27me3
FW23-24
H3K4me3
FW18-20
H3K27me3
FW18-20
H3K4me3
FW15-16
H3K27me3
FW15-16
H3K4me3
FW13-14
H3K27me3
FW13-14
300
200
100
400
97,155,000
97,160,000
97,165,000
97,170,000
GDF6

GPR88

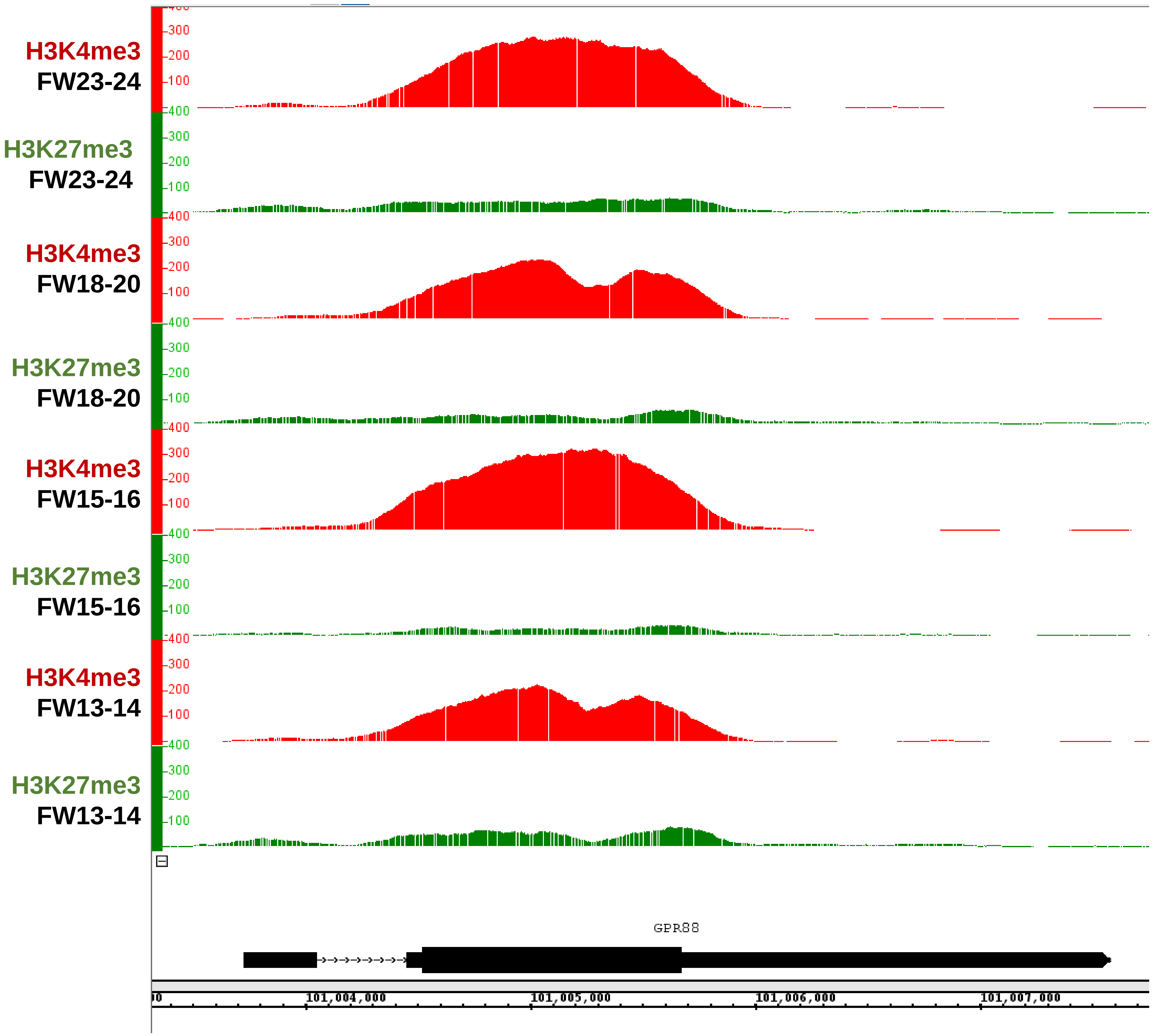
H3K4me3
FW23-24
H3K27me3
FW23-24
H3K4me3
FW18-20
H3K27me3
FW18-20
H3K4me3
FW15-16
H3K27me3
FW15-16
H3K4me3
FW13-14
H3K27me3
FW13-14
400
300
200
100
GPR88
101,004,000
101,005,000
101,006,000
101,007,000

GUCY2D

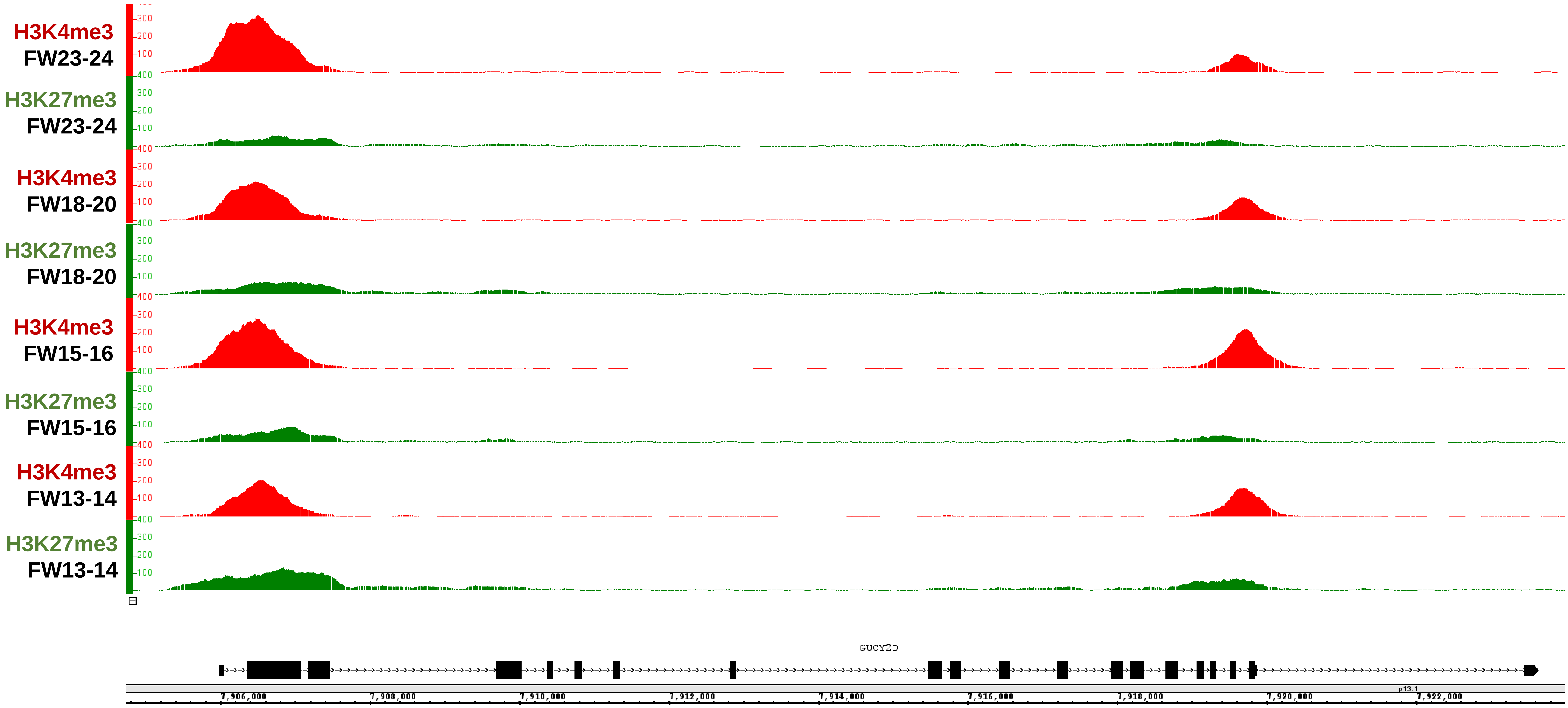

H3K4me3
FW23-24
H3K27me3
FW23-24
H3K4me3
FW18-20
H3K27me3
FW18-20
H3K4me3
FW15-16
H3K27me3
FW15-16
H3K4me3
FW13-14
H3K27me3
FW13-14
GUCY2D
7,906,000
7,908,000
7,910,000
7,912,000
7,914,000
7,916,000
7,918,000
7,920,000
7,922,000

NXNL1

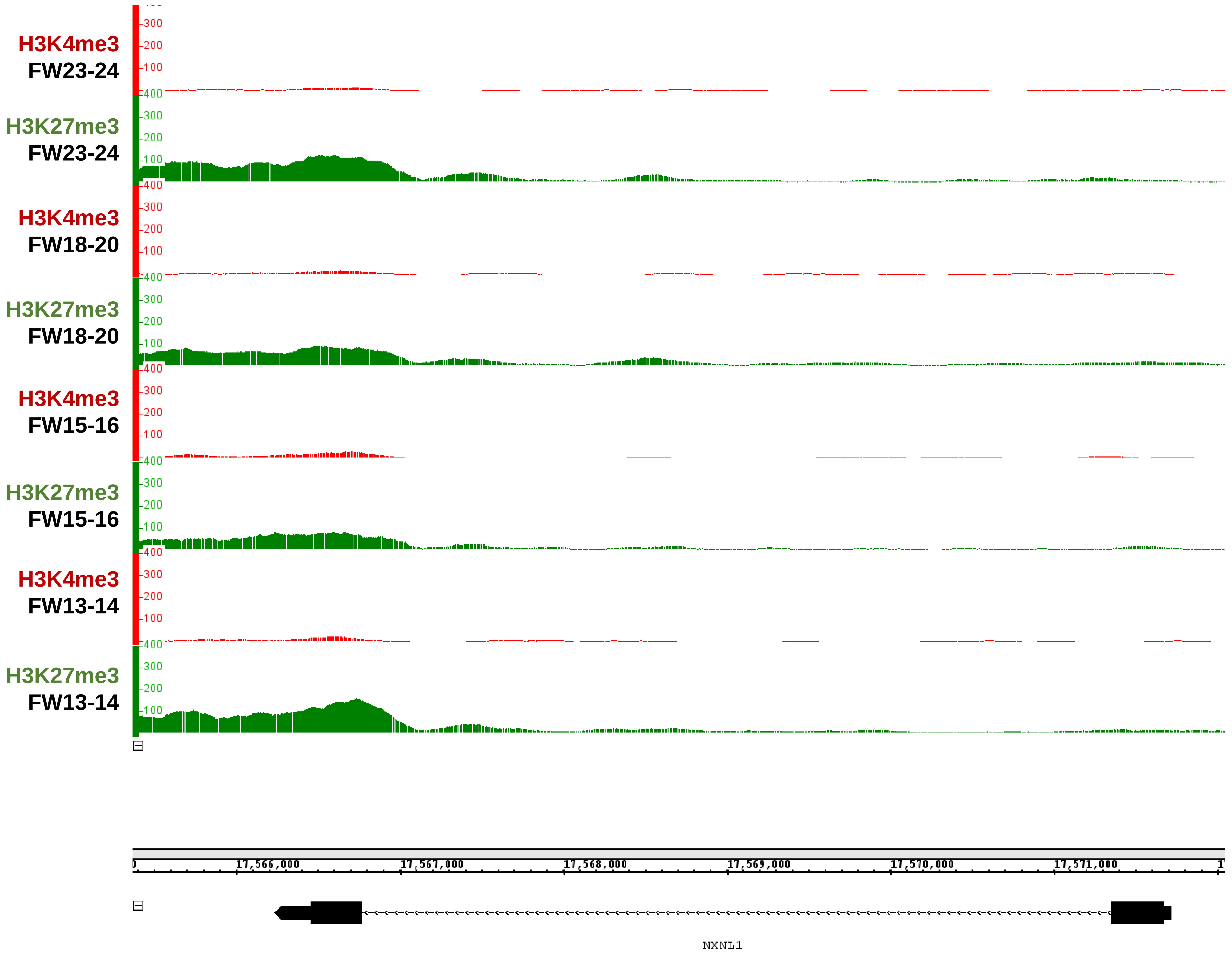
H3K4me3
FW23-24
H3K27me3
FW23-24
H3K4me3
FW18-20
H3K27me3
FW18-20
H3K4me3
FW15-16
H3K27me3
FW15-16
H3K4me3
FW13-14
H3K27me3
FW13-14
400
300
200
100
17,566,000
17,567,000
17,568,000
17,569,000
17,570,000
17,571,000
NXNL1

PITPNM3

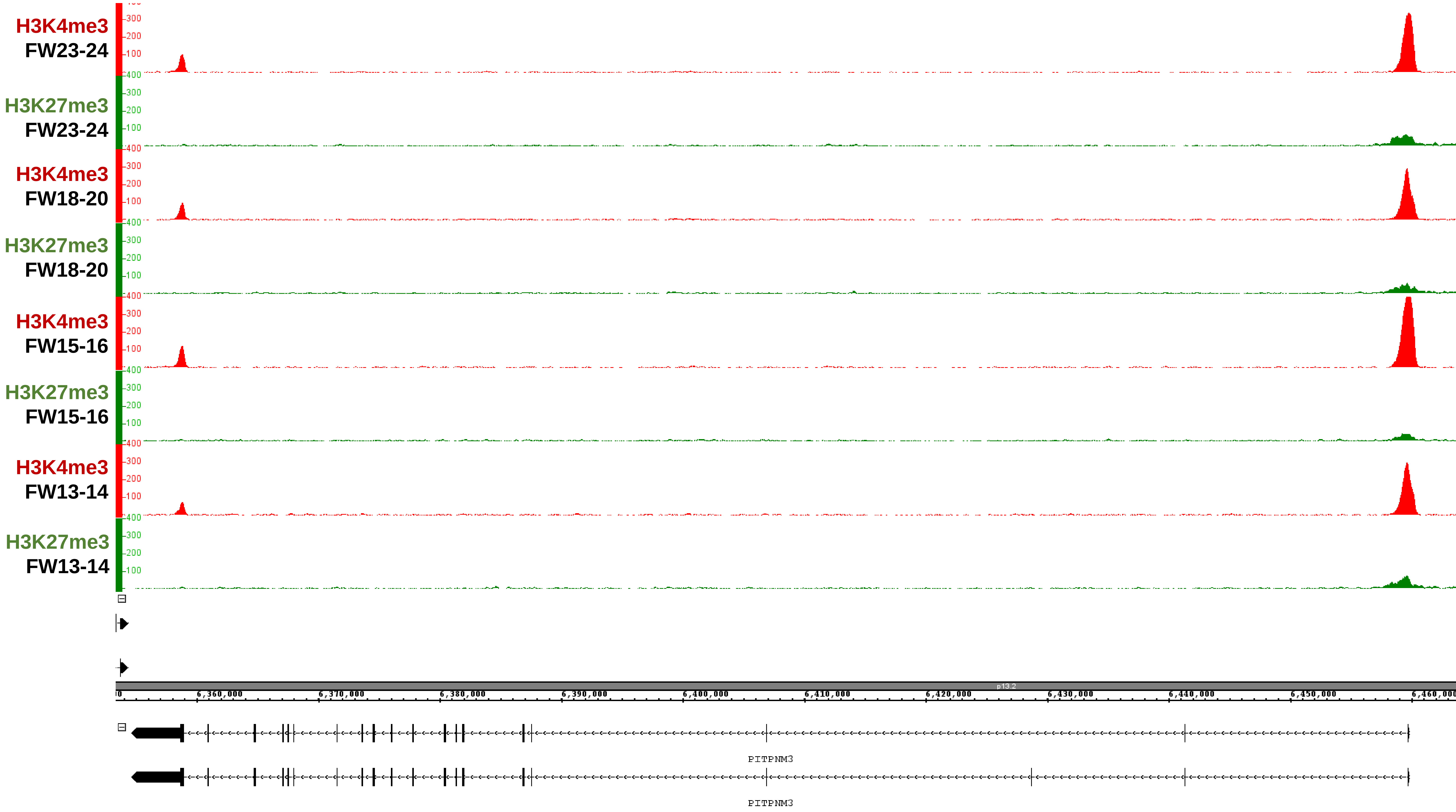

H3K4me3
FW23-24
H3K27me3
FW23-24
H3K4me3
FW18-20
H3K27me3
FW18-20
H3K4me3
FW15-16
H3K27me3
FW15-16
H3K4me3
FW13-14
H3K27me3
FW13-14
6,360,000
6,370,000
6,380,000
6,390,000
6,400,000
6,410,000
6,420,000
6,430,000
6,440,000
6,450,000
PITPNM3
PITPNM3

RAPGEF4

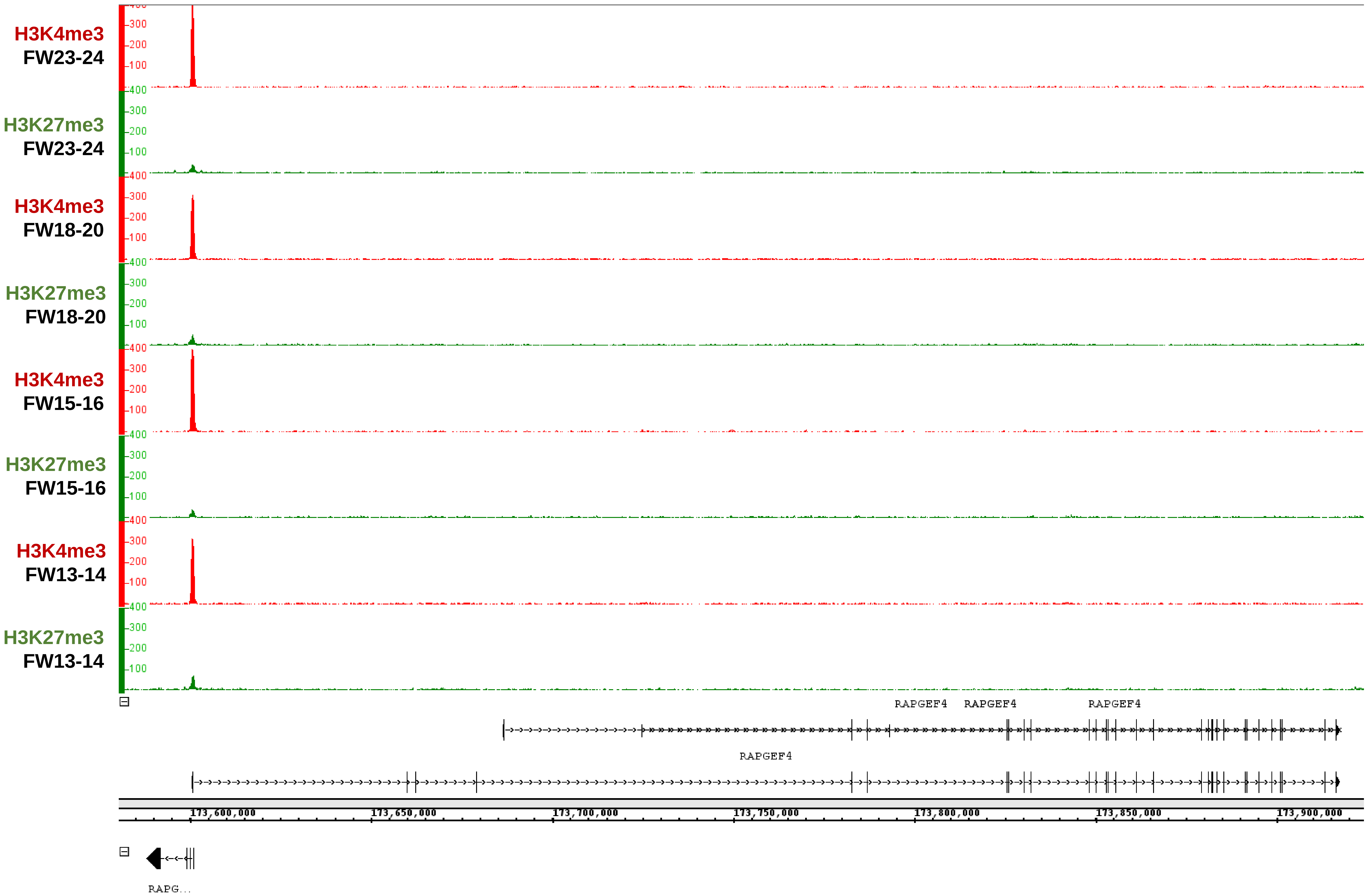
H3K4me3
FW23-24
H3K27me3
FW23-24
H3K4me3
FW18-20
H3K27me3
FW18-20
H3K4me3
FW15-16
H3K27me3
FW15-16
H3K4me3
FW13-14
H3K27me3
FW13-14
400
300
200
100
RAPGEF4
RAPGEF4
RAPGEF4
RAPGEF4
173,600,000
173,650,000
173,700,000
173,750,000
173,800,000
173,850,000
173,900,000
RAPG...

RIMS2

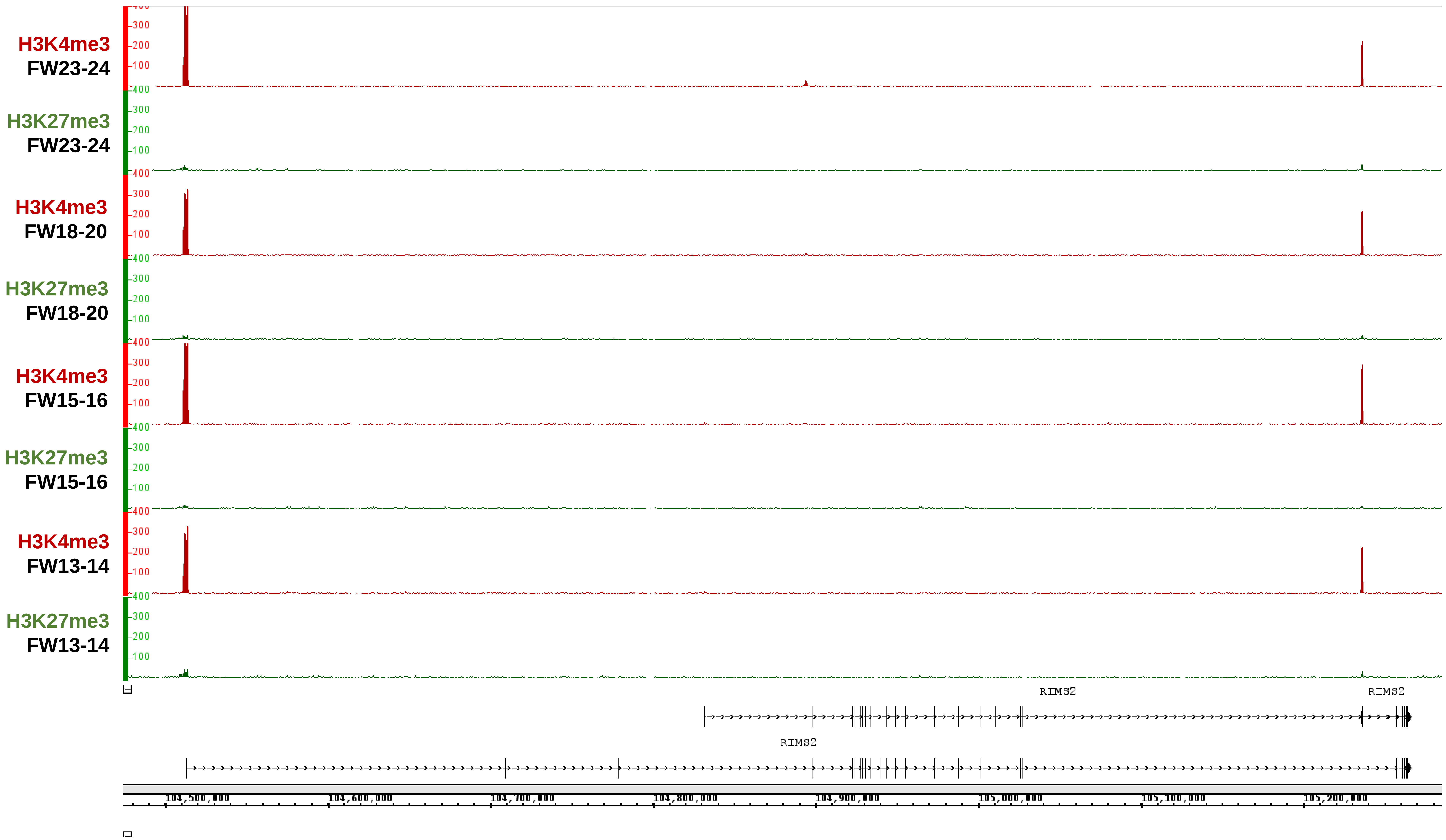
H3K4me3 FW23-24
H3K27me3 FW23-24
H3K4me3 FW18-20
H3K27me3 FW18-20
H3K4me3 FW15-16
H3K27me3 FW15-16
H3K4me3 FW13-14
H3K27me3 FW13-14
400
300
200
100
RIMS2
RIMS2
RIMS2
104,500,000
104,600,000
104,700,000
104,800,000
104,900,000
105,000,000
105,100,000
105,200,000

SEMA4A

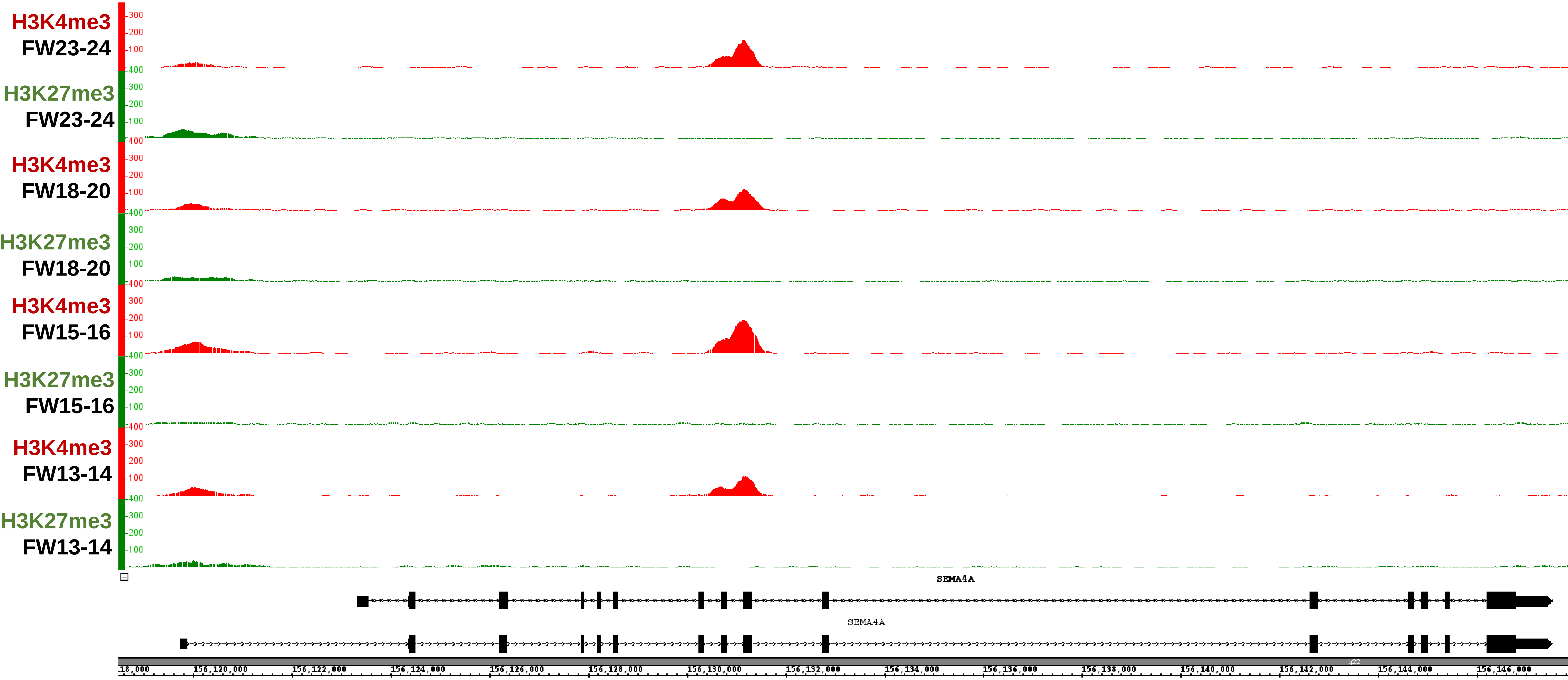
H3K4me3
FW23-24
H3K27me3
FW23-24
H3K4me3
FW18-20
H3K27me3
FW18-20
H3K4me3
FW15-16
H3K27me3
FW15-16
H3K4me3
FW13-14
H3K27me3
FW13-14
300
200
100
400
SEMA4A
SEMA4A
156,120,000
156,122,000
156,124,000
156,126,000
156,128,000
156,130,000
156,132,000
156,134,000
156,136,000
156,138,000
156,140,000
156,142,000
156,144,000
156,146,000

SLC7A14

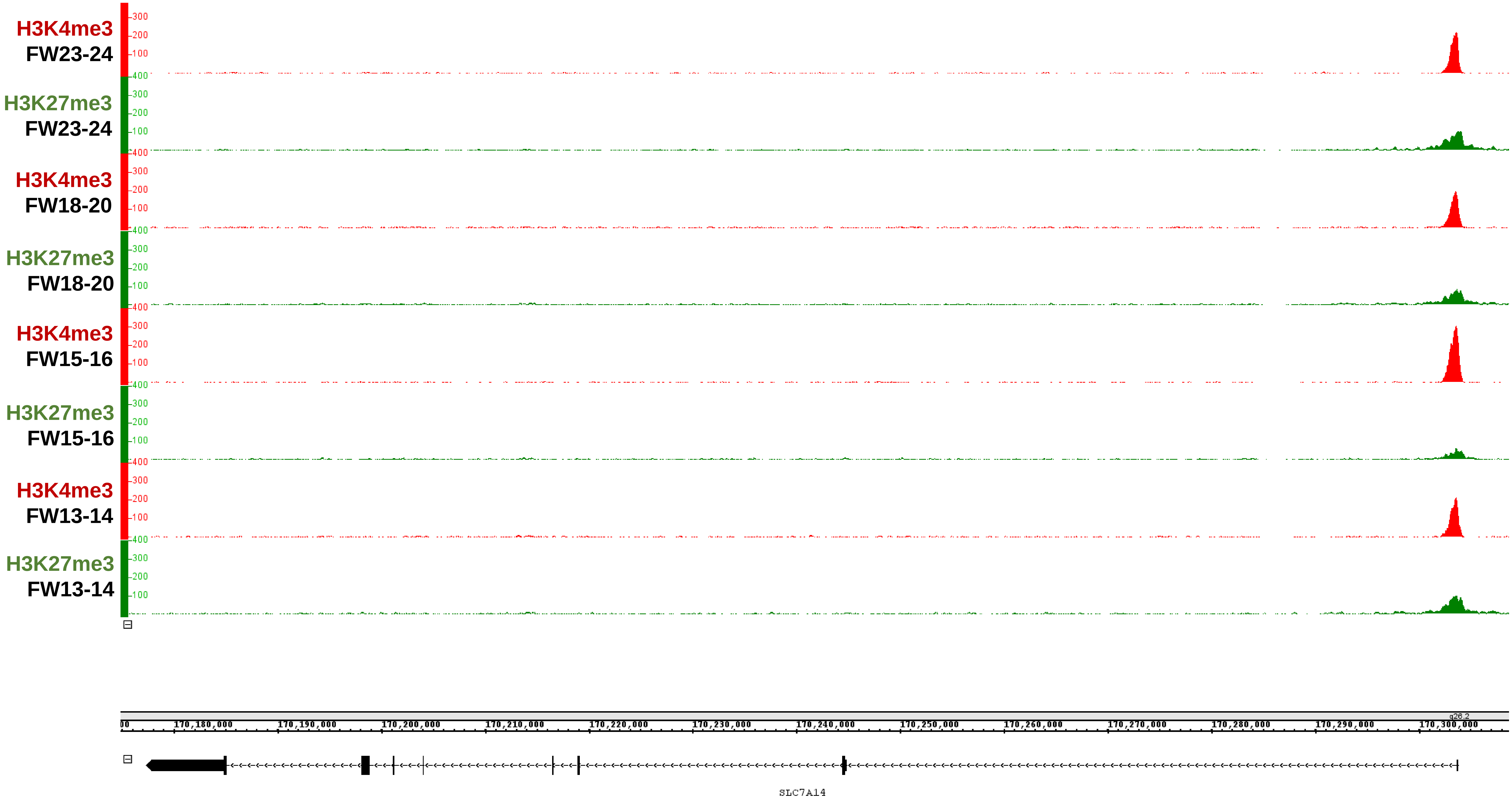
H3K4me3
FW23-24
H3K27me3
FW23-24
H3K4me3
FW18-20
H3K27me3
FW18-20
H3K4me3
FW15-16
H3K27me3
FW15-16
H3K4me3
FW13-14
H3K27me3
FW13-14
300
200
100
170,180,000
170,190,000
170,200,000
170,210,000
170,220,000
170,230,000
170,240,000
170,250,000
170,260,000
170,270,000
170,280,000
170,290,000
170,300,000
SLC7A14

SLC24A2

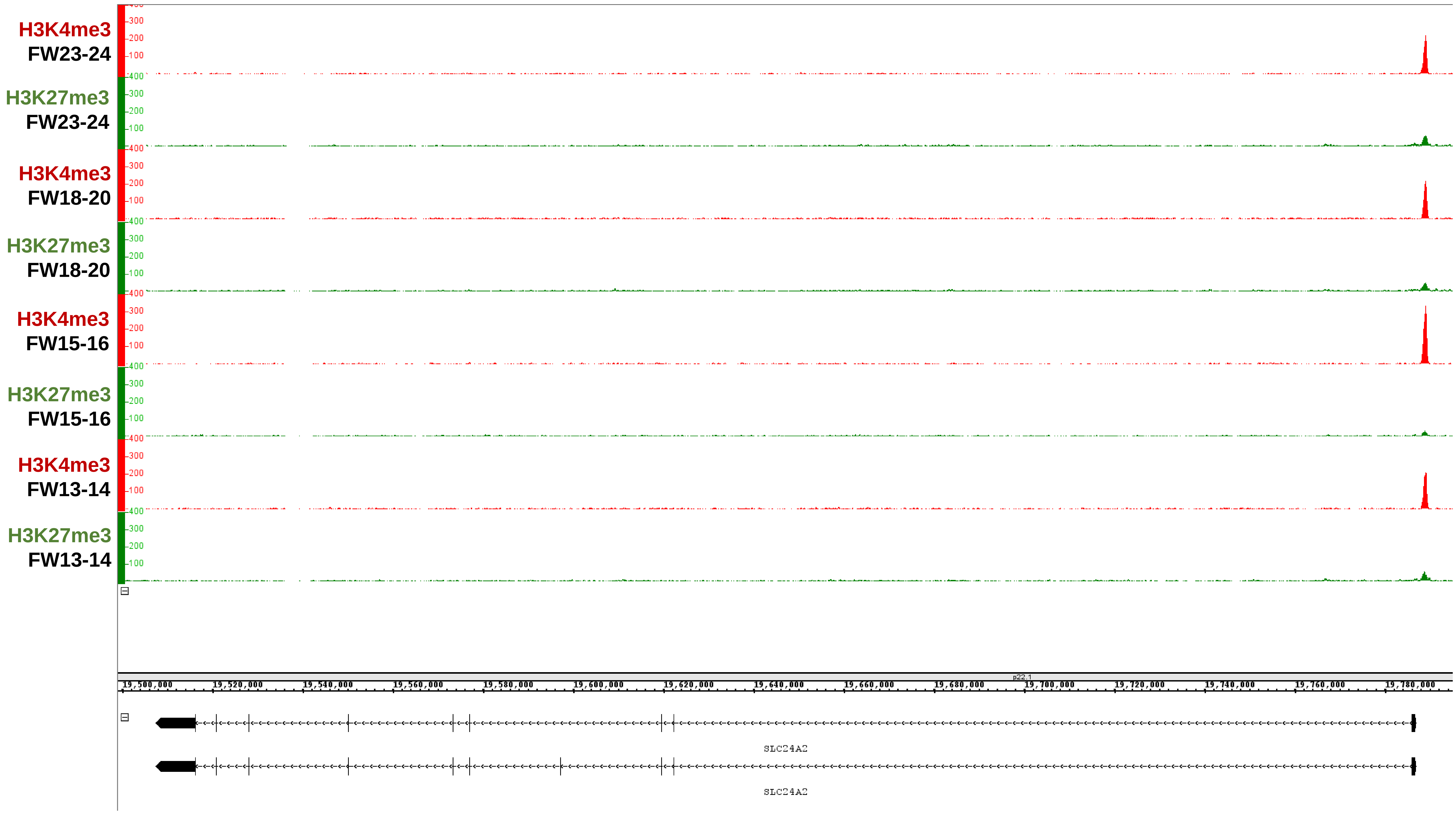
H3K4me3
FW23-24
H3K27me3
FW23-24
H3K4me3
FW18-20
H3K27me3
FW18-20
H3K4me3
FW15-16
H3K27me3
FW15-16
H3K4me3
FW13-14
H3K27me3
FW13-14
19,500,000
19,520,000
19,540,000
19,560,000
19,580,000
19,600,000
19,620,000
19,640,000
19,660,000
19,680,000
19,700,000
19,720,000
19,740,000
19,760,000
19,780,000
SLC24A2
SLC24A2

TRPC3

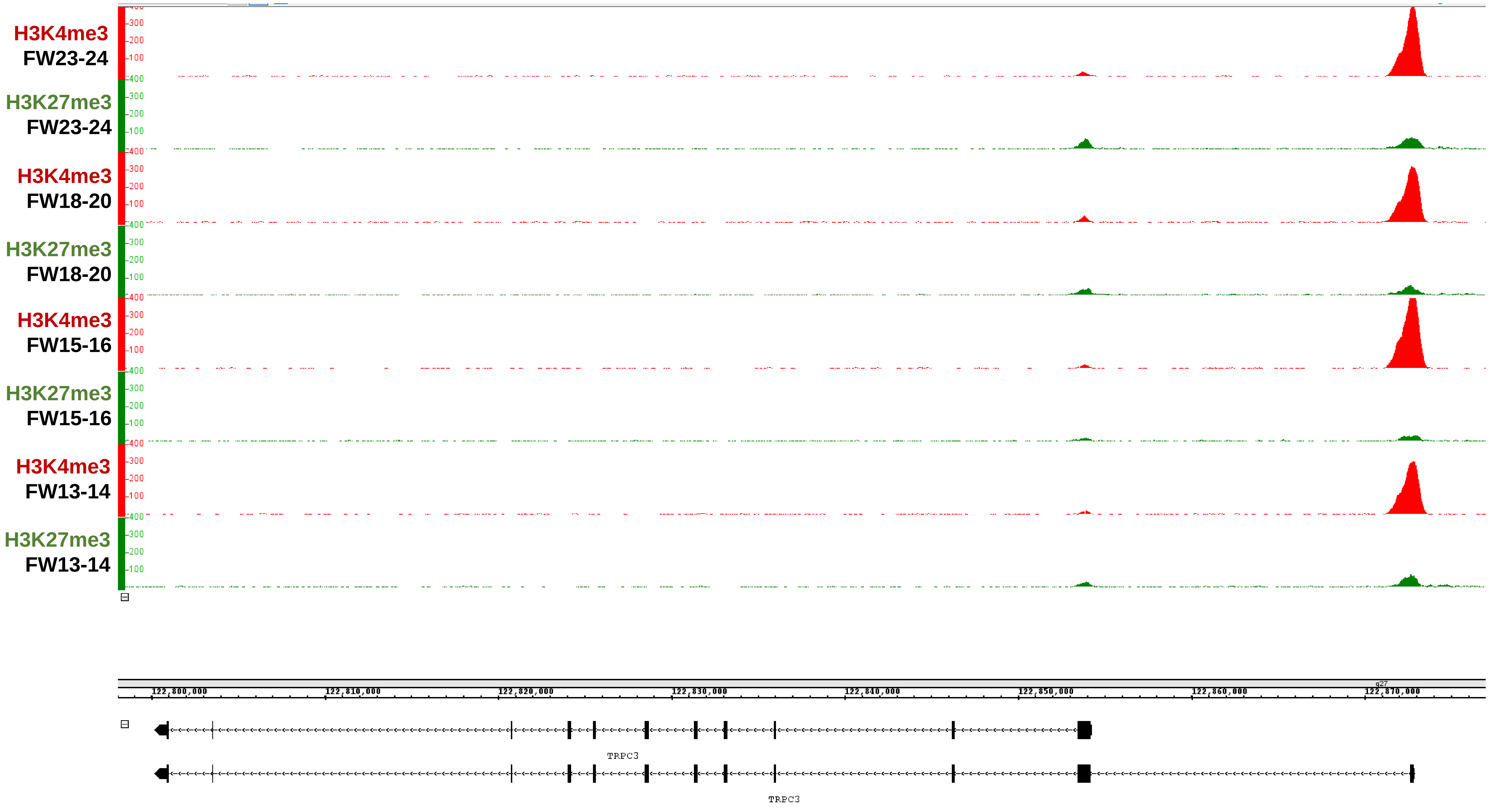

H3K4me3 FW23-24
H3K27me3 FW23-24
H3K4me3 FW18-20
H3K27me3 FW18-20
H3K4me3 FW15-16
H3K27me3 FW15-16
H3K4me3 FW13-14
H3K27me3 FW13-14
300
200
100
400
122,800,000
122,810,000
122,820,000
122,830,000
122,840,000
122,850,000
122,860,000
122,870,000
q27
TRPC3
TRPC3

USH1G

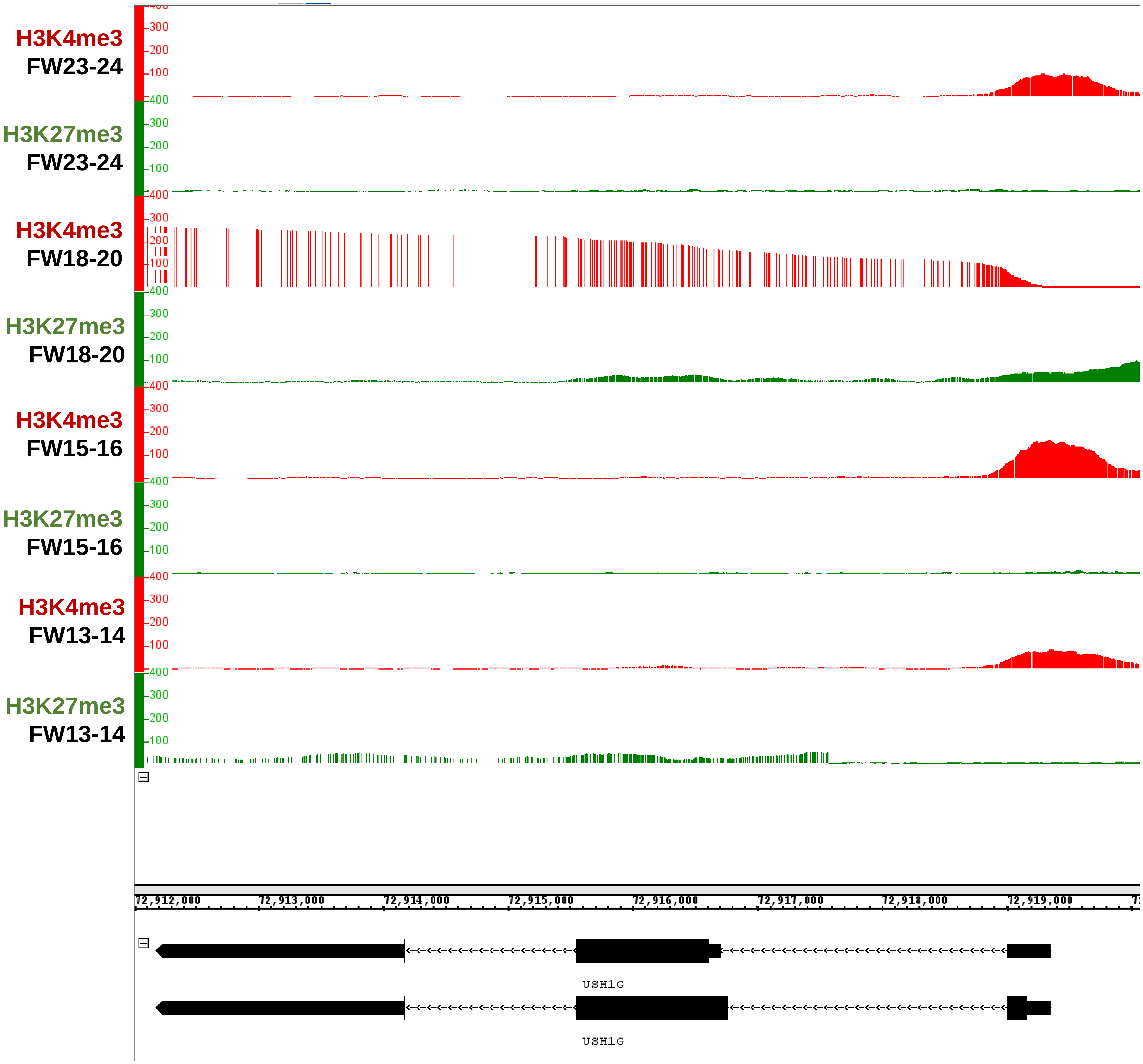
H3K4me3
FW23-24
H3K27me3
FW23-24
H3K4me3
FW18-20
H3K27me3
FW18-20
H3K4me3
FW15-16
H3K27me3
FW15-16
H3K4me3
FW13-14
H3K27me3
FW13-14
72,912,000
72,913,000
72,914,000
72,915,000
72,916,000
72,917,000
72,918,000
72,919,000
USH1G
USH1G

CNGA3

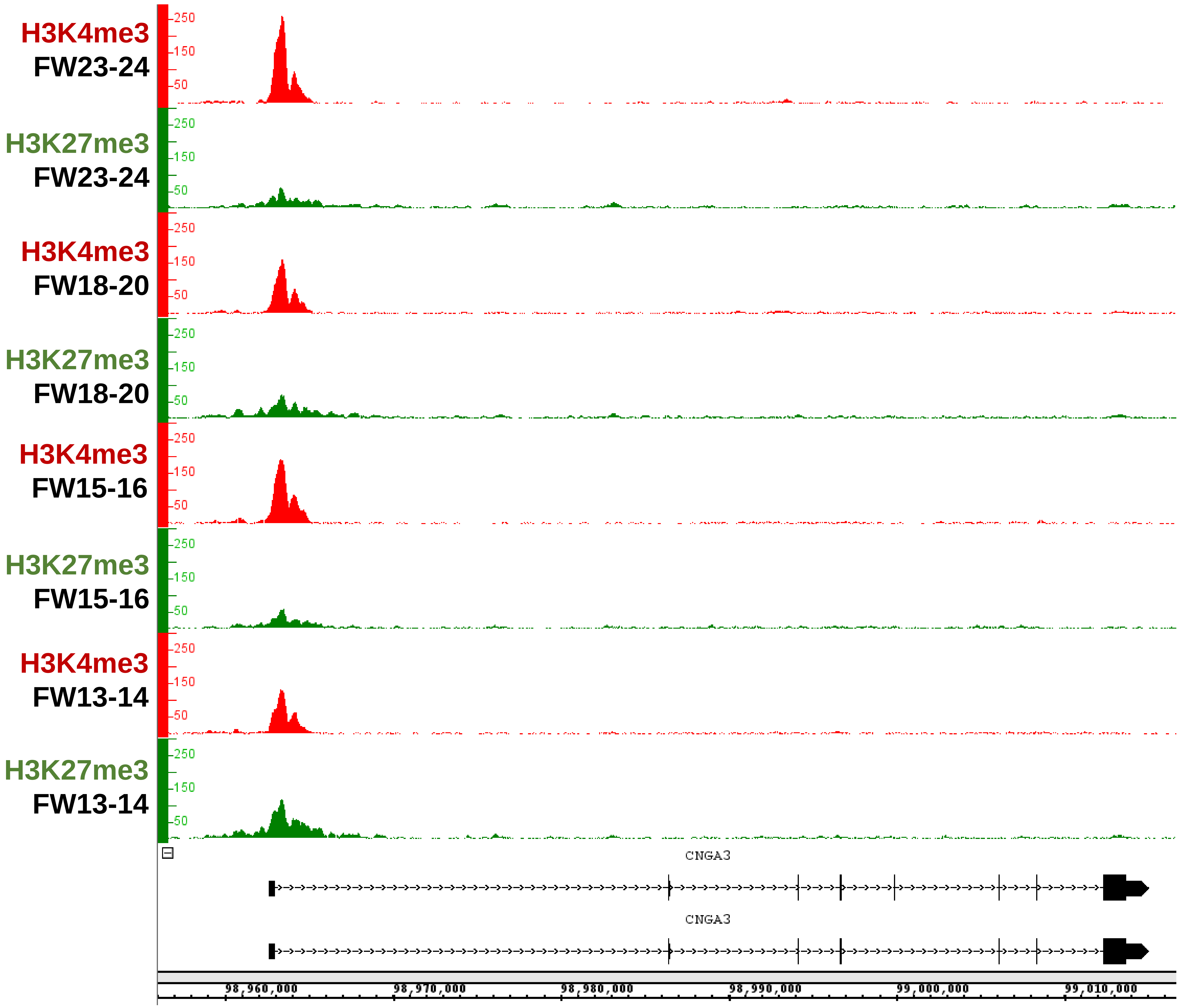

H3K4me3
FW23-24
H3K27me3
FW23-24
H3K4me3
FW18-20
H3K27me3
FW18-20
H3K4me3
FW15-16
H3K27me3
FW15-16
H3K4me3
FW13-14
H3K27me3
FW13-14
250
150
50
CNGA3
CNGA3
98,960,000
98,970,000
98,980,000
98,990,000
99,000,000
99,010,000

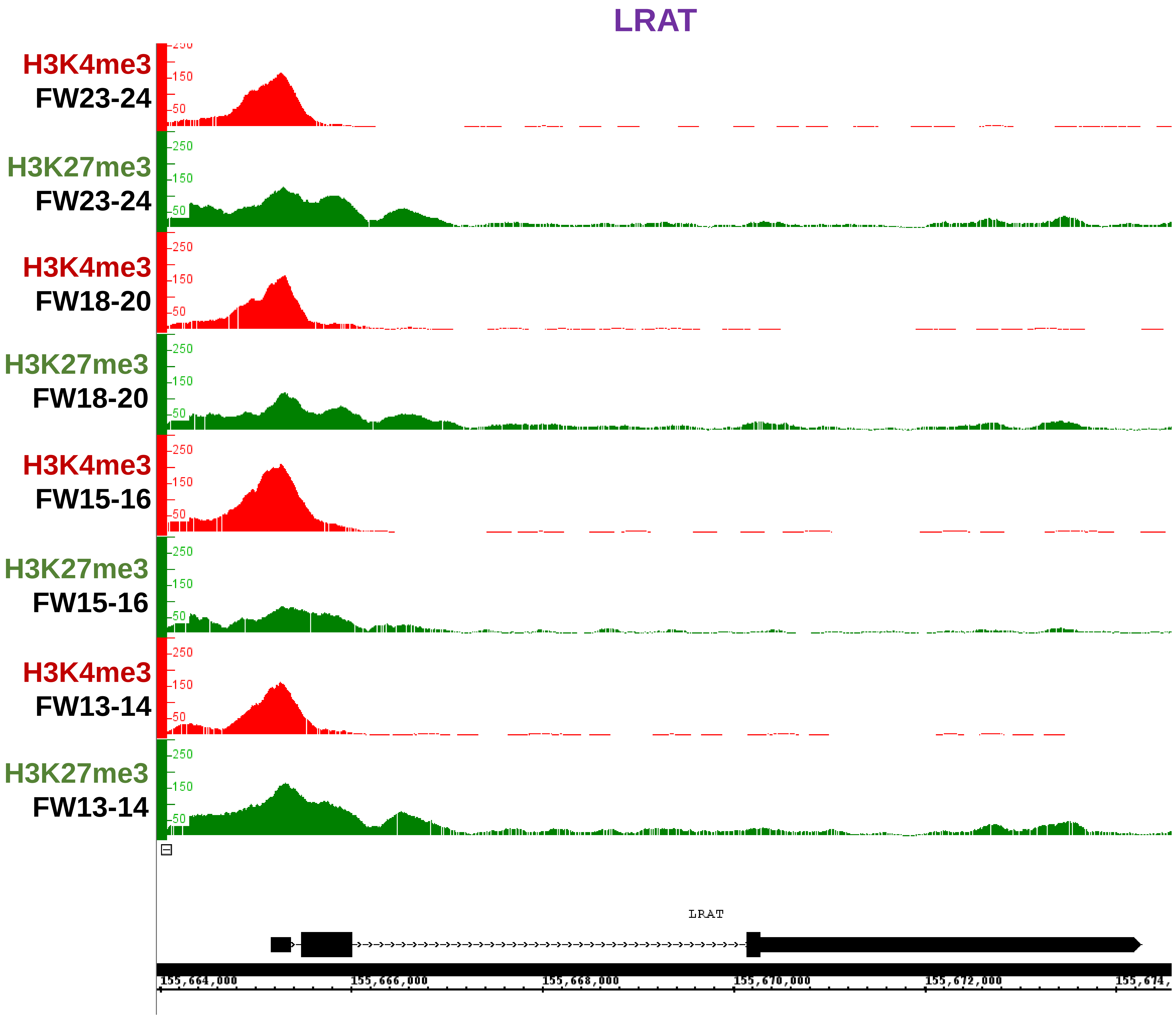
LRAT
H3K4me3 FW23-24
H3K27me3 FW23-24
H3K4me3 FW18-20
H3K27me3 FW18-20
H3K4me3 FW15-16
H3K27me3 FW15-16
H3K4me3 FW13-14
H3K27me3 FW13-14
250
150
50
LRAT
155,664,000
155,666,000
155,668,000
155,670,000
155,672,000
155,674,

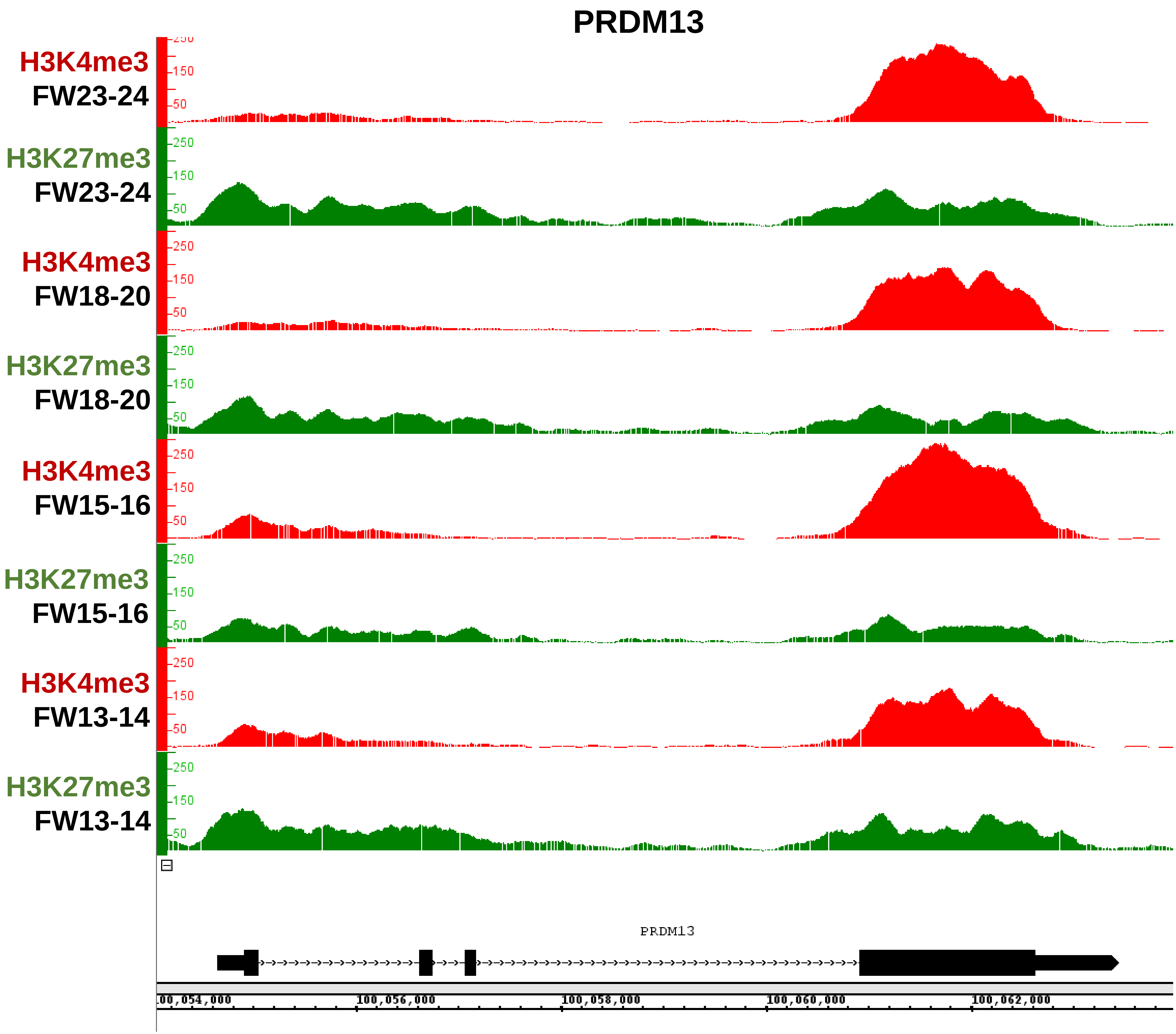
PRDM13
H3K4me3
FW23-24
H3K27me3
FW23-24
H3K4me3
FW18-20
H3K27me3
FW18-20
H3K4me3
FW15-16
H3K27me3
FW15-16
H3K4me3
FW13-14
H3K27me3
FW13-14
250
150
50
PRDM13
00,054,000
100,056,000
100,058,000
100,060,000
100,062,000

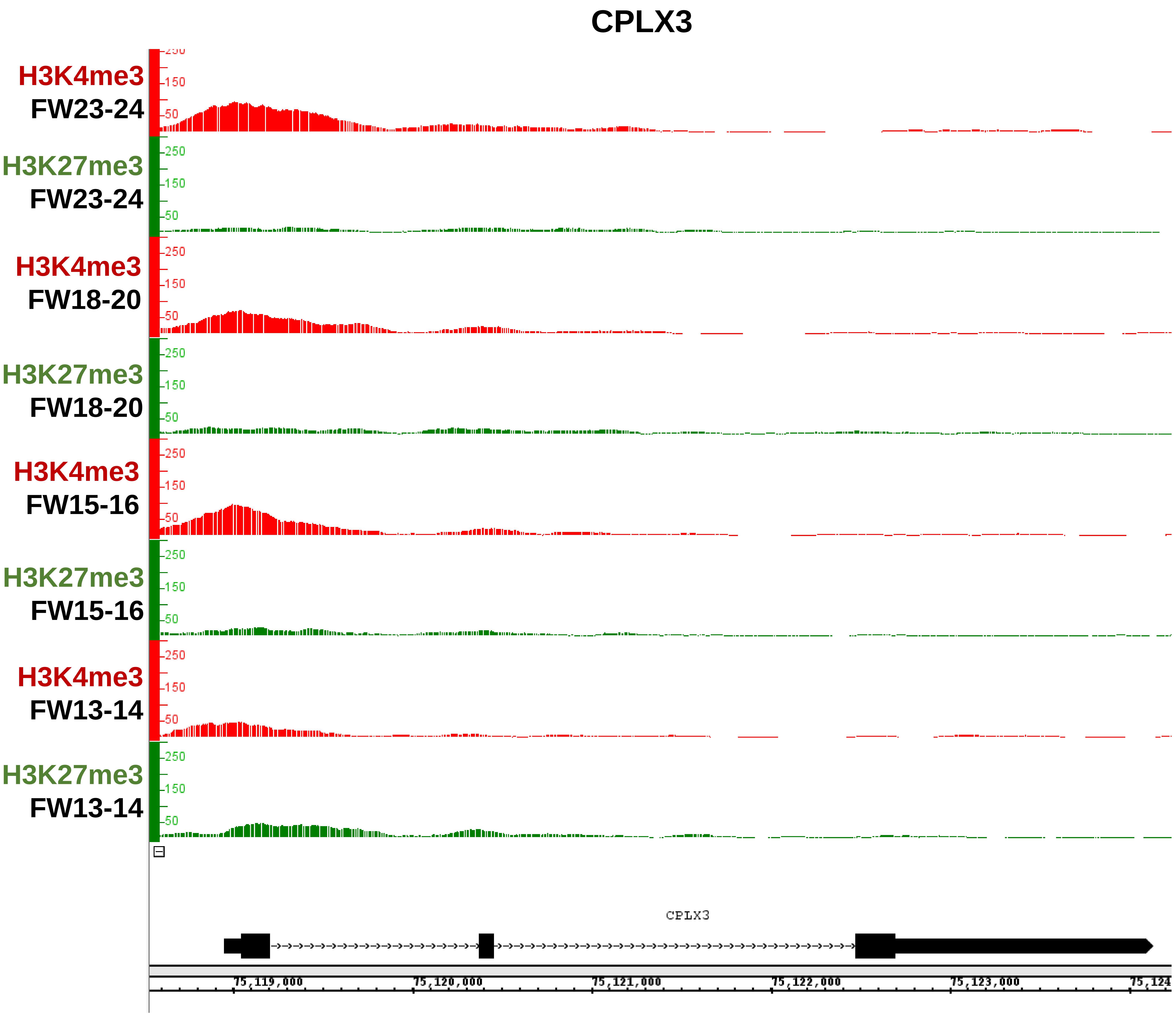
CPLX3
H3K4me3
FW23-24
H3K27me3
FW23-24
H3K4me3
FW18-20
H3K27me3
FW18-20
H3K4me3
FW15-16
H3K27me3
FW15-16
H3K4me3
FW13-14
H3K27me3
FW13-14
250
150
50
CPLX3
75,119,000
75,120,000
75,121,000
75,122,000
75,123,000
75,124

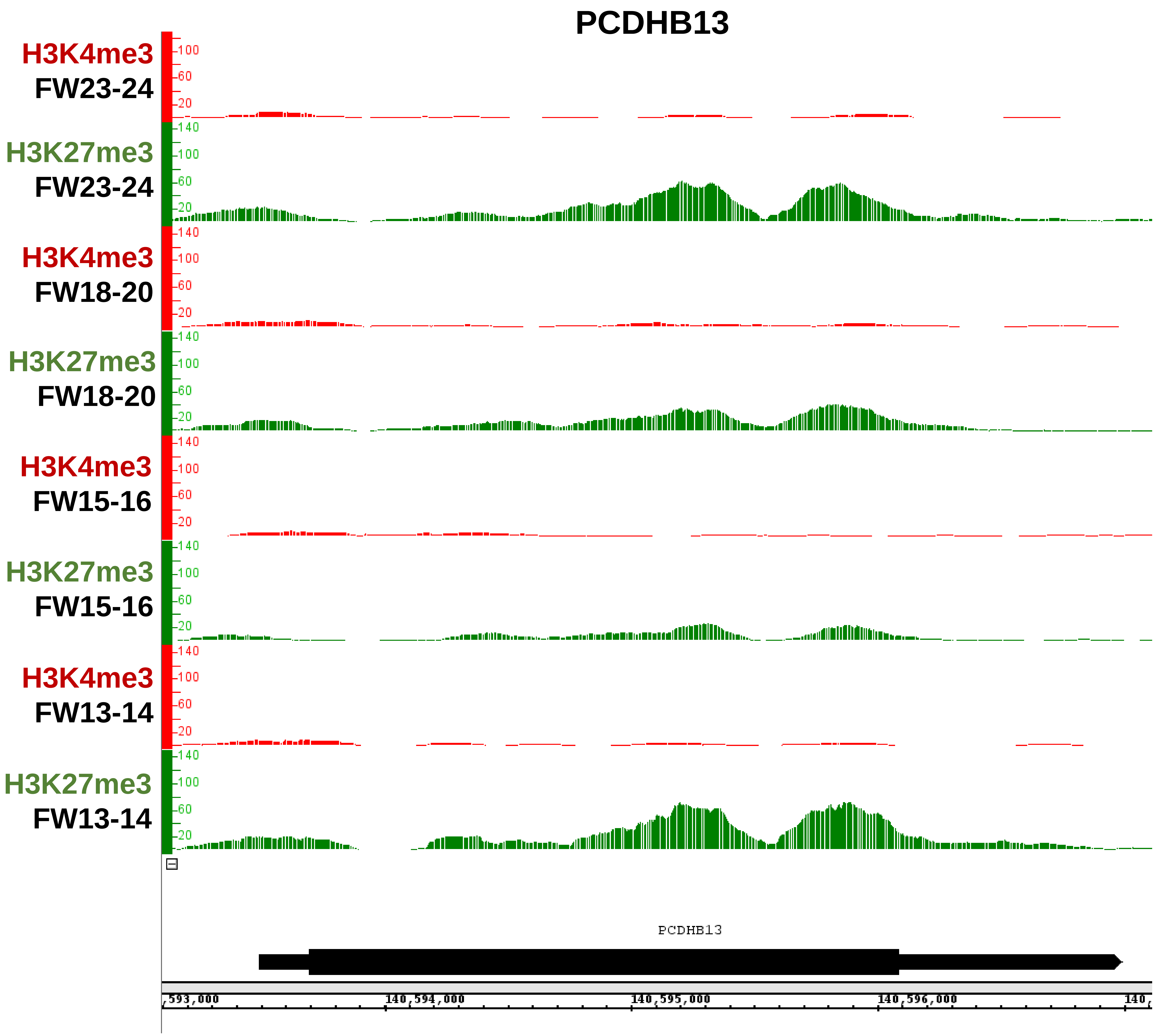

PCDHB13
H3K4me3
FW23-24
H3K27me3
FW23-24
H3K4me3
FW18-20
H3K27me3
FW18-20
H3K4me3
FW15-16
H3K27me3
FW15-16
H3K4me3
FW13-14
H3K27me3
FW13-14
100
60
20
140
PCDHB13
593,000
140,594,000
140,595,000
140,596,000
140,

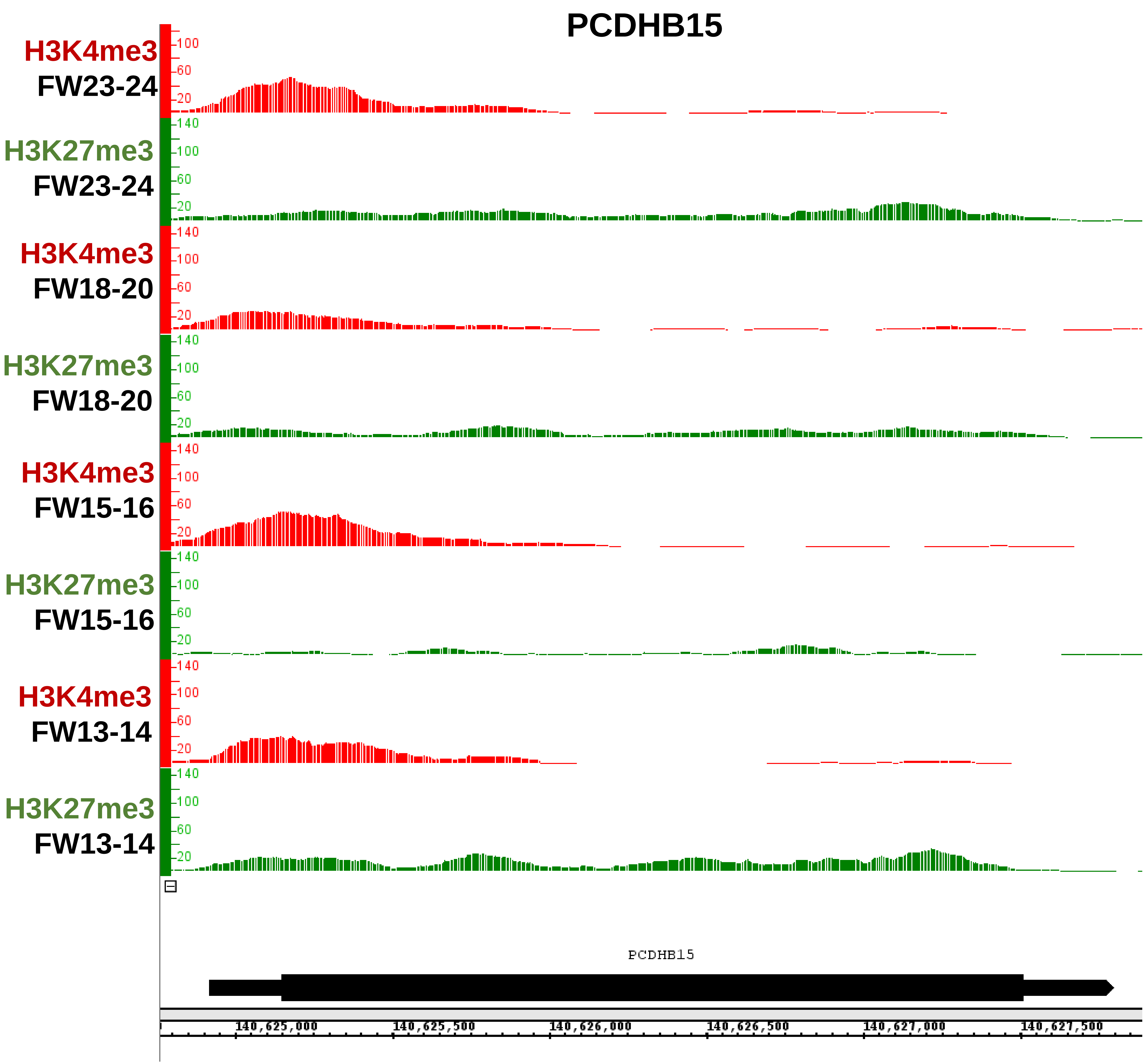
PCDHB15
H3K4me3
FW23-24
H3K27me3
FW23-24
H3K4me3
FW18-20
H3K27me3
FW18-20
H3K4me3
FW15-16
H3K27me3
FW15-16
H3K4me3
FW13-14
H3K27me3
FW13-14
100
60
20
140
PCDHB15
140,625,000
140,625,500
140,626,000
140,626,500
140,627,000
140,627,500

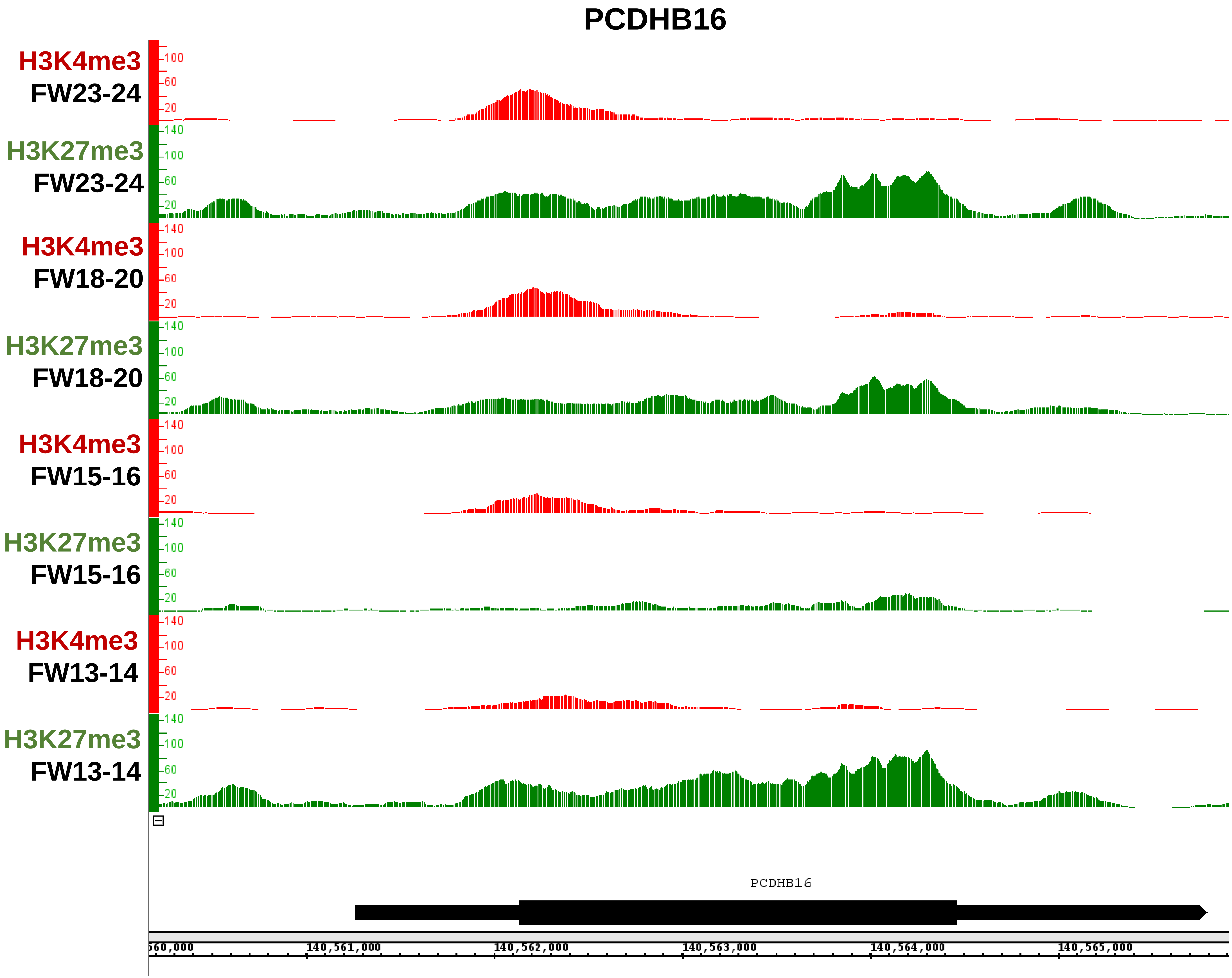
PCDHB16
H3K4me3
FW23-24
H3K27me3
FW23-24
H3K4me3
FW18-20
H3K27me3
FW18-20
H3K4me3
FW15-16
H3K27me3
FW15-16
H3K4me3
FW13-14
H3K27me3
FW13-14
PCDHB16
140,561,000
140,562,000
140,563,000
140,564,000
140,565,000

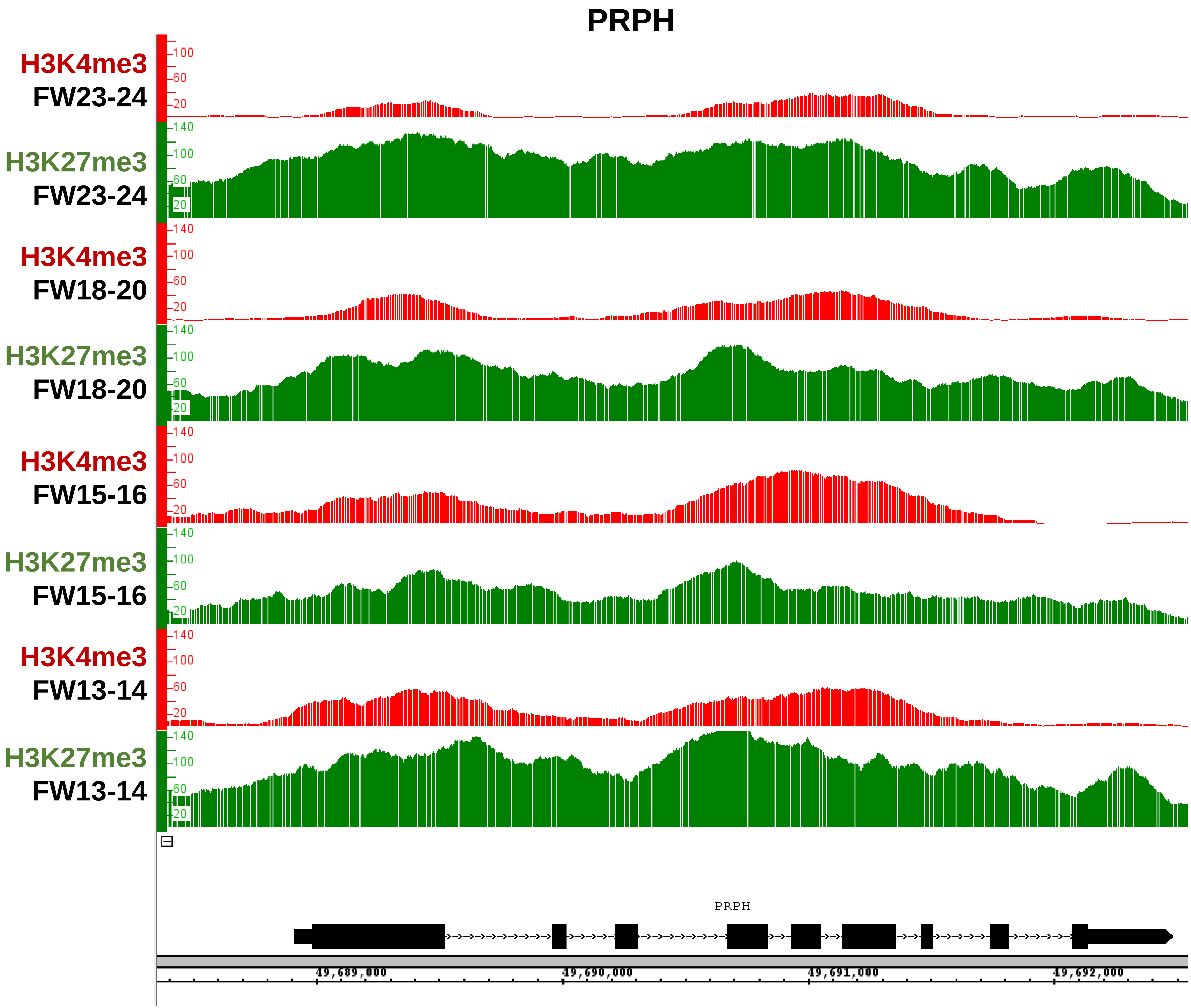
PRPH
H3K4me3
FW23-24
H3K27me3
FW23-24
H3K4me3
FW18-20
H3K27me3
FW18-20
H3K4me3
FW15-16
H3K27me3
FW15-16
H3K4me3
FW13-14
H3K27me3
FW13-14
20
60
100
140
PRPH
49,689,000
49,690,000
49,691,000
49,692,000

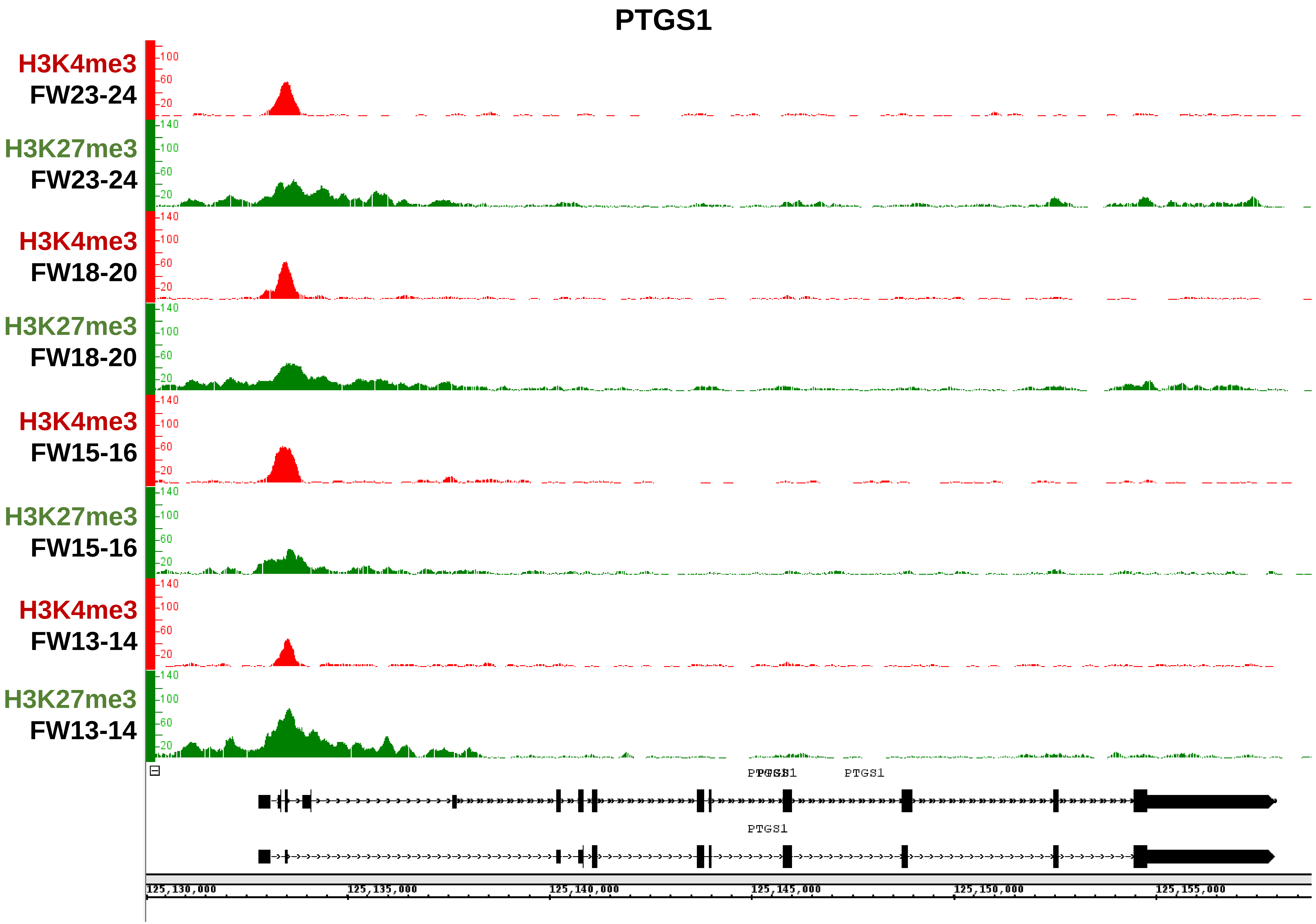
PTGS1
H3K4me3
FW23-24
H3K27me3
FW23-24
H3K4me3
FW18-20
H3K27me3
FW18-20
H3K4me3
FW15-16
H3K27me3
FW15-16
H3K4me3
FW13-14
H3K27me3
FW13-14
PTGS1
PTGS1
125,130,000
125,135,000
125,140,000
125,145,000
125,150,000
125,155,000

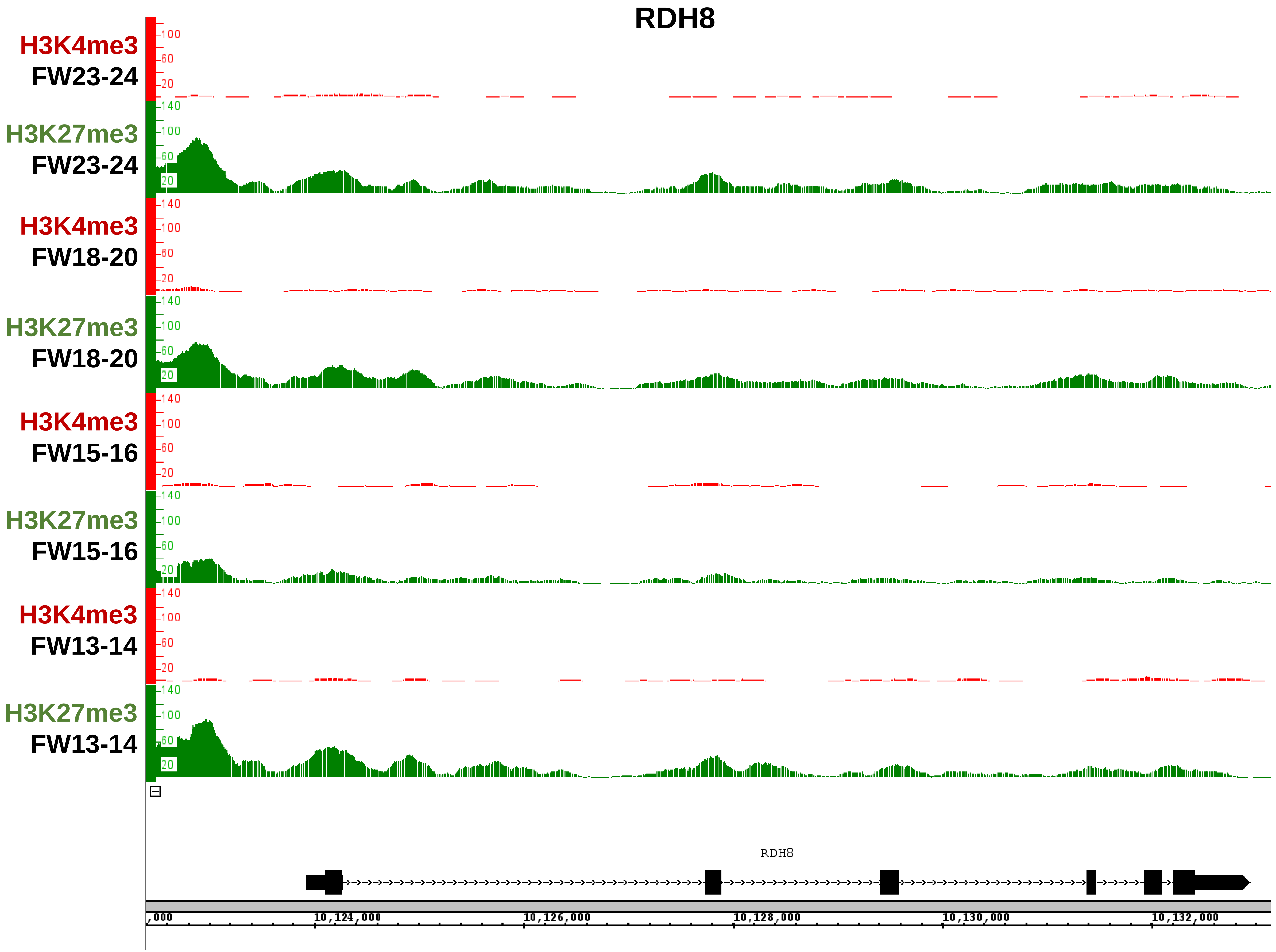

RDH8
H3K4me3
FW23-24
H3K27me3
FW23-24
H3K4me3
FW18-20
H3K27me3
FW18-20
H3K4me3
FW15-16
H3K27me3
FW15-16
H3K4me3
FW13-14
H3K27me3
FW13-14
100
60
20
140
RDH8
,000
10,124,000
10,126,000
10,128,000
10,130,000
10,132,000

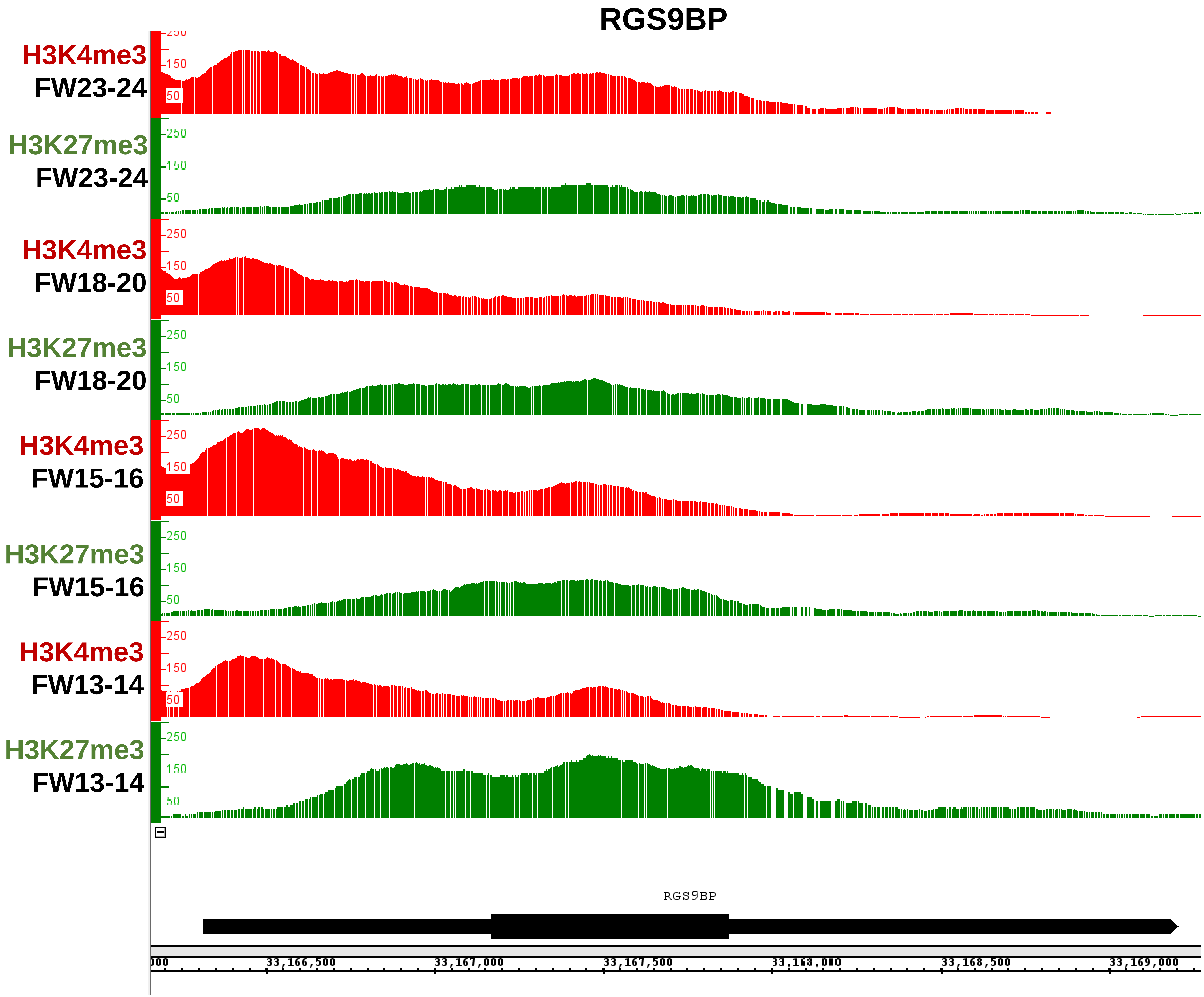

RGS9BP
H3K4me3
FW23-24
H3K27me3
FW23-24
H3K4me3
FW18-20
H3K27me3
FW18-20
H3K4me3
FW15-16
H3K27me3
FW15-16
H3K4me3
FW13-14
H3K27me3
FW13-14
RGS9BP
33,166,500
33,167,000
33,167,500
33,168,000
33,168,500
33,169,000

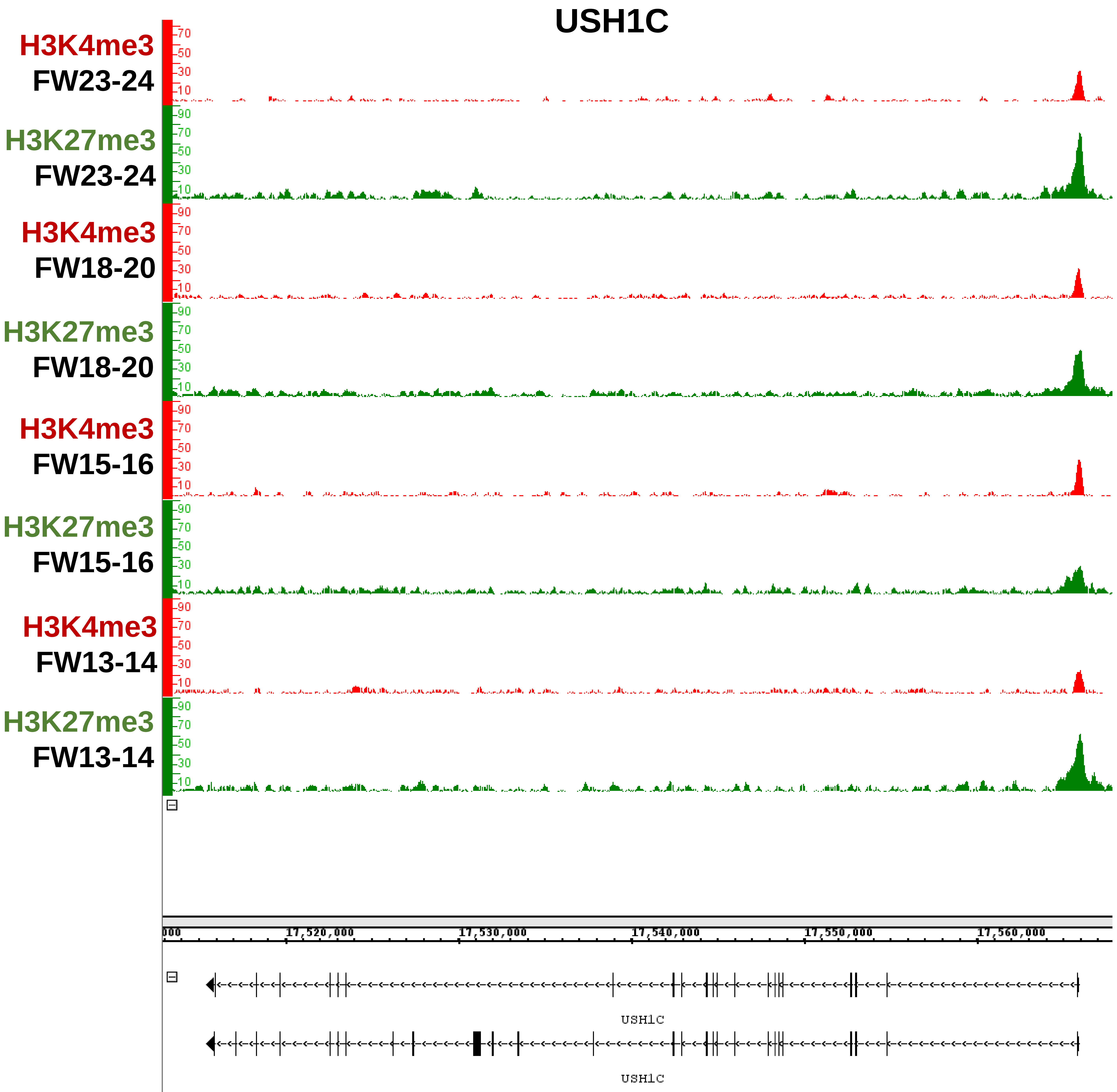
USH1C
H3K4me3
FW23-24
H3K27me3
FW23-24
H3K4me3
FW18-20
H3K27me3
FW18-20
H3K4me3
FW15-16
H3K27me3
FW15-16
H3K4me3
FW13-14
H3K27me3
FW13-14
17,520,000
17,530,000
17,540,000
17,550,000
17,560,000
USH1C
USH1C
