# Supplementary material for: The Potential Role of Epigenetic Mechanisms in the Development of Retinitis Pigmentosa and Related Photoreceptor Dystrophies
Source: Front Genet. 2022 Mar 11;13:827274. doi: 10.3389/fgene.2022.827274 (PMC8961674; doi:10.3389/fgene.2022.827274)
Supplement: Supplementary file 3 [file DataSheet4.PDF]

Title: The potential role of epigenetic mechanisms in the development of retinitis pigmentosa and related photoreceptor dystrophies

Galina Dvoriantchikova, Bascom Palmer Eye Institute, Department of Ophthalmology, University of Miami Miller School of Medicine, Miami, FL, 33136, USA

Karin Rose Lypka, Bascom Palmer Eye Institute, Department of Ophthalmology, University of Miami Miller School of Medicine, Miami, FL, 33136, USA

Dmitry Ivanov , Bascom Palmer Eye Institute, Department of Ophthalmology, University of Miami Miller School of Medicine, Miami, FL, 33136, USA; Department of Microbiology and Immunology, University of Miami Miller School of Medicine, Miami, FL, 33136, USA; divanov@med.miami.edu.

Supplementary Data S10: ChIP-seq data in Integrated Genome Browser to visually verify the chromatin state in promoters of identified genes in Supplementary Data S9, whose states was not clear

Plk5

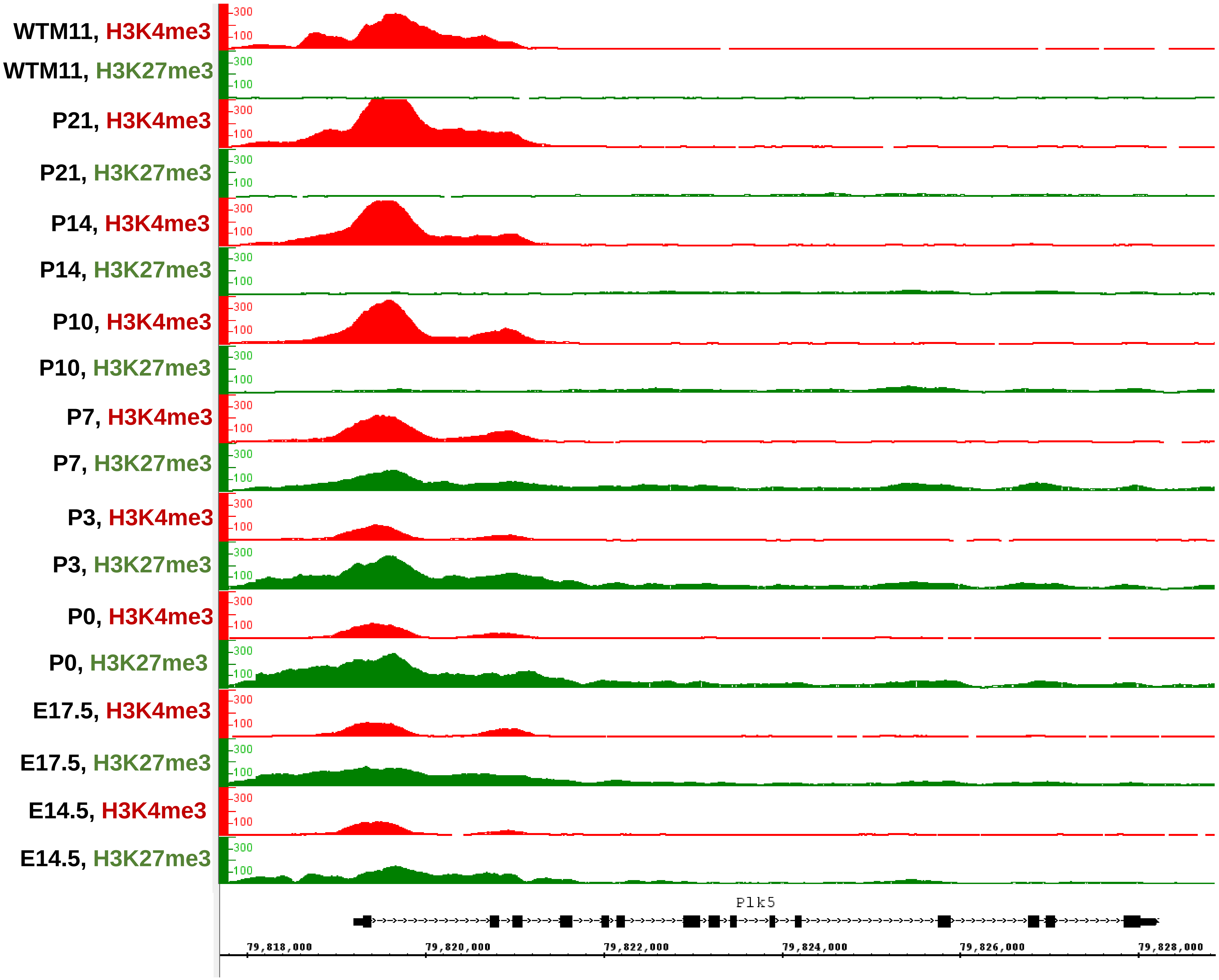
WTM11, H3K4me3
WTM11, H3K27me3
P21, H3K4me3
P21, H3K27me3
P14, H3K4me3
P14, H3K27me3
P10, H3K4me3
P10, H3K27me3
P7, H3K4me3
P7, H3K27me3
P3, H3K4me3
P3, H3K27me3
P0, H3K4me3
P0, H3K27me3
E17.5, H3K4me3
E17.5, H3K27me3
E14.5, H3K4me3
E14.5, H3K27me3
300
100
Plk5
79,818,000
79,820,000
79,822,000
79,824,000
79,826,000
79,828,000

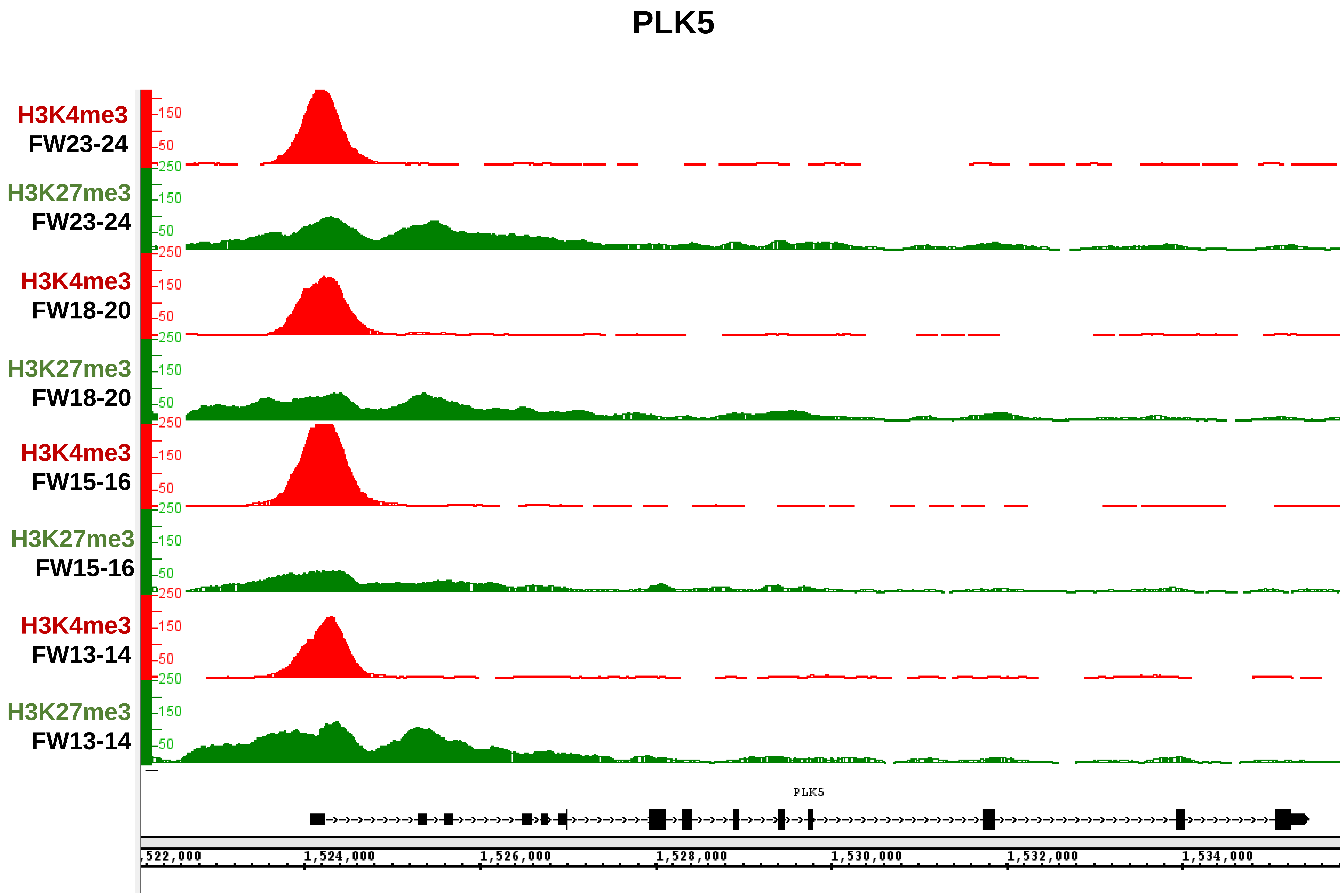
PLK5
H3K4me3 FW23-24
H3K27me3 FW23-24
H3K4me3 FW18-20
H3K27me3 FW18-20
H3K4me3 FW15-16
H3K27me3 FW15-16
H3K4me3 FW13-14
H3K27me3 FW13-14
150
50
250
PLK5
1,522,000
1,524,000
1,526,000
1,528,000
1,530,000
1,532,000
1,534,000

Ppm1n

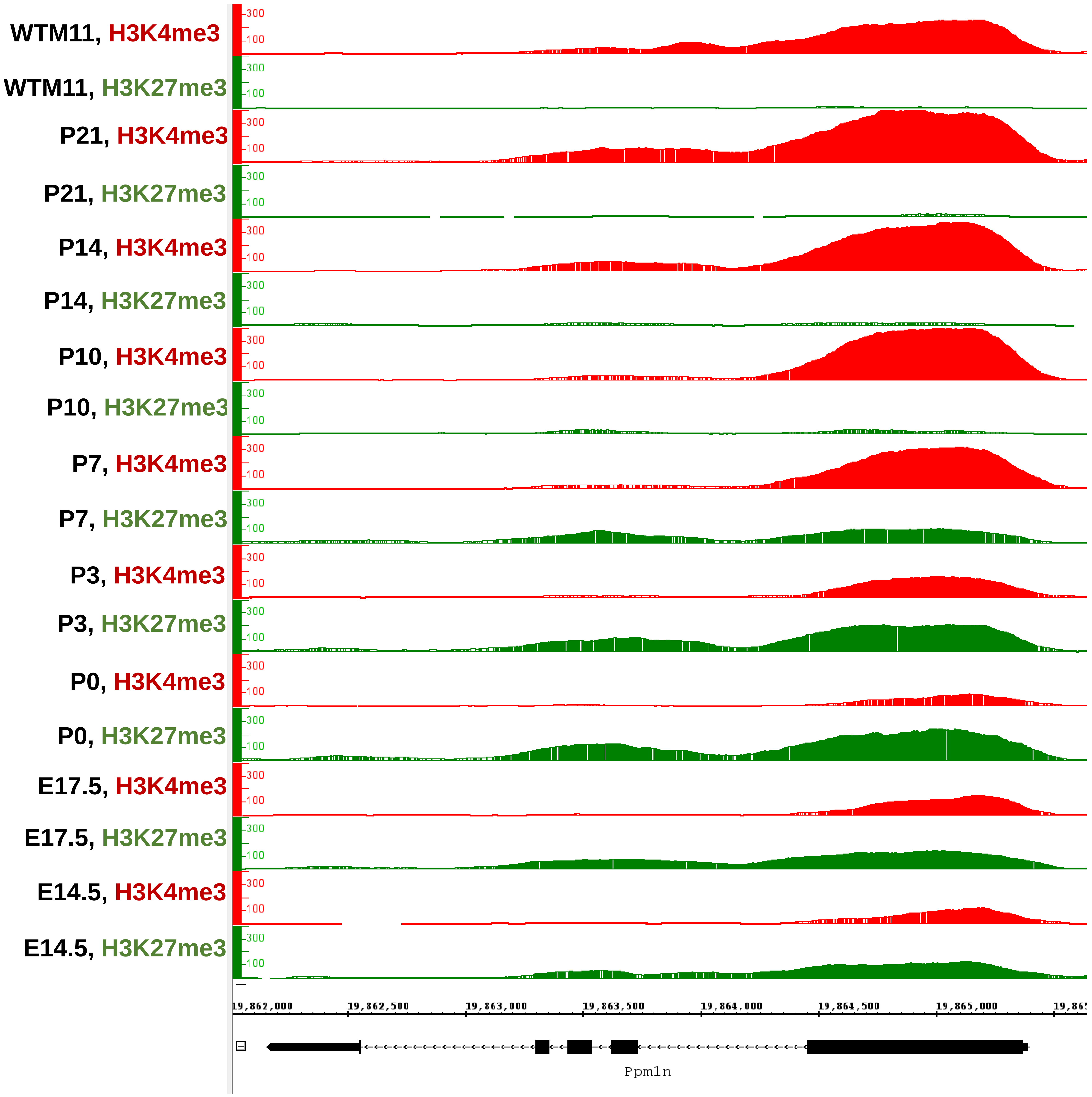
WTM11, H3K4me3
WTM11, H3K27me3
P21, H3K4me3
P21, H3K27me3
P14, H3K4me3
P14, H3K27me3
P10, H3K4me3
P10, H3K27me3
P7, H3K4me3
P7, H3K27me3
P3, H3K4me3
P3, H3K27me3
P0, H3K4me3
P0, H3K27me3
E17.5, H3K4me3
E17.5, H3K27me3
E14.5, H3K4me3
E14.5, H3K27me3
300
100
19,862,000
19,862,500
19,863,000
19,863,500
19,864,000
19,864,500
19,865,000
Ppm1n

PPM1N

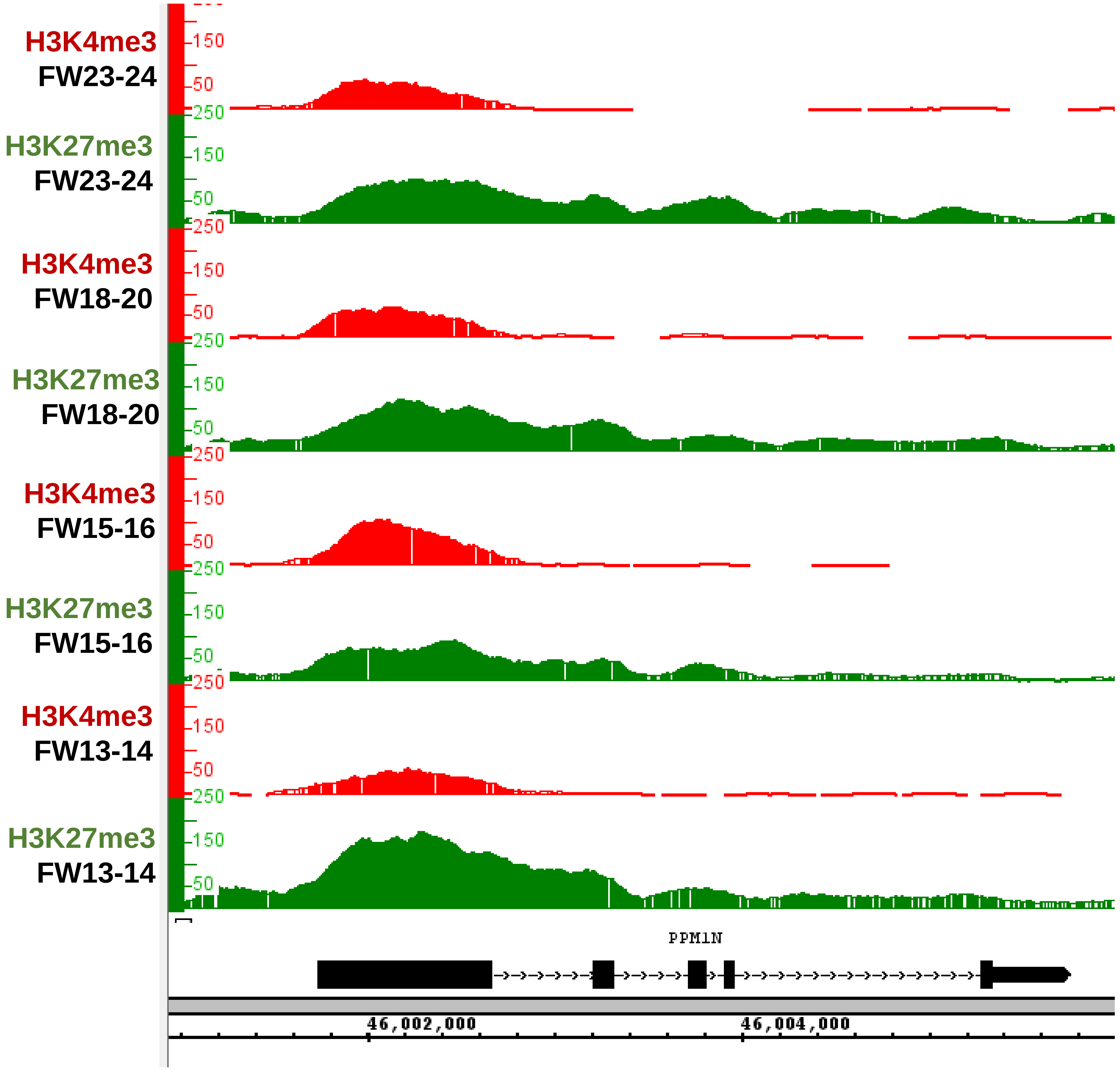
H3K4me3 FW23-24
H3K27me3 FW23-24
H3K4me3 FW18-20
H3K27me3 FW18-20
H3K4me3 FW15-16
H3K27me3 FW15-16
H3K4me3 FW13-14
H3K27me3 FW13-14
250
150
50
PPM1N
46,002,000
46,004,000

H2-K2

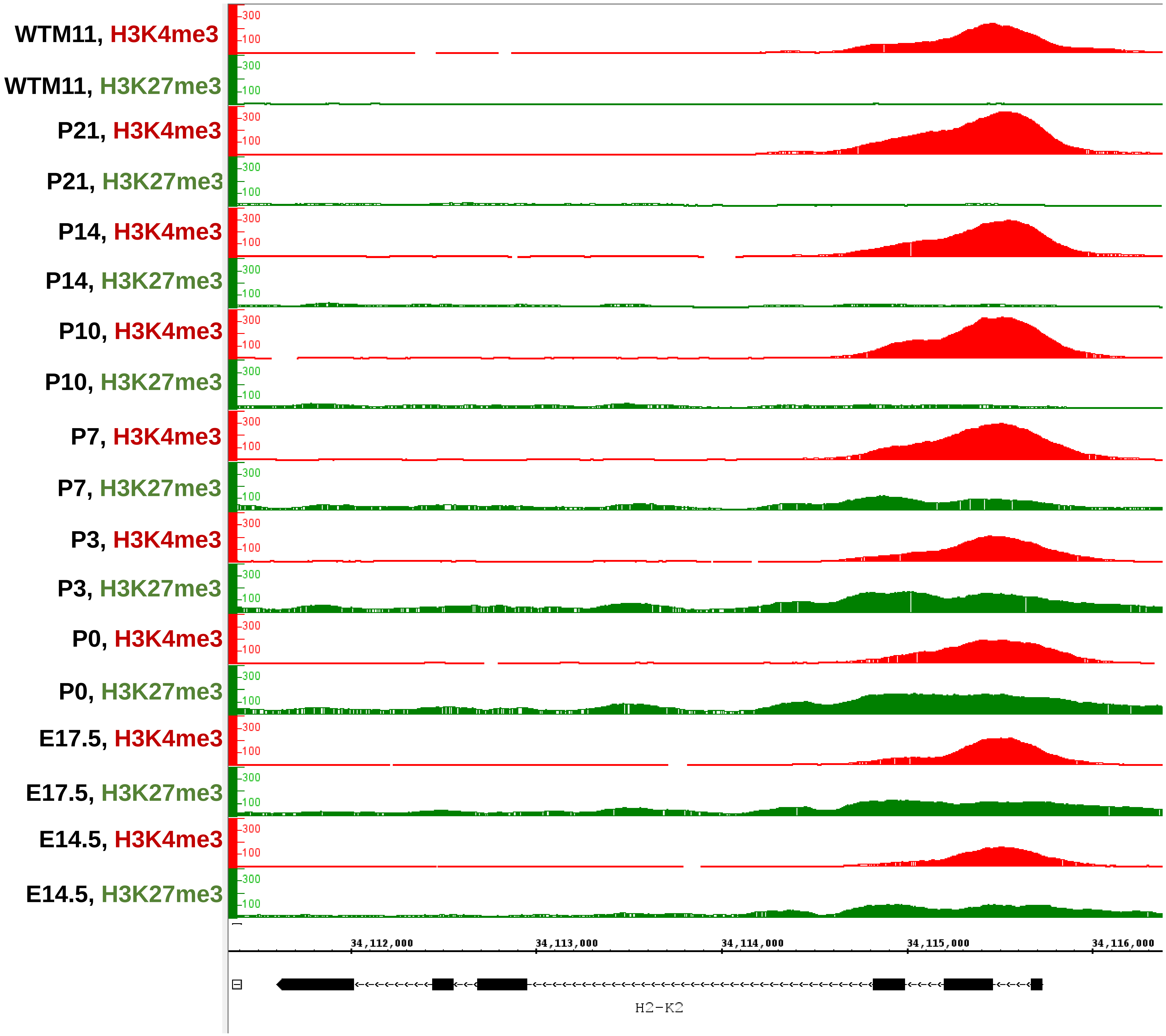
WTM11, H3K4me3
WTM11, H3K27me3
P21, H3K4me3
P21, H3K27me3
P14, H3K4me3
P14, H3K27me3
P10, H3K4me3
P10, H3K27me3
P7, H3K4me3
P7, H3K27me3
P3, H3K4me3
P3, H3K27me3
P0, H3K4me3
P0, H3K27me3
E17.5, H3K4me3
E17.5, H3K27me3
E14.5, H3K4me3
E14.5, H3K27me3
300
100
34,112,000
34,113,000
34,114,000
34,115,000
34,116,000
H2-K2

Pde8a

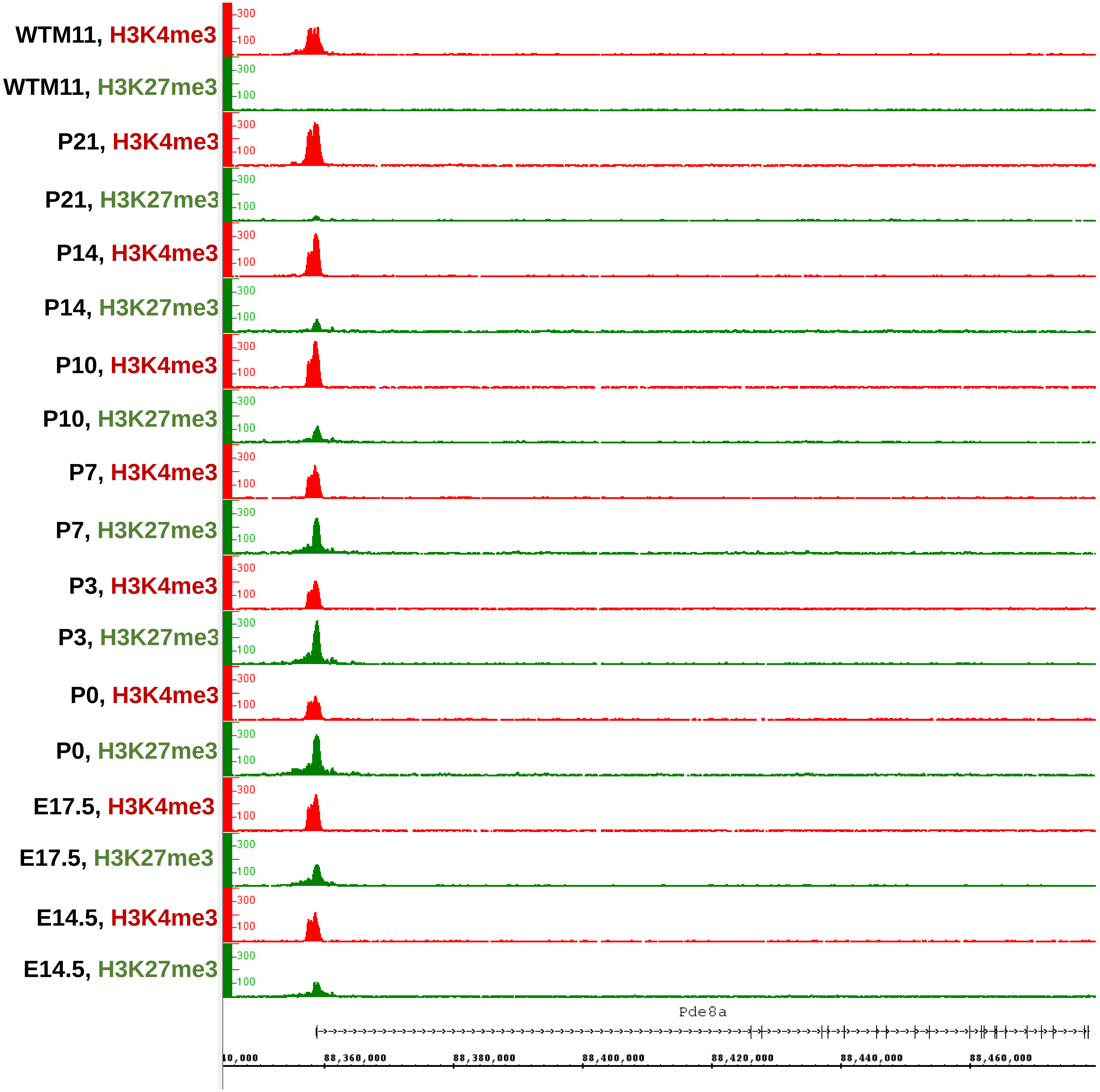
WTM11, H3K4me3
WTM11, H3K27me3
P21, H3K4me3
P21, H3K27me3
P14, H3K4me3
P14, H3K27me3
P10, H3K4me3
P10, H3K27me3
P7, H3K4me3
P7, H3K27me3
P3, H3K4me3
P3, H3K27me3
P0, H3K4me3
P0, H3K27me3
E17.5, H3K4me3
E17.5, H3K27me3
E14.5, H3K4me3
E14.5, H3K27me3
300
100
Pde8a
88,360,000
88,380,000
88,400,000
88,420,000
88,440,000
88,460,000

PDE8A

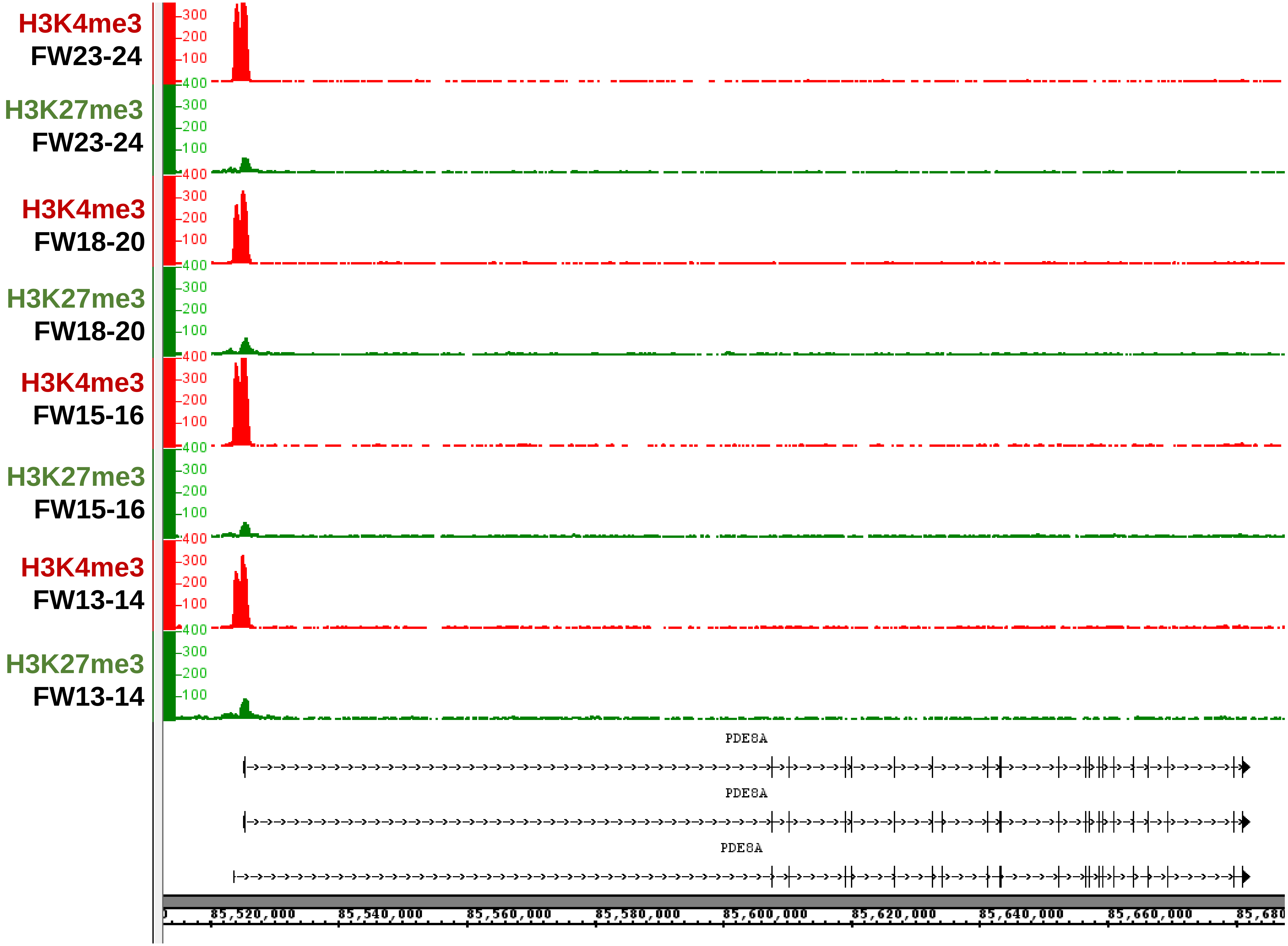
H3K4me3
FW23-24
H3K27me3
FW23-24
H3K4me3
FW18-20
H3K27me3
FW18-20
H3K4me3
FW15-16
H3K27me3
FW15-16
H3K4me3
FW13-14
H3K27me3
FW13-14
300
200
100
400
PDE8A
PDE8A
PDE8A
85,520,000
85,540,000
85,560,000
85,580,000
85,600,000
85,620,000
85,640,000
85,660,000
