# Supplementary material for: The Potential Role of Epigenetic Mechanisms in the Development of Retinitis Pigmentosa and Related Photoreceptor Dystrophies
Source: Front Genet. 2022 Mar 11;13:827274. doi: 10.3389/fgene.2022.827274 (PMC8961674; doi:10.3389/fgene.2022.827274)
Supplement: Supplementary file 5 [file DataSheet3.PDF]

Title: The potential role of epigenetic mechanisms in the development of retinitis pigmentosa and related photoreceptor dystrophies

Galina Dvoriantchikova, Bascom Palmer Eye Institute, Department of Ophthalmology, University of Miami Miller School of Medicine, Miami, FL, 33136, USA

Karin Rose Lypka, Bascom Palmer Eye Institute, Department of Ophthalmology, University of Miami Miller School of Medicine, Miami, FL, 33136, USA

Dmitry Ivanov , Bascom Palmer Eye Institute, Department of Ophthalmology, University of Miami Miller School of Medicine, Miami, FL, 33136, USA; Department of Microbiology and Immunology, University of Miami Miller School of Medicine, Miami, FL, 33136, USA; divanov@med.miami.edu.

Supplementary Data S5: ChIP-seq data in Integrated Genome Browser to visually verify the chromatin state in promoters of studied mouse genes, whose state was not clear

Cdhr1

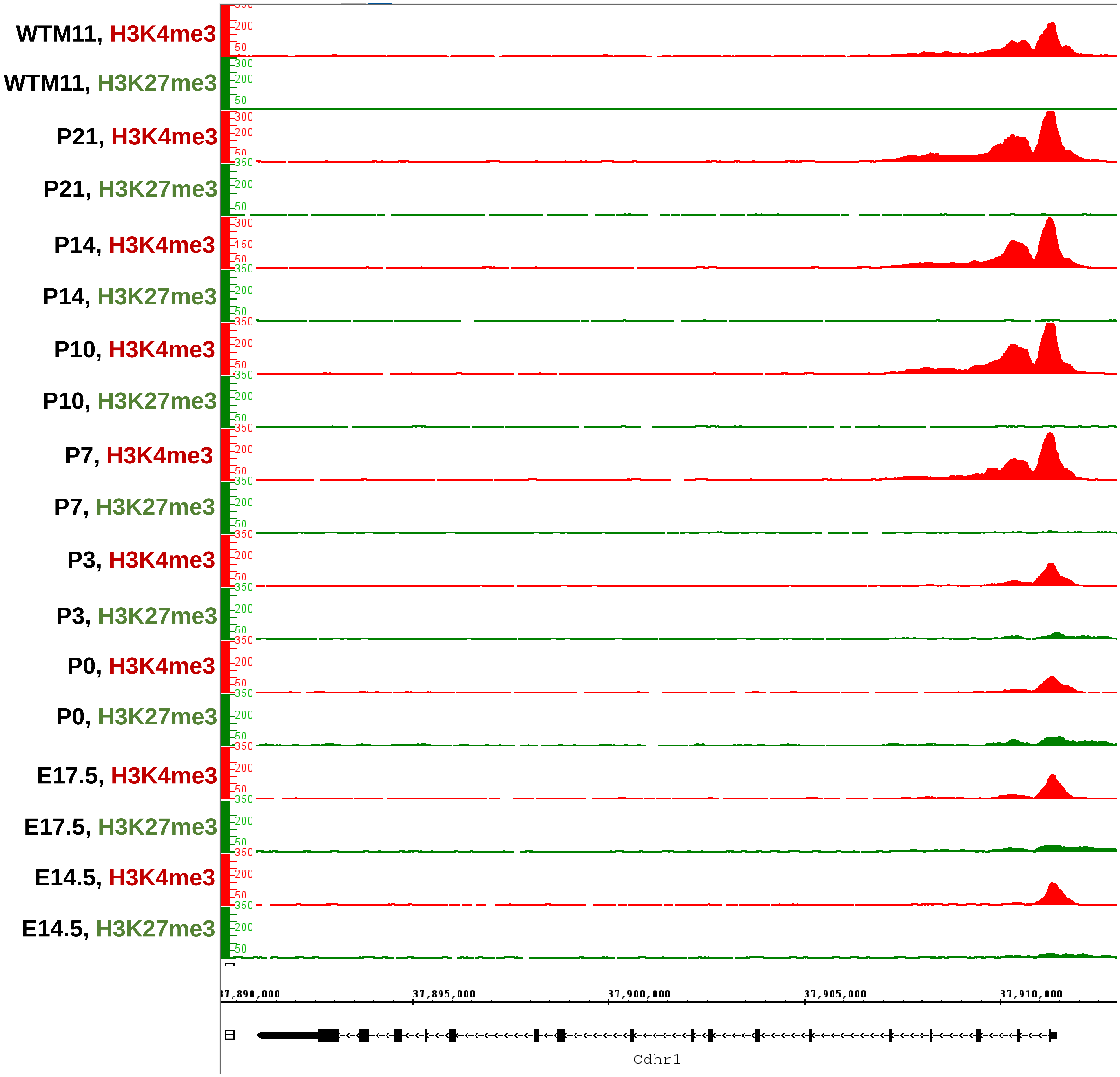

WTM11, H3K4me3
WTM11, H3K27me3
P21, H3K4me3
P21, H3K27me3
P14, H3K4me3
P14, H3K27me3
P10, H3K4me3
P10, H3K27me3
P7, H3K4me3
P7, H3K27me3
P3, H3K4me3
P3, H3K27me3
P0, H3K4me3
P0, H3K27me3
E17.5, H3K4me3
E17.5, H3K27me3
E14.5, H3K4me3
E14.5, H3K27me3
37,895,000
37,900,000
37,905,000
37,910,000
Cdhr1

Cerkl

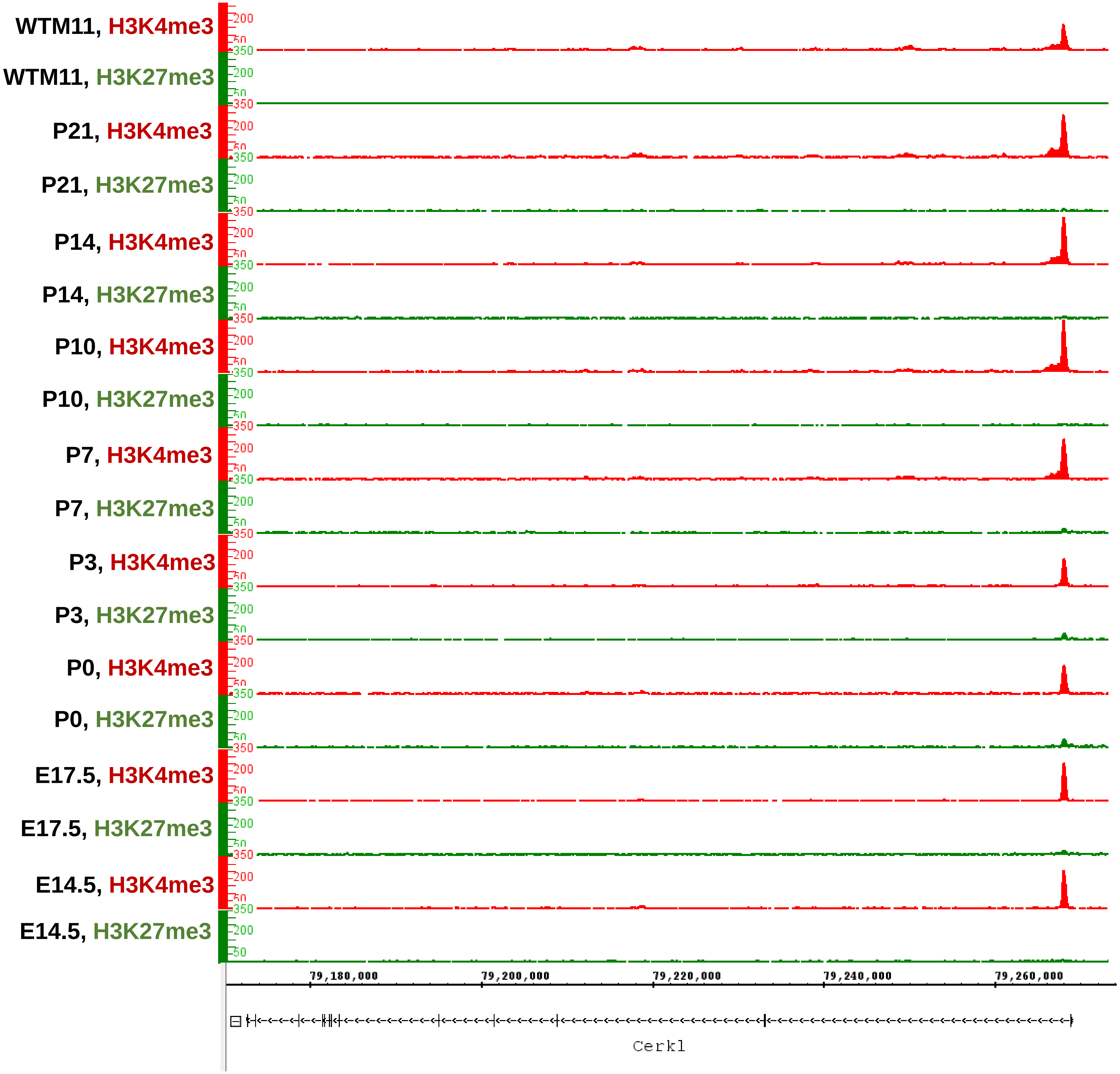

WTM11, H3K4me3
WTM11, H3K27me3
P21, H3K4me3
P21, H3K27me3
P14, H3K4me3
P14, H3K27me3
P10, H3K4me3
P10, H3K27me3
P7, H3K4me3
P7, H3K27me3
P3, H3K4me3
P3, H3K27me3
P0, H3K4me3
P0, H3K27me3
E17.5, H3K4me3
E17.5, H3K27me3
E14.5, H3K4me3
E14.5, H3K27me3
200
50
350
79,180,000
79,200,000
79,220,000
79,240,000
79,260,000
Cerkl

Gdf6

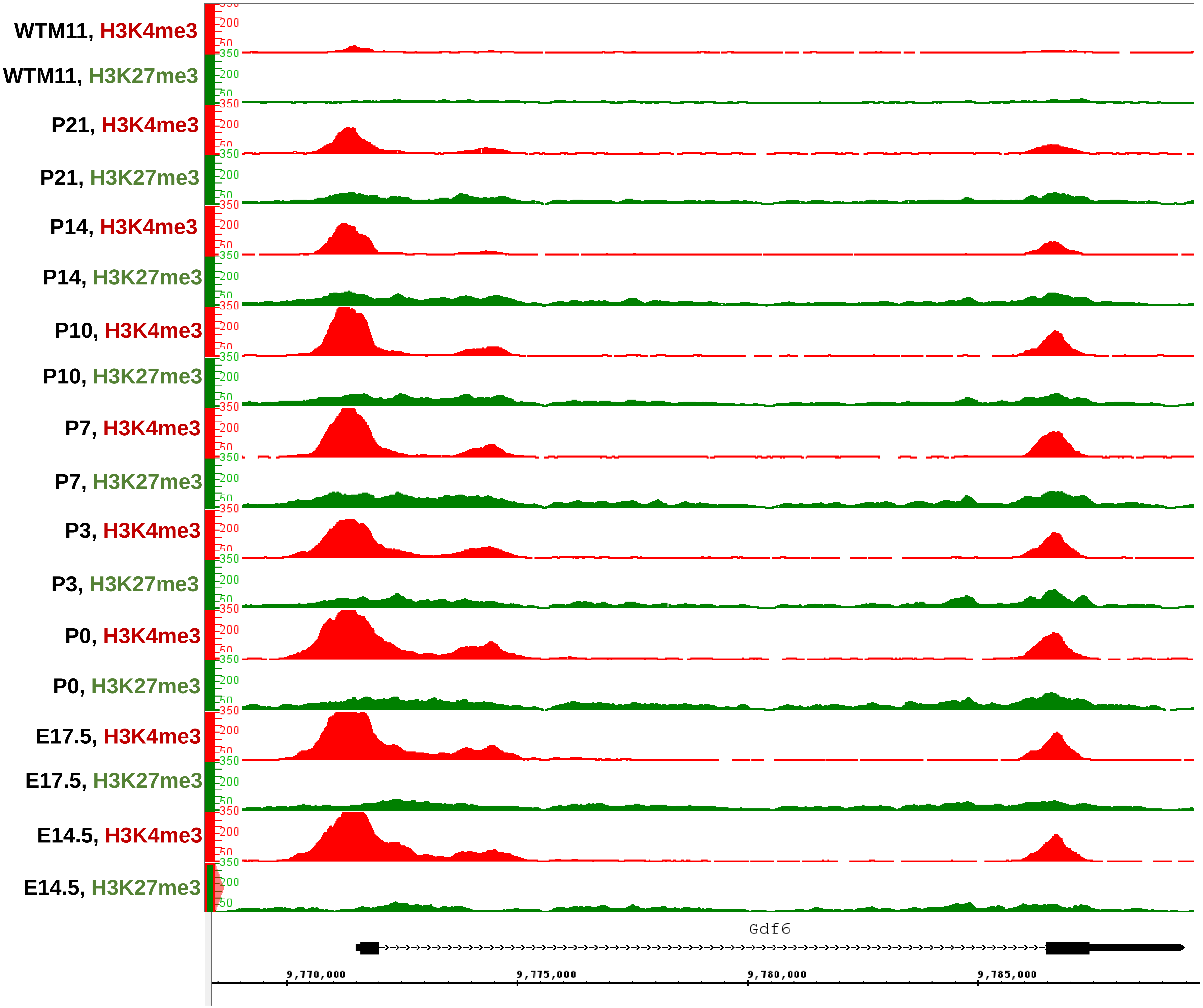

WTM11, H3K4me3
WTM11, H3K27me3
P21, H3K4me3
P21, H3K27me3
P14, H3K4me3
P14, H3K27me3
P10, H3K4me3
P10, H3K27me3
P7, H3K4me3
P7, H3K27me3
P3, H3K4me3
P3, H3K27me3
P0, H3K4me3
P0, H3K27me3
E17.5, H3K4me3
E17.5, H3K27me3
E14.5, H3K4me3
E14.5, H3K27me3
200
50
350
Gdf6
9,770,000
9,775,000
9,780,000
9,785,000

Guca1b

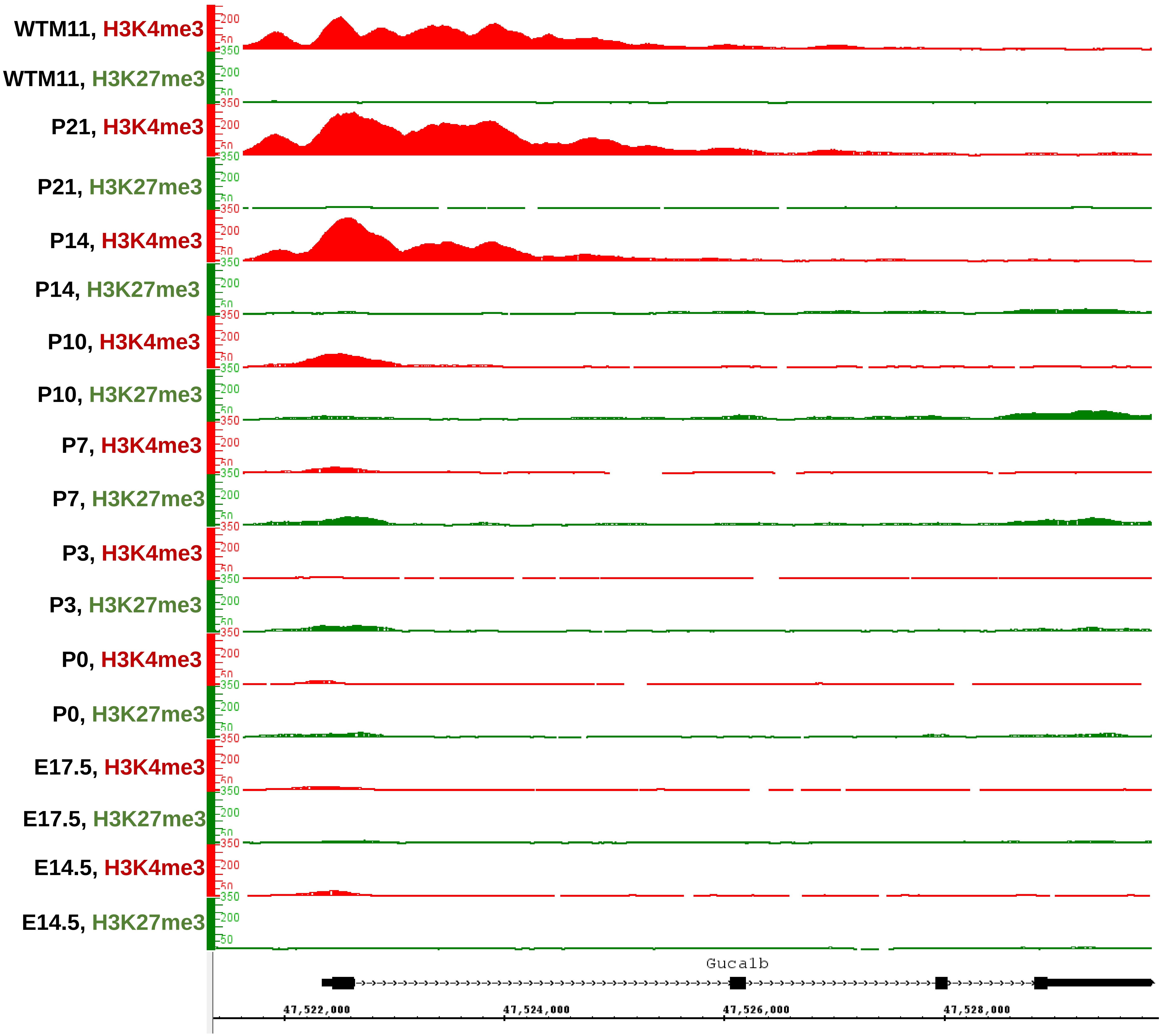
WTM11, H3K4me3
WTM11, H3K27me3
P21, H3K4me3
P21, H3K27me3
P14, H3K4me3
P14, H3K27me3
P10, H3K4me3
P10, H3K27me3
P7, H3K4me3
P7, H3K27me3
P3, H3K4me3
P3, H3K27me3
P0, H3K4me3
P0, H3K27me3
E17.5, H3K4me3
E17.5, H3K27me3
E14.5, H3K4me3
E14.5, H3K27me3
350
200
50
Guca1b
47,522,000
47,524,000
47,526,000
47,528,000

Hmcn1

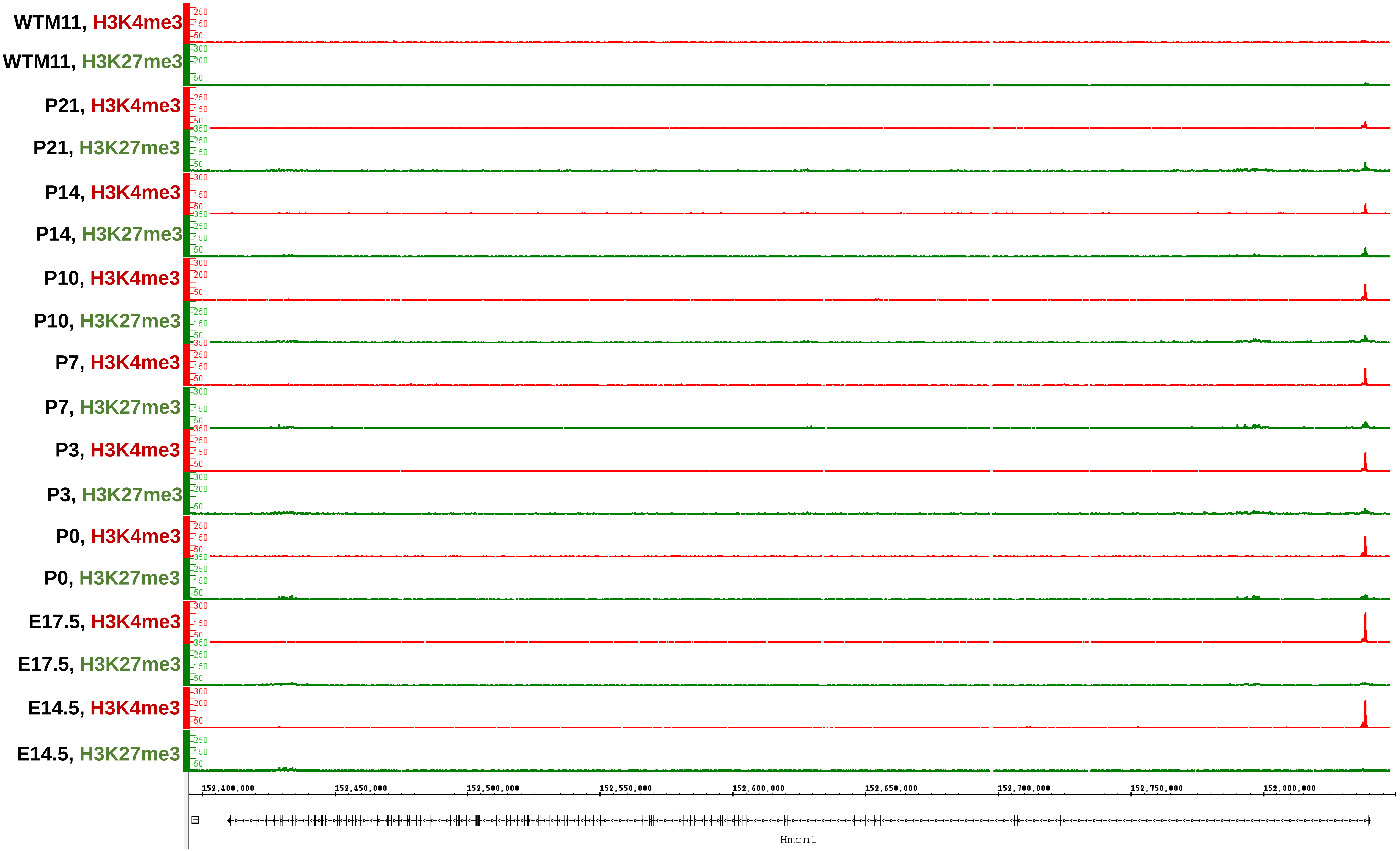

WTM11, H3K4me3
WTM11, H3K27me3
P21, H3K4me3
P21, H3K27me3
P14, H3K4me3
P14, H3K27me3
P10, H3K4me3
P10, H3K27me3
P7, H3K4me3
P7, H3K27me3
P3, H3K4me3
P3, H3K27me3
P0, H3K4me3
P0, H3K27me3
E17.5, H3K4me3
E17.5, H3K27me3
E14.5, H3K4me3
E14.5, H3K27me3
152,400,000
152,450,000
152,500,000
152,550,000
152,600,000
152,650,000
152,700,000
152,750,000
152,800,000
Hmcn1

Mak

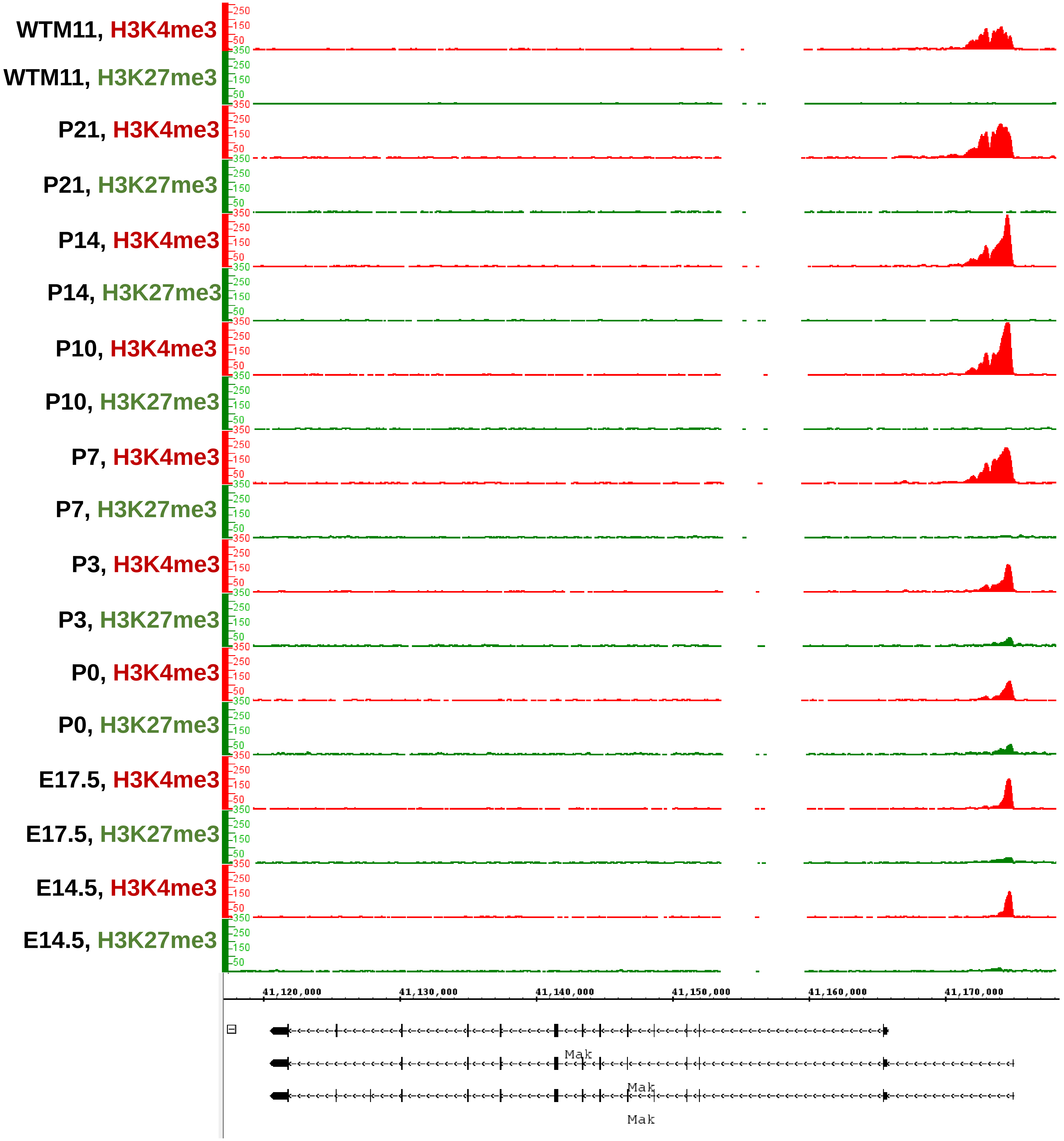
WTM11, H3K4me3
WTM11, H3K27me3
P21, H3K4me3
P21, H3K27me3
P14, H3K4me3
P14, H3K27me3
P10, H3K4me3
P10, H3K27me3
P7, H3K4me3
P7, H3K27me3
P3, H3K4me3
P3, H3K27me3
P0, H3K4me3
P0, H3K27me3
E17.5, H3K4me3
E17.5, H3K27me3
E14.5, H3K4me3
E14.5, H3K27me3
350
250
150
50
41,120,000
41,130,000
41,140,000
41,150,000
41,160,000
41,170,000
Mak
Mak
Mak

Mertk

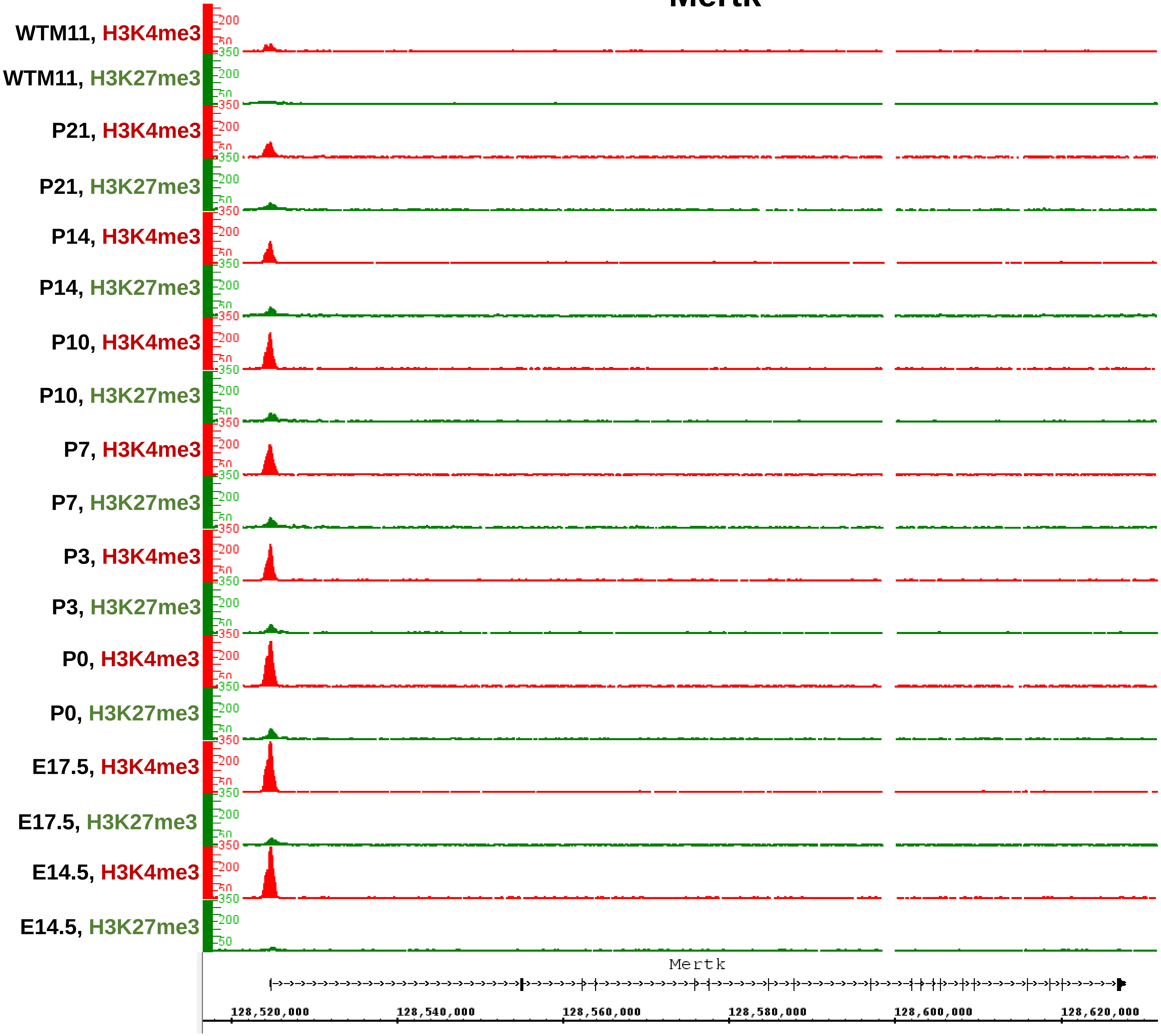

WTM11, H3K4me3
WTM11, H3K27me3
P21, H3K4me3
P21, H3K27me3
P14, H3K4me3
P14, H3K27me3
P10, H3K4me3
P10, H3K27me3
P7, H3K4me3
P7, H3K27me3
P3, H3K4me3
P3, H3K27me3
P0, H3K4me3
P0, H3K27me3
E17.5, H3K4me3
E17.5, H3K27me3
E14.5, H3K4me3
E14.5, H3K27me3
200
50
350
Mertk
128,520,000
128,540,000
128,560,000
128,580,000
128,600,000
128,620,000

Neurod1

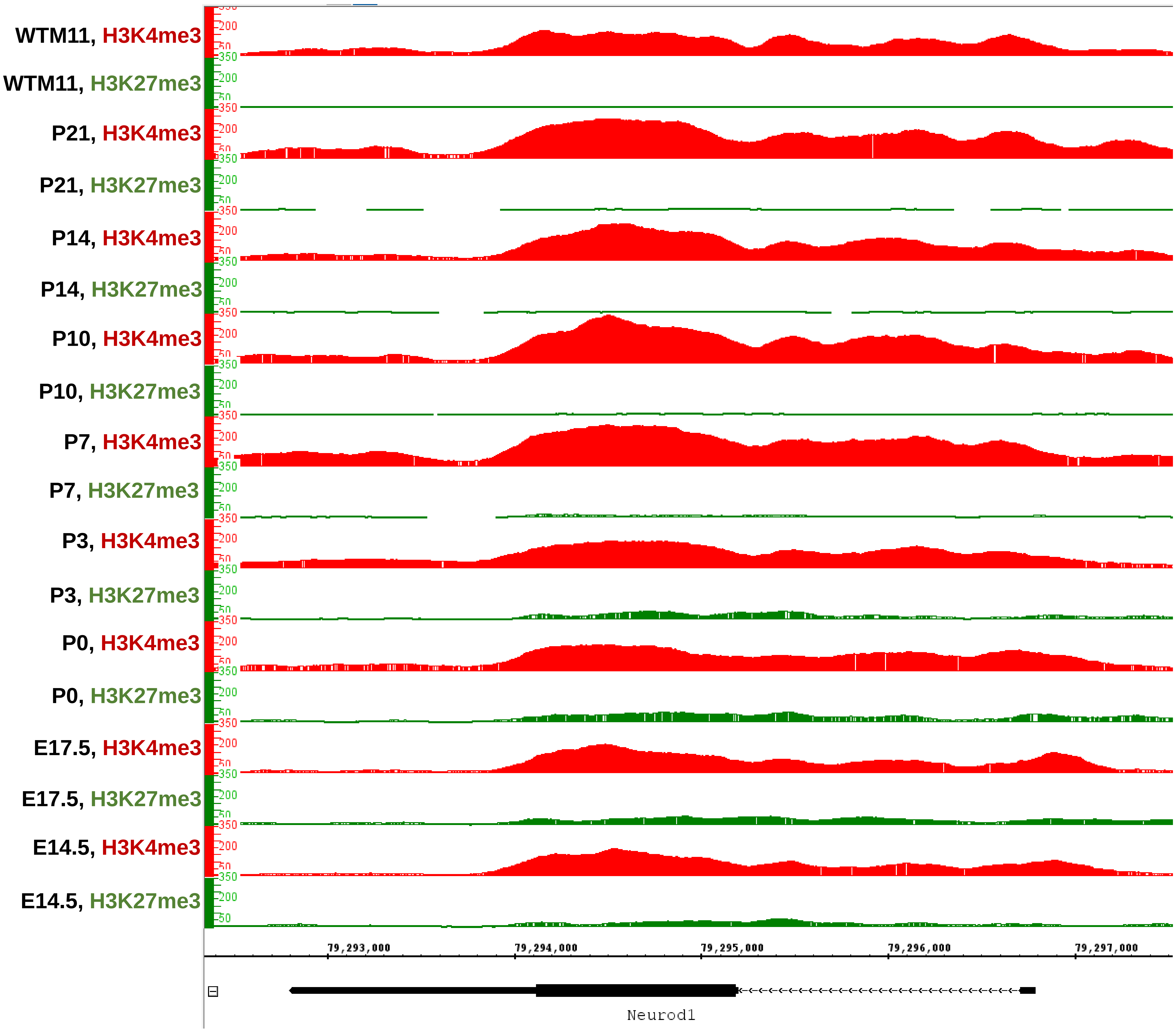

WTM11, H3K4me3
WTM11, H3K27me3
P21, H3K4me3
P21, H3K27me3
P14, H3K4me3
P14, H3K27me3
P10, H3K4me3
P10, H3K27me3
P7, H3K4me3
P7, H3K27me3
P3, H3K4me3
P3, H3K27me3
P0, H3K4me3
P0, H3K27me3
E17.5, H3K4me3
E17.5, H3K27me3
E14.5, H3K4me3
E14.5, H3K27me3
350
200
50
79,293,000
79,294,000
79,295,000
79,296,000
79,297,000
Neurod1

Pitpnm3

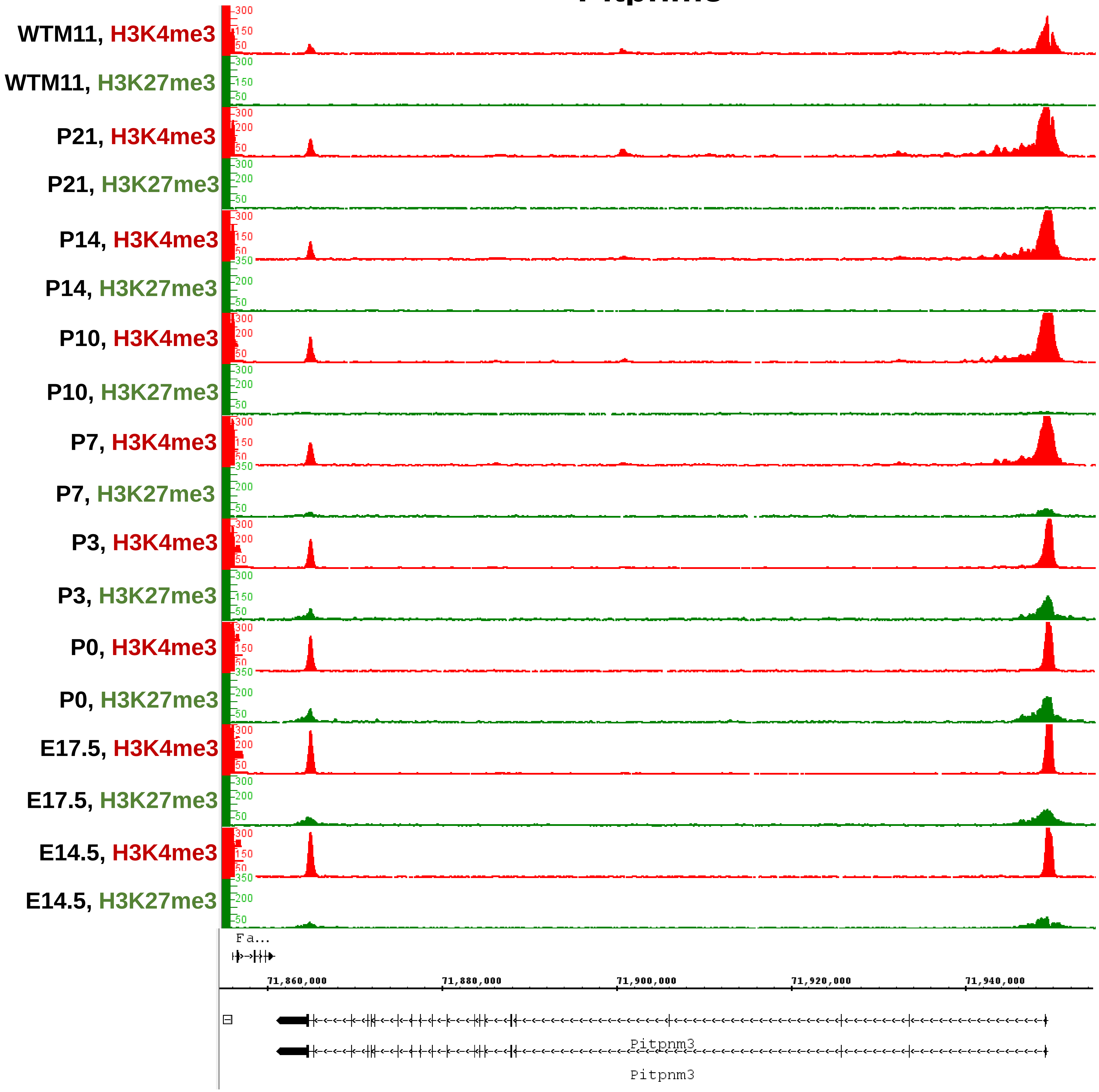
WTM11, H3K4me3
WTM11, H3K27me3
P21, H3K4me3
P21, H3K27me3
P14, H3K4me3
P14, H3K27me3
P10, H3K4me3
P10, H3K27me3
P7, H3K4me3
P7, H3K27me3
P3, H3K4me3
P3, H3K27me3
P0, H3K4me3
P0, H3K27me3
E17.5, H3K4me3
E17.5, H3K27me3
E14.5, H3K4me3
E14.5, H3K27me3
71,860,000
71,880,000
71,900,000
71,920,000
71,940,000
Pitpnm3
Pitpnm3

Rdh12

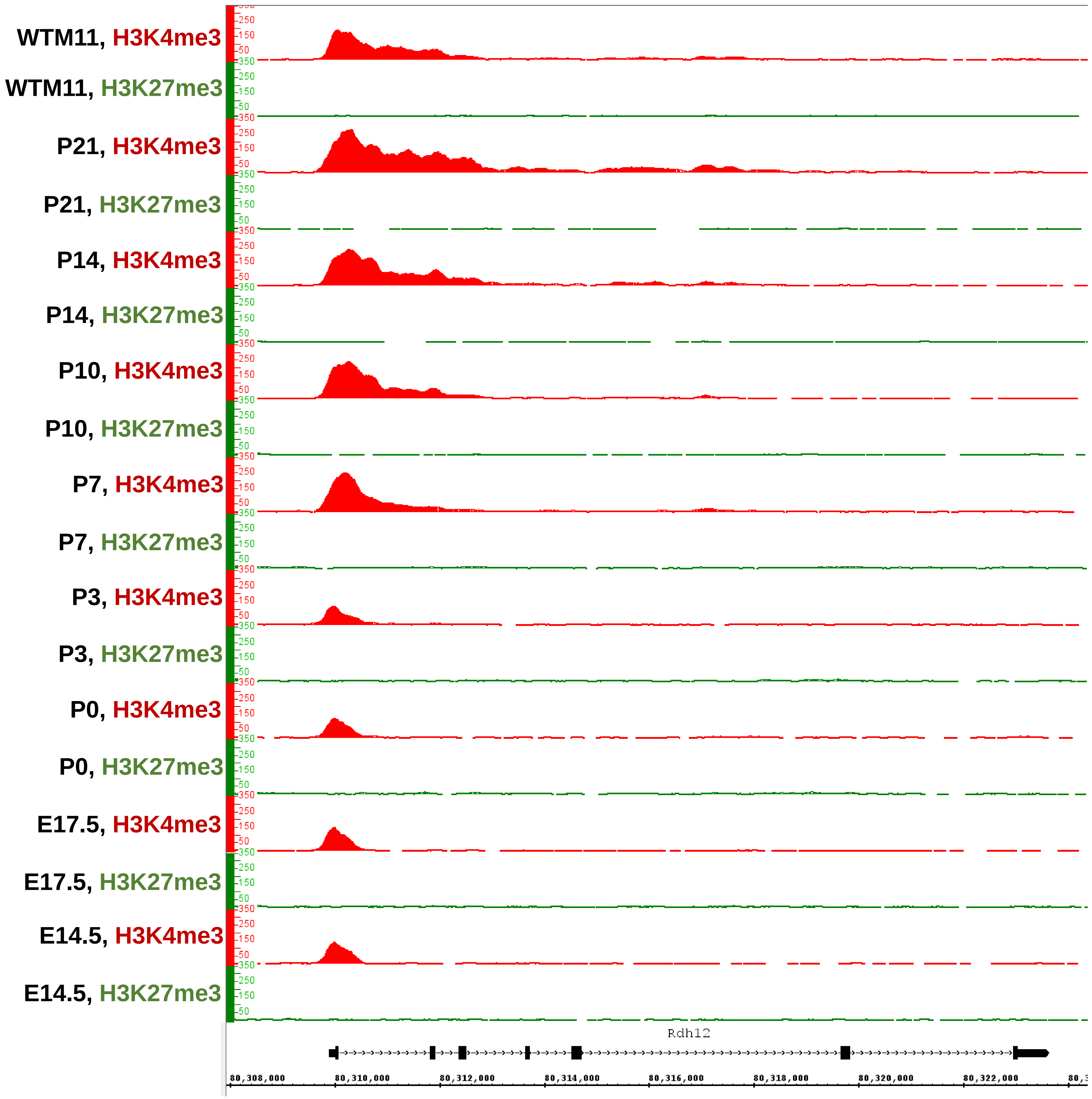
WTM11, H3K4me3
WTM11, H3K27me3
P21, H3K4me3
P21, H3K27me3
P14, H3K4me3
P14, H3K27me3
P10, H3K4me3
P10, H3K27me3
P7, H3K4me3
P7, H3K27me3
P3, H3K4me3
P3, H3K27me3
P0, H3K4me3
P0, H3K27me3
E17.5, H3K4me3
E17.5, H3K27me3
E14.5, H3K4me3
E14.5, H3K27me3
Rdh12
80,308,000
80,310,000
80,312,000
80,314,000
80,316,000
80,318,000
80,320,000
80,322,000

Reep6

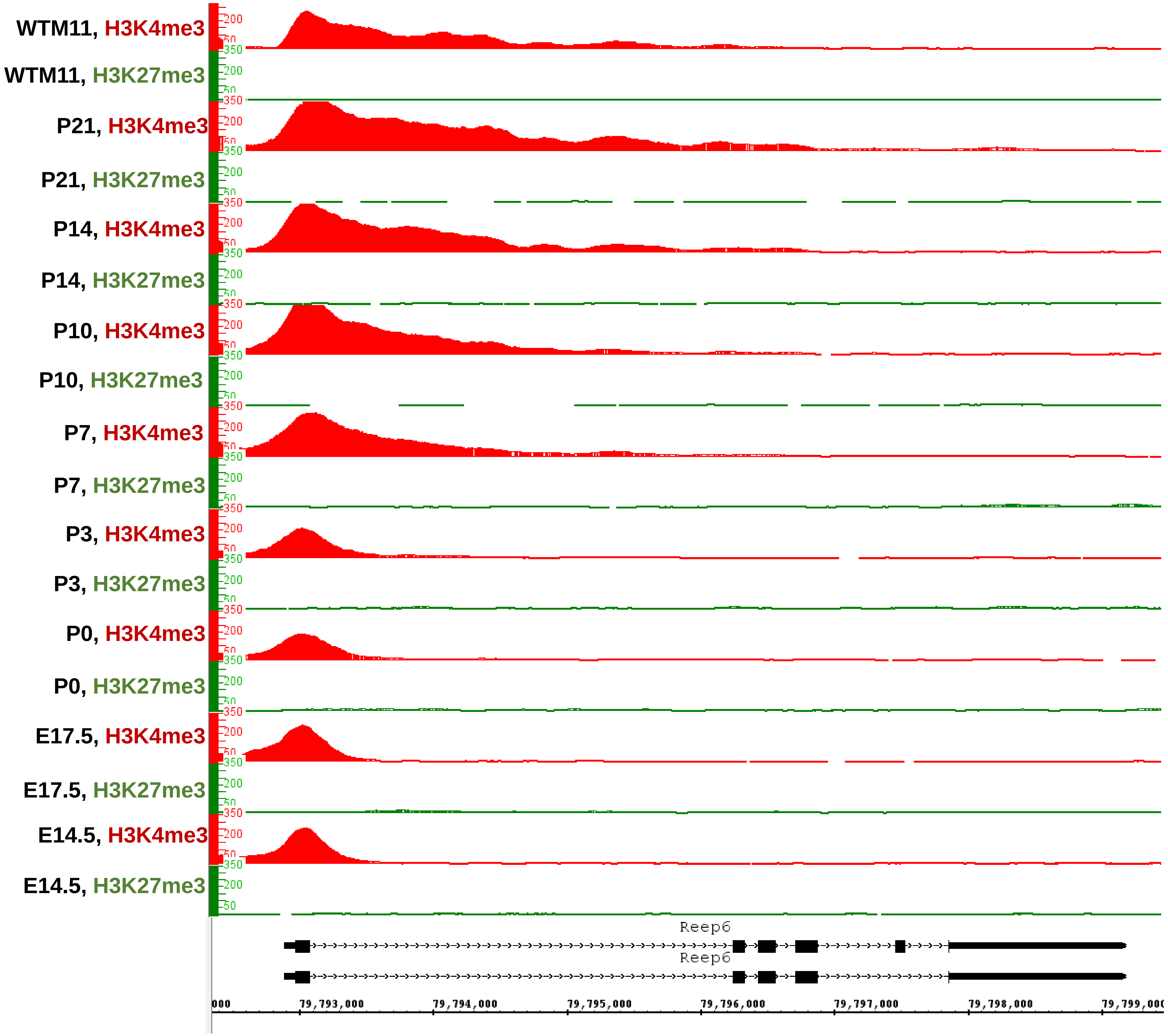
WTM11, H3K4me3
WTM11, H3K27me3
P21, H3K4me3
P21, H3K27me3
P14, H3K4me3
P14, H3K27me3
P10, H3K4me3
P10, H3K27me3
P7, H3K4me3
P7, H3K27me3
P3, H3K4me3
P3, H3K27me3
P0, H3K4me3
P0, H3K27me3
E17.5, H3K4me3
E17.5, H3K27me3
E14.5, H3K4me3
E14.5, H3K27me3
350
200
50
Reep6
Reep6
79,793,000
79,794,000
79,795,000
79,796,000
79,797,000
79,798,000

Slc24a1

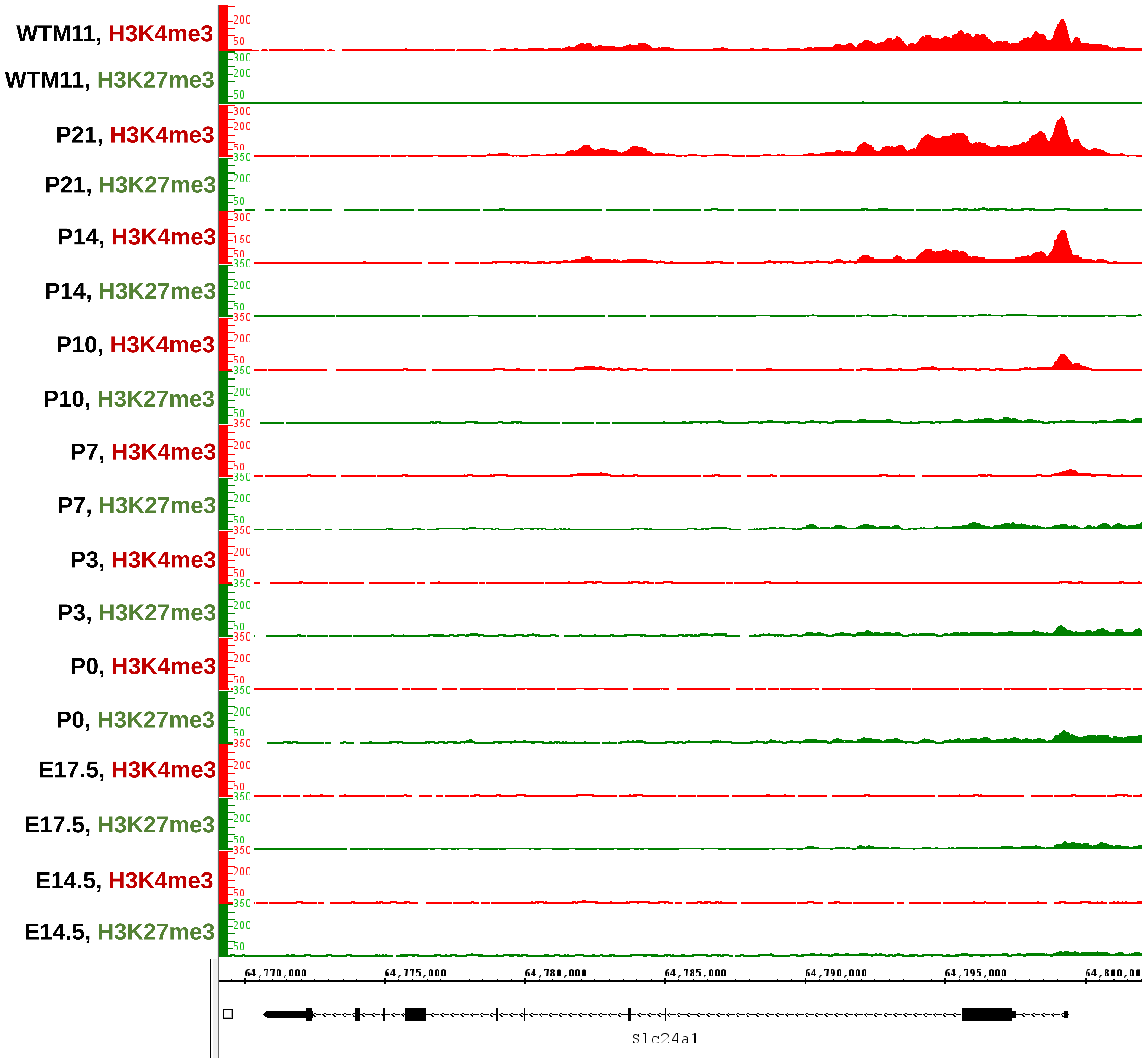

WTM11, H3K4me3
WTM11, H3K27me3
P21, H3K4me3
P21, H3K27me3
P14, H3K4me3
P14, H3K27me3
P10, H3K4me3
P10, H3K27me3
P7, H3K4me3
P7, H3K27me3
P3, H3K4me3
P3, H3K27me3
P0, H3K4me3
P0, H3K27me3
E17.5, H3K4me3
E17.5, H3K27me3
E14.5, H3K4me3
E14.5, H3K27me3
64,770,000
64,775,000
64,780,000
64,785,000
64,790,000
64,795,000
64,800,00
Slc24a1

Atp8a2

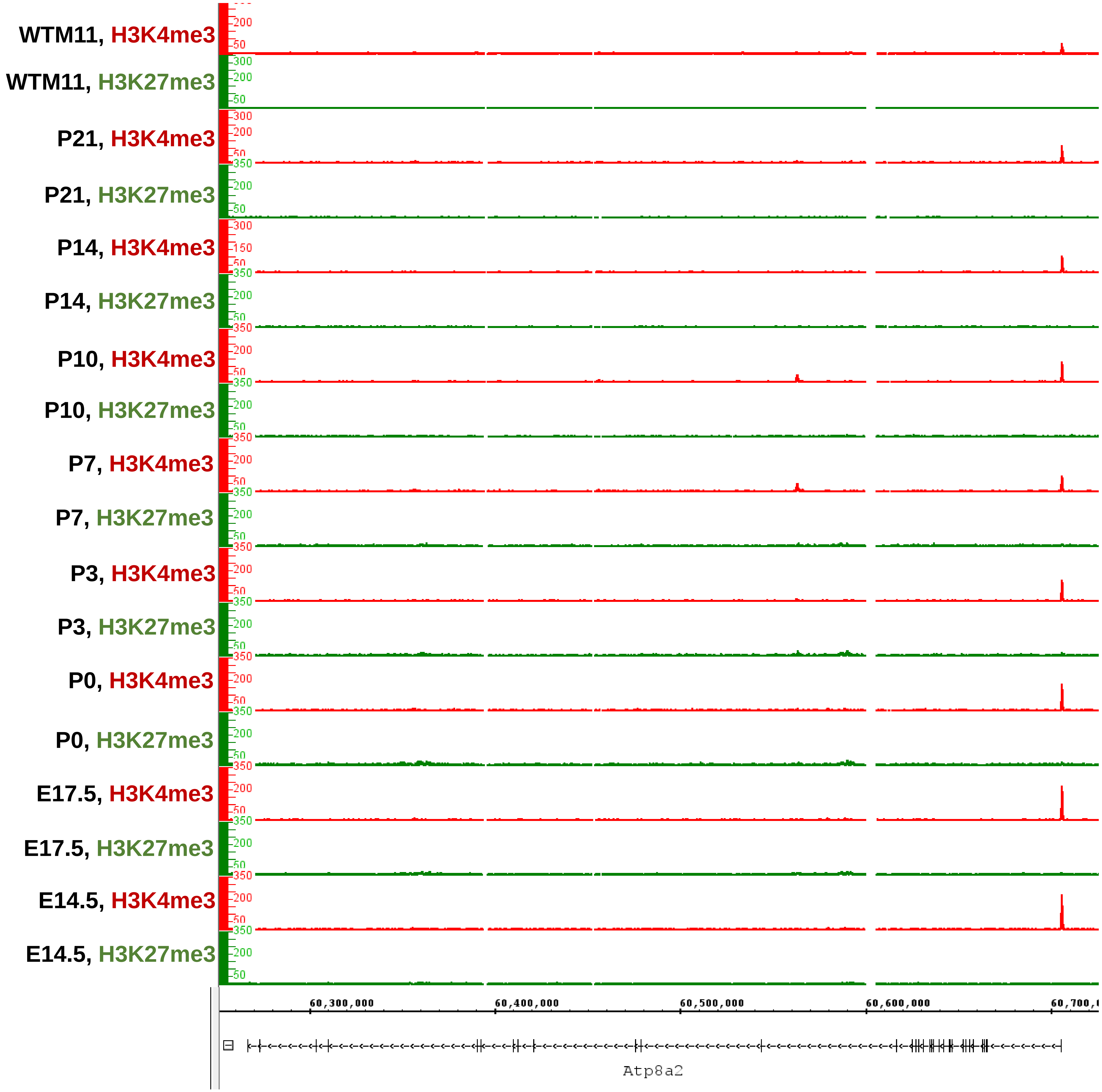
WTM11, H3K4me3
WTM11, H3K27me3
P21, H3K4me3
P21, H3K27me3
P14, H3K4me3
P14, H3K27me3
P10, H3K4me3
P10, H3K27me3
P7, H3K4me3
P7, H3K27me3
P3, H3K4me3
P3, H3K27me3
P0, H3K4me3
P0, H3K27me3
E17.5, H3K4me3
E17.5, H3K27me3
E14.5, H3K4me3
E14.5, H3K27me3
60,300,000
60,400,000
60,500,000
60,600,000
Atp8a2

Cacnb2

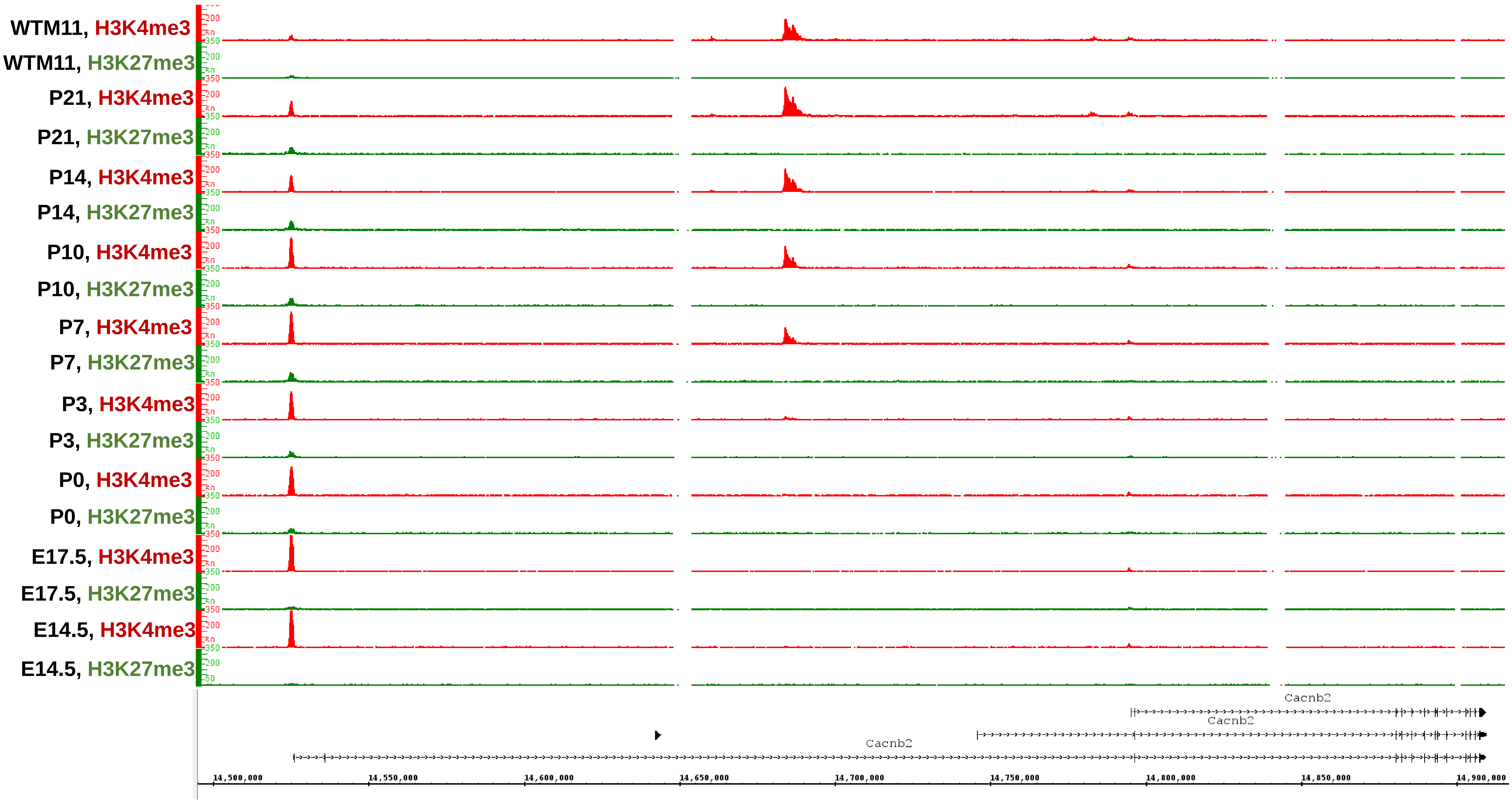
WTM11, H3K4me3
WTM11, H3K27me3
P21, H3K4me3
P21, H3K27me3
P14, H3K4me3
P14, H3K27me3
P10, H3K4me3
P10, H3K27me3
P7, H3K4me3
P7, H3K27me3
P3, H3K4me3
P3, H3K27me3
P0, H3K4me3
P0, H3K27me3
E17.5, H3K4me3
E17.5, H3K27me3
E14.5, H3K4me3
E14.5, H3K27me3
Cacnb2
Cacnb2
Cacnb2
14,500,000
14,550,000
14,600,000
14,650,000
14,700,000
14,750,000
14,800,000
14,850,000
14,900,000

Cdh23

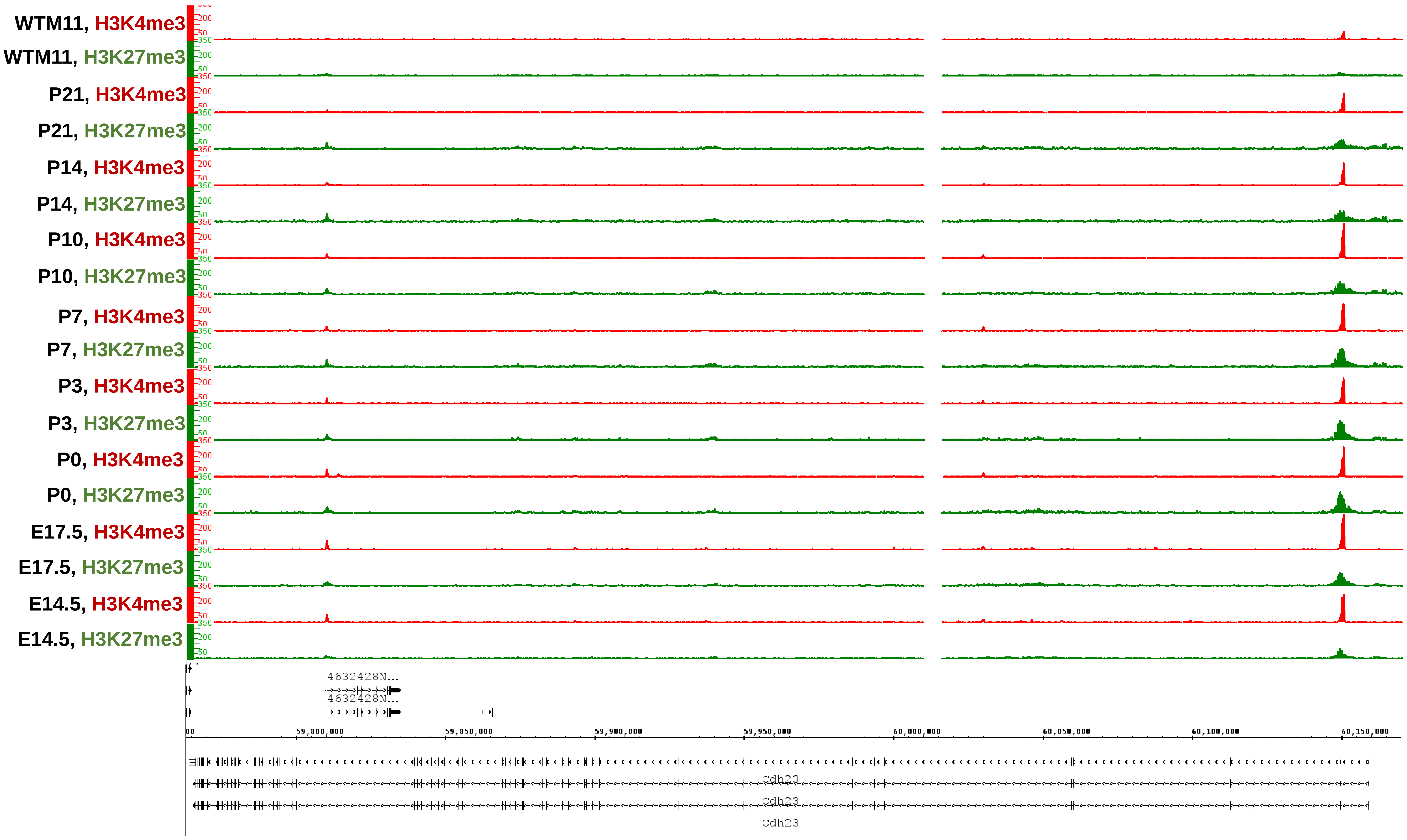
WTM11, H3K4me3
WTM11, H3K27me3
P21, H3K4me3
P21, H3K27me3
P14, H3K4me3
P14, H3K27me3
P10, H3K4me3
P10, H3K27me3
P7, H3K4me3
P7, H3K27me3
P3, H3K4me3
P3, H3K27me3
P0, H3K4me3
P0, H3K27me3
E17.5, H3K4me3
E17.5, H3K27me3
E14.5, H3K4me3
E14.5, H3K27me3
4632428N...
4632428N...
59,800,000
59,850,000
59,900,000
59,950,000
60,000,000
60,050,000
60,100,000
60,150,000
Cdh23
Cdh23
Cdh23

Cds1

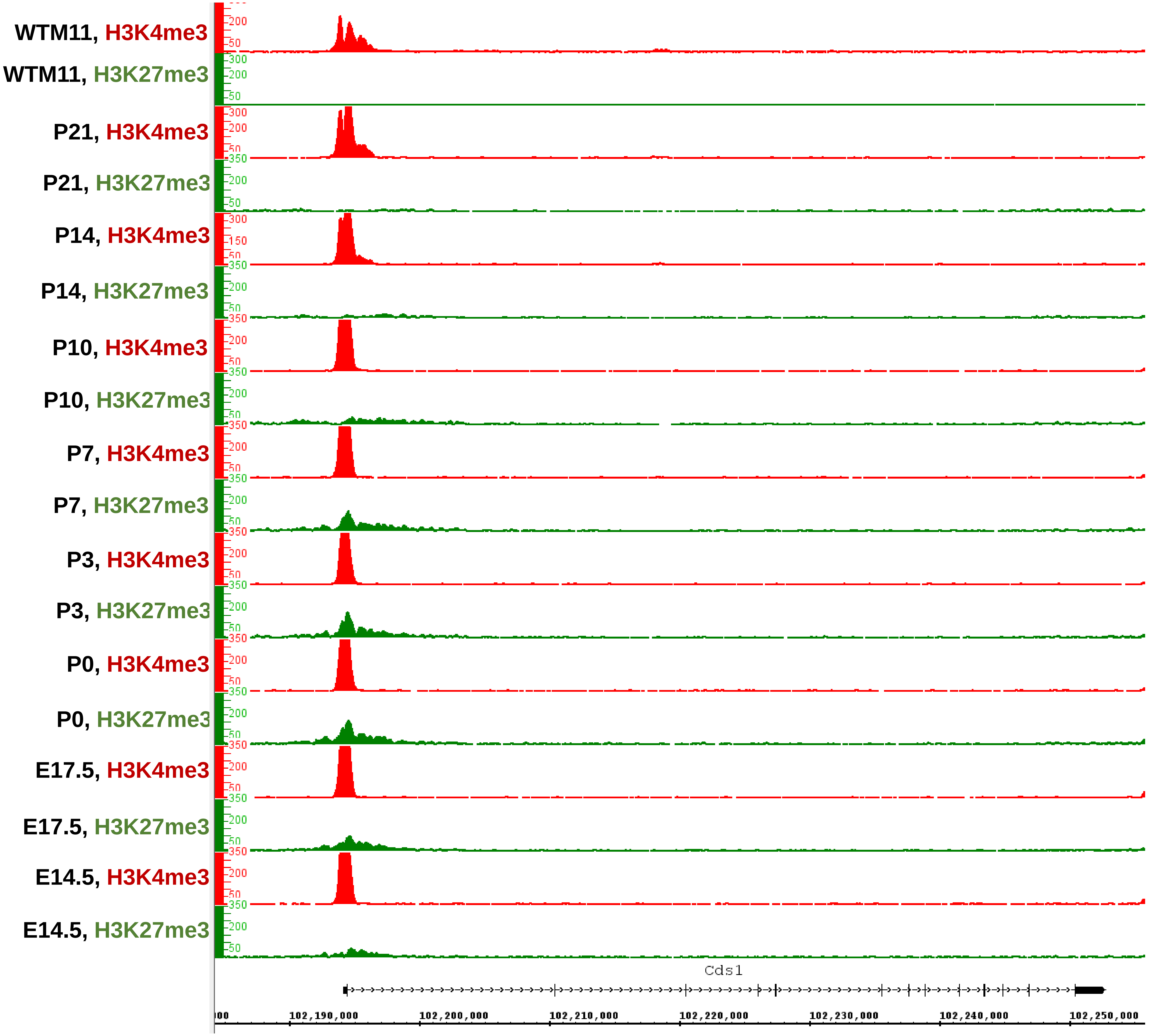
WTM11, H3K4me3
WTM11, H3K27me3
P21, H3K4me3
P21, H3K27me3
P14, H3K4me3
P14, H3K27me3
P10, H3K4me3
P10, H3K27me3
P7, H3K4me3
P7, H3K27me3
P3, H3K4me3
P3, H3K27me3
P0, H3K4me3
P0, H3K27me3
E17.5, H3K4me3
E17.5, H3K27me3
E14.5, H3K4me3
E14.5, H3K27me3
Cds1
102,190,000
102,200,000
102,210,000
102,220,000
102,230,000
102,240,000
102,250,000

Cib2

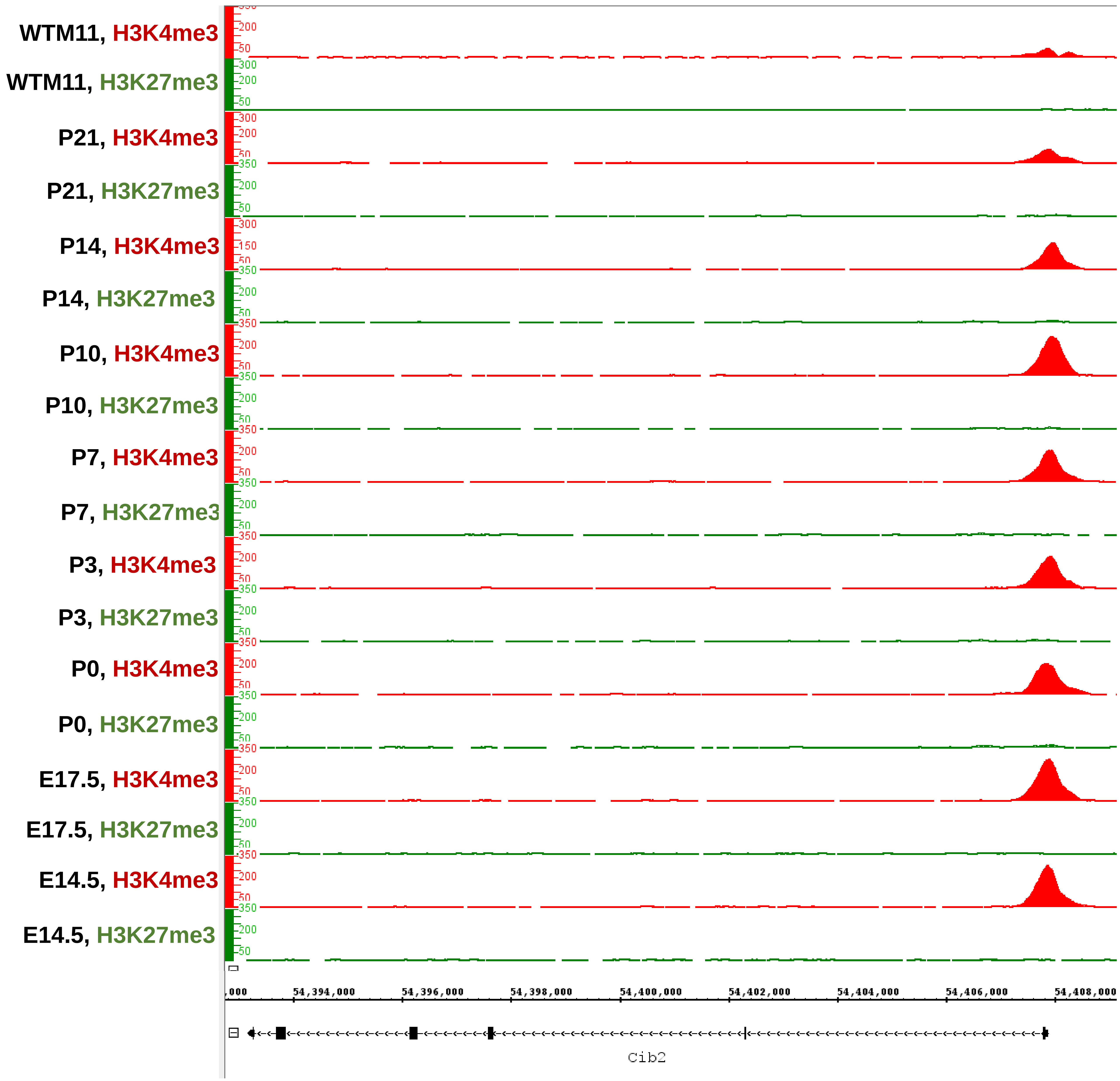

WTM11, H3K4me3
WTM11, H3K27me3
P21, H3K4me3
P21, H3K27me3
P14, H3K4me3
P14, H3K27me3
P10, H3K4me3
P10, H3K27me3
P7, H3K4me3
P7, H3K27me3
P3, H3K4me3
P3, H3K27me3
P0, H3K4me3
P0, H3K27me3
E17.5, H3K4me3
E17.5, H3K27me3
E14.5, H3K4me3
E14.5, H3K27me3
54,394,000
54,396,000
54,398,000
54,400,000
54,402,000
54,404,000
54,406,000
54,408,000
Cib2

Myo3a

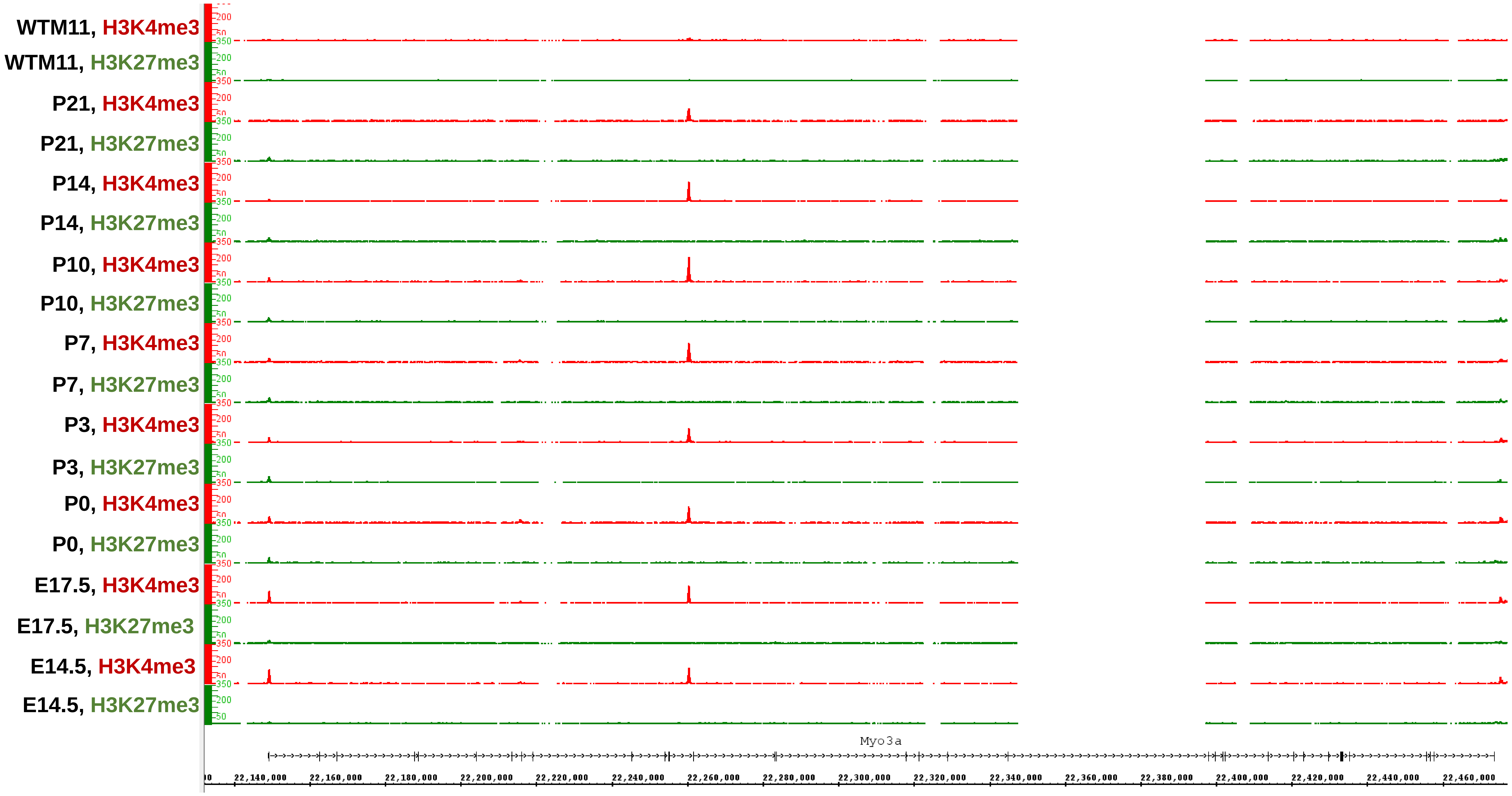
WTM11, H3K4me3
WTM11, H3K27me3
P21, H3K4me3
P21, H3K27me3
P14, H3K4me3
P14, H3K27me3
P10, H3K4me3
P10, H3K27me3
P7, H3K4me3
P7, H3K27me3
P3, H3K4me3
P3, H3K27me3
P0, H3K4me3
P0, H3K27me3
E17.5, H3K4me3
E17.5, H3K27me3
E14.5, H3K4me3
E14.5, H3K27me3
Myo3a
22,140,000
22,160,000
22,180,000
22,200,000
22,220,000
22,240,000
22,260,000
22,280,000
22,300,000
22,320,000
22,340,000
22,360,000
22,380,000
22,400,000
22,420,000
22,440,000
22,460,000

Myrip

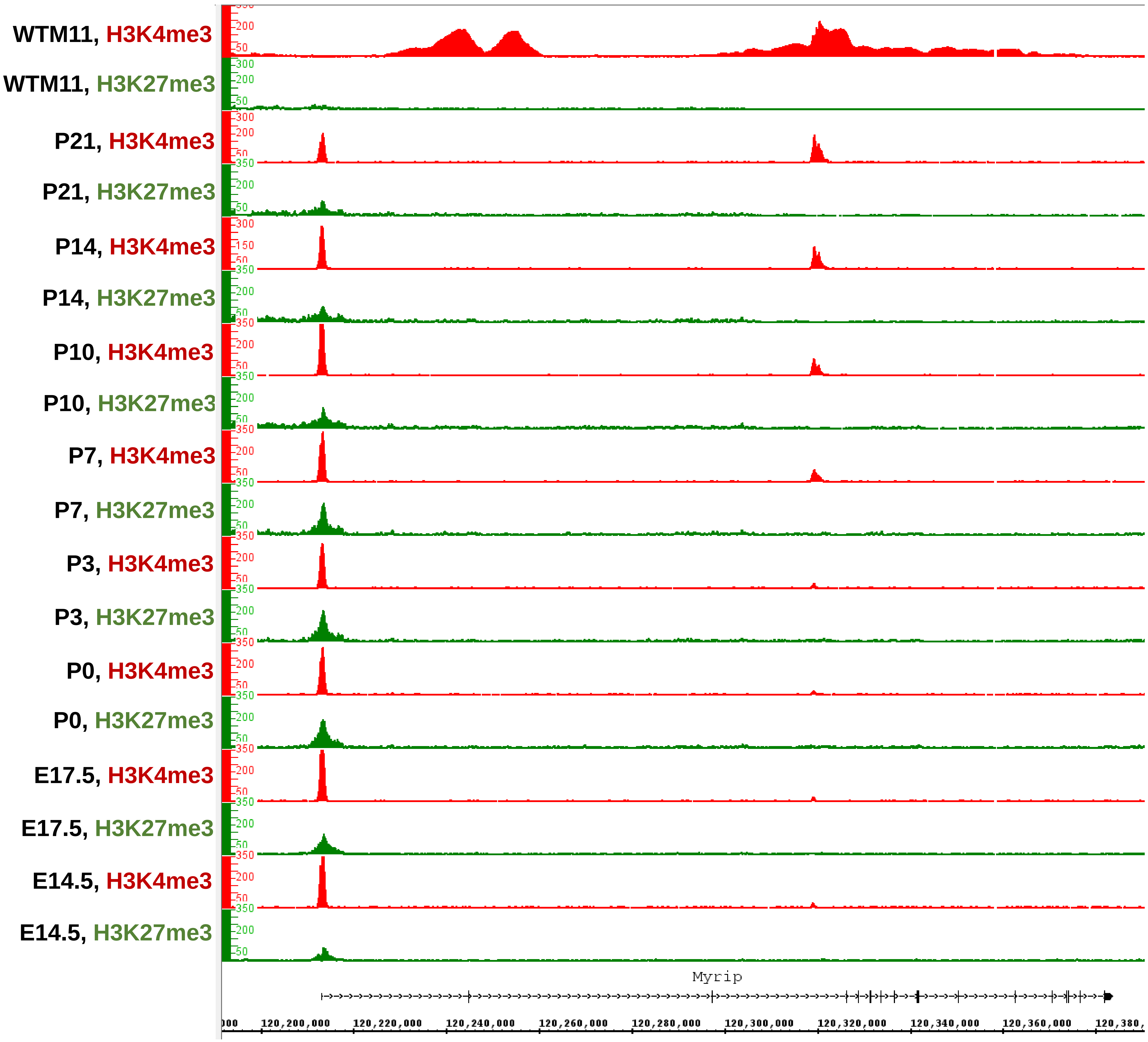
WTM11, H3K4me3
WTM11, H3K27me3
P21, H3K4me3
P21, H3K27me3
P14, H3K4me3
P14, H3K27me3
P10, H3K4me3
P10, H3K27me3
P7, H3K4me3
P7, H3K27me3
P3, H3K4me3
P3, H3K27me3
P0, H3K4me3
P0, H3K27me3
E17.5, H3K4me3
E17.5, H3K27me3
E14.5, H3K4me3
E14.5, H3K27me3
Myrip
120,200,000
120,220,000
120,240,000
120,260,000
120,280,000
120,300,000
120,320,000
120,340,000
120,360,000

Ptgs1

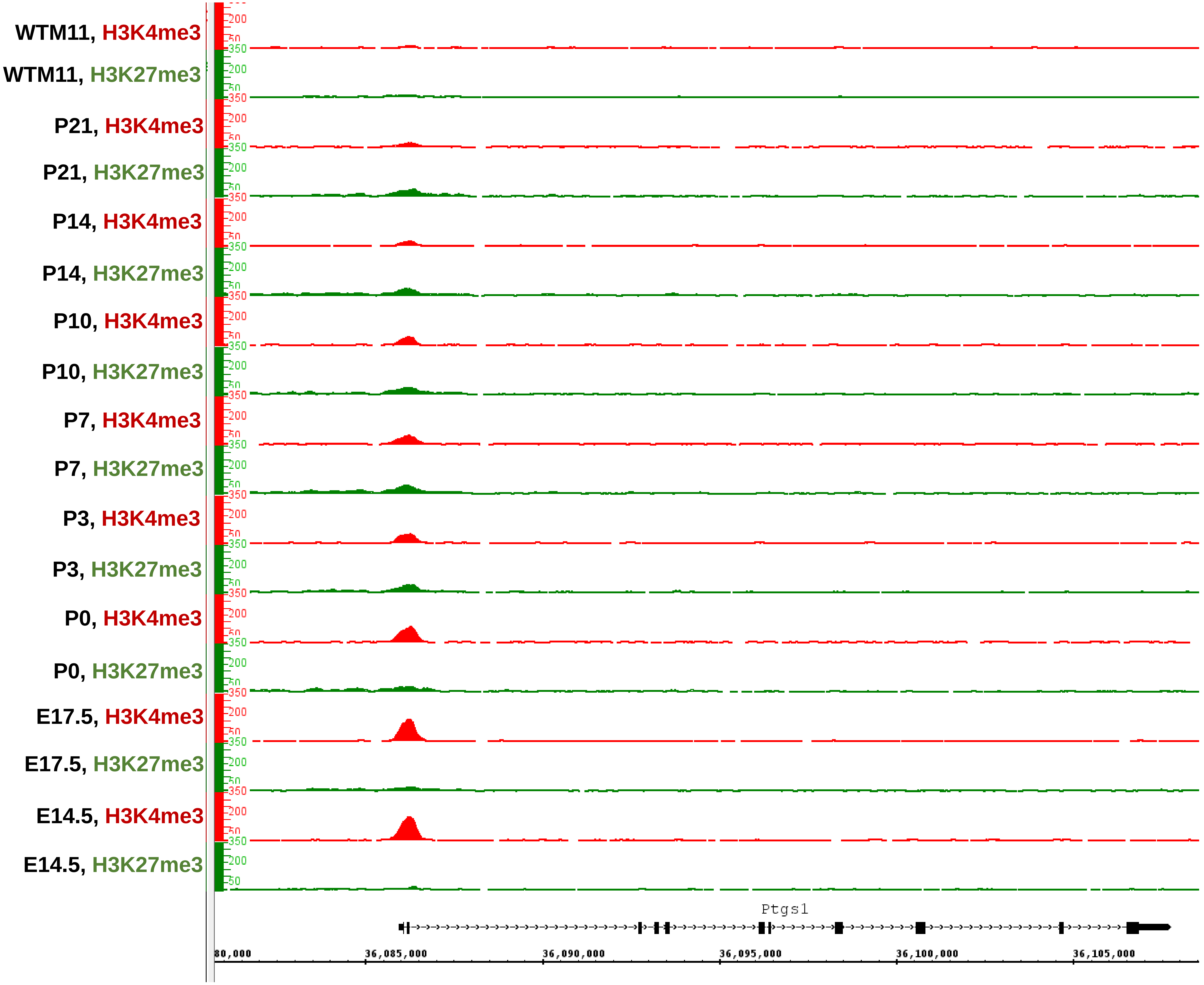
WTM11, H3K4me3
WTM11, H3K27me3
P21, H3K4me3
P21, H3K27me3
P14, H3K4me3
P14, H3K27me3
P10, H3K4me3
P10, H3K27me3
P7, H3K4me3
P7, H3K27me3
P3, H3K4me3
P3, H3K27me3
P0, H3K4me3
P0, H3K27me3
E17.5, H3K4me3
E17.5, H3K27me3
E14.5, H3K4me3
E14.5, H3K27me3
350
200
50
Ptgs1
80,000
36,085,000
36,090,000
36,095,000
36,100,000
36,105,000

Ptprk

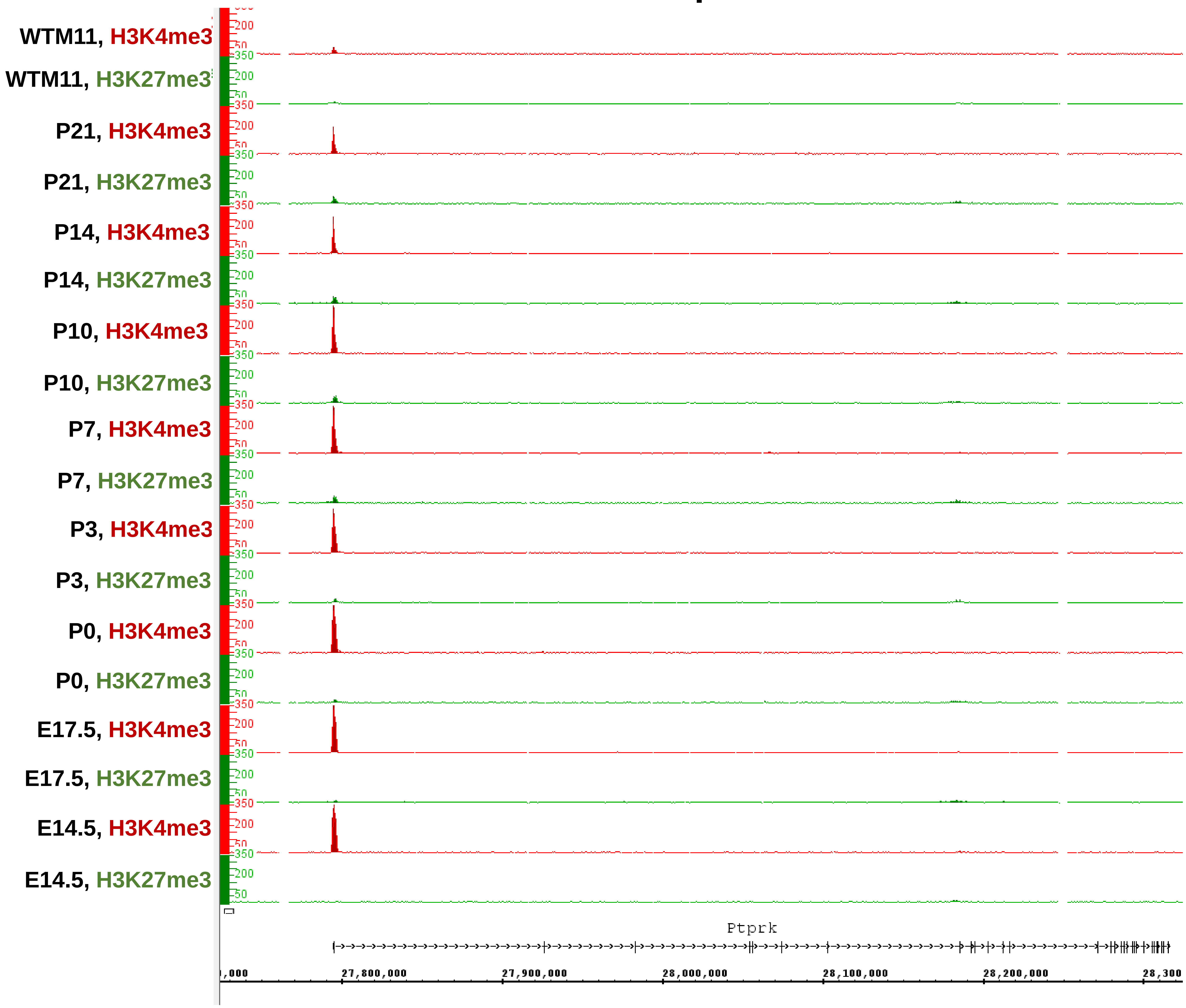
WTM11, H3K4me3
WTM11, H3K27me3
P21, H3K4me3
P21, H3K27me3
P14, H3K4me3
P14, H3K27me3
P10, H3K4me3
P10, H3K27me3
P7, H3K4me3
P7, H3K27me3
P3, H3K4me3
P3, H3K27me3
P0, H3K4me3
P0, H3K27me3
E17.5, H3K4me3
E17.5, H3K27me3
E14.5, H3K4me3
E14.5, H3K27me3
Ptprk
27,800,000
27,900,000
28,000,000
28,100,000
28,200,000
28,300

Rab27a

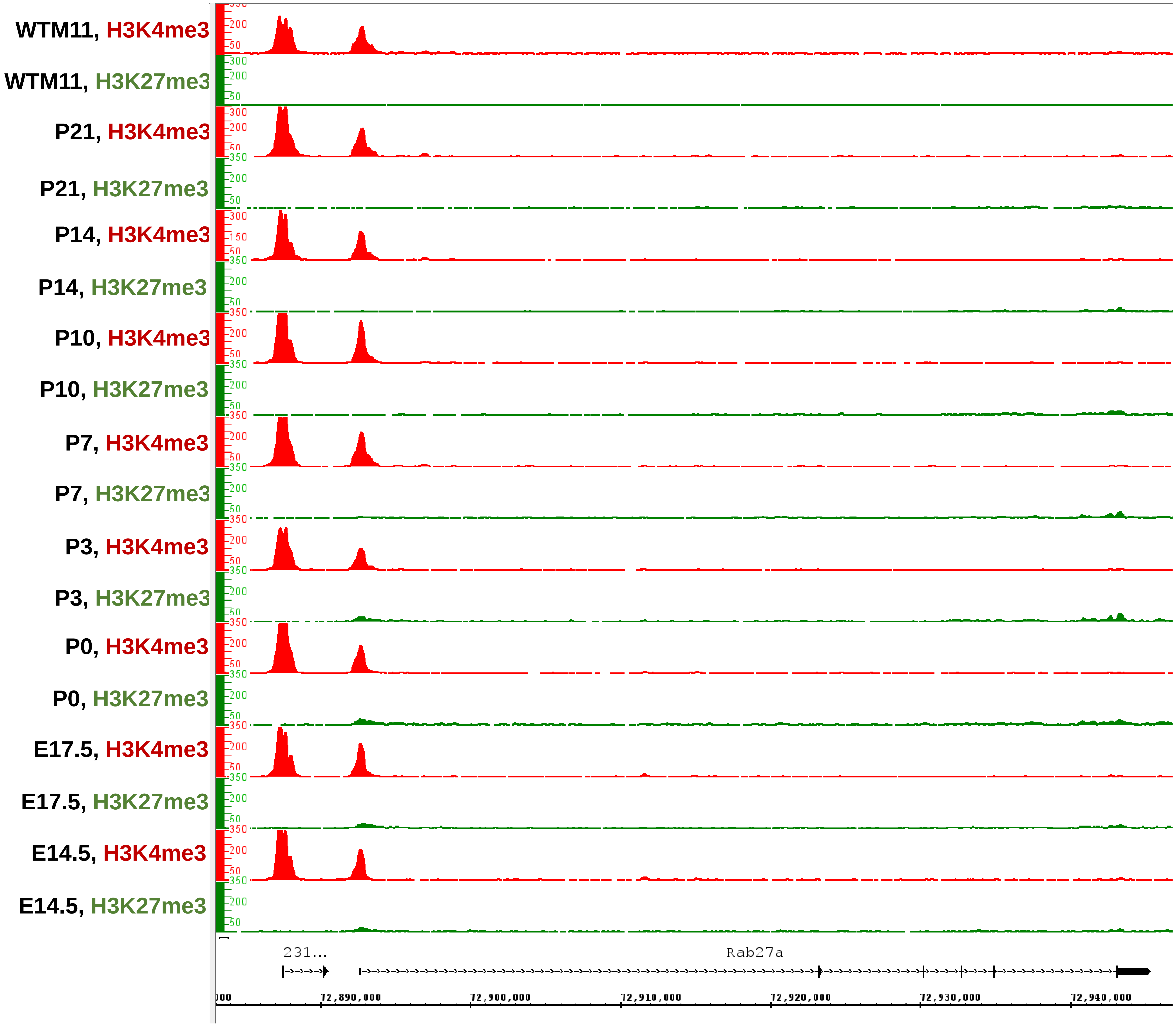

WTM11, H3K4me3
WTM11, H3K27me3
P21, H3K4me3
P21, H3K27me3
P14, H3K4me3
P14, H3K27me3
P10, H3K4me3
P10, H3K27me3
P7, H3K4me3
P7, H3K27me3
P3, H3K4me3
P3, H3K27me3
P0, H3K4me3
P0, H3K27me3
E17.5, H3K4me3
E17.5, H3K27me3
E14.5, H3K4me3
E14.5, H3K27me3
231...
Rab27a
72,890,000
72,900,000
72,910,000
72,920,000
72,930,000
72,940,000

Rapgef4

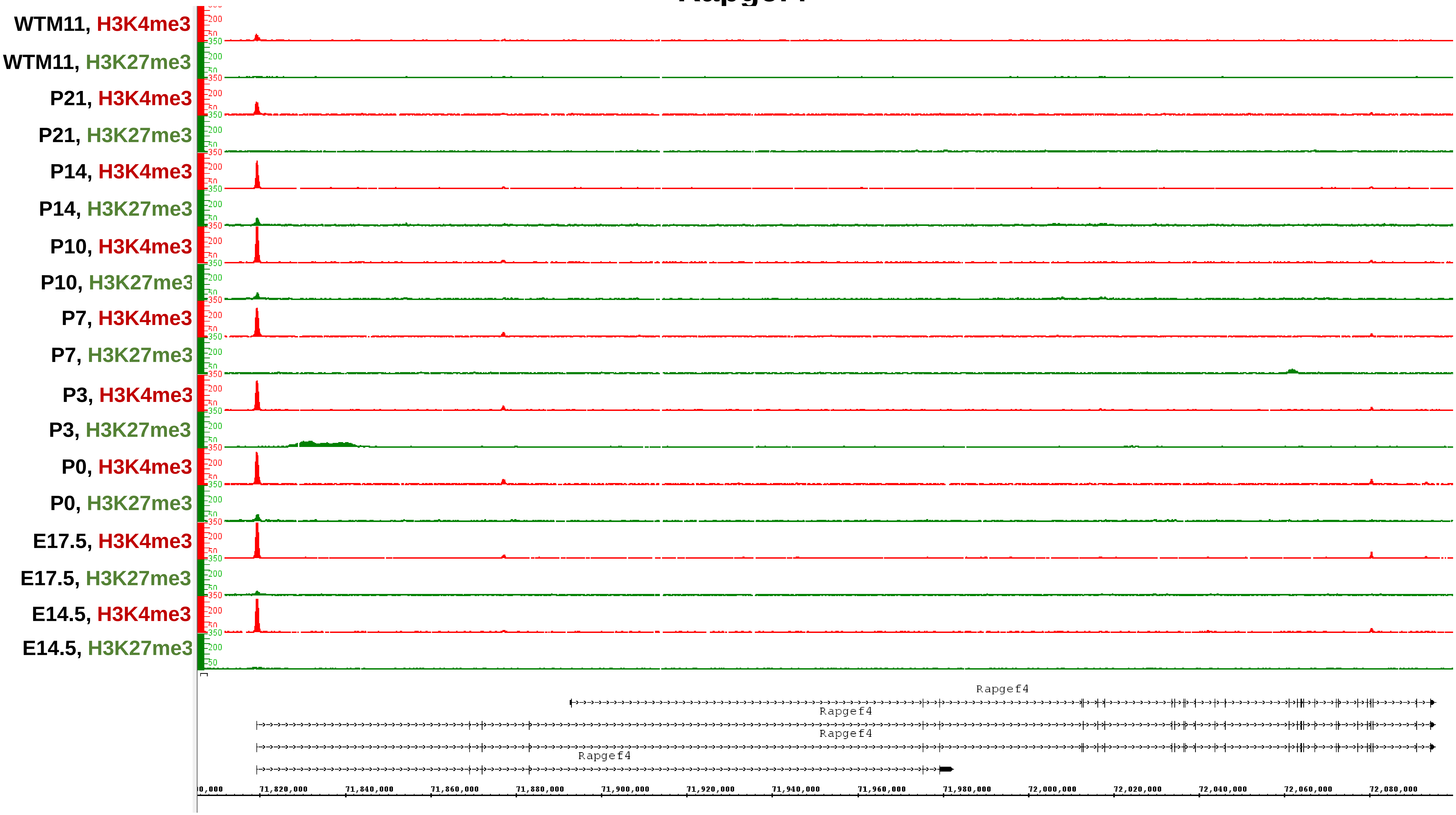

WTM11, H3K4me3
WTM11, H3K27me3
P21, H3K4me3
P21, H3K27me3
P14, H3K4me3
P14, H3K27me3
P10, H3K4me3
P10, H3K27me3
P7, H3K4me3
P7, H3K27me3
P3, H3K4me3
P3, H3K27me3
P0, H3K4me3
P0, H3K27me3
E17.5, H3K4me3
E17.5, H3K27me3
E14.5, H3K4me3
E14.5, H3K27me3
350
200
50
Rapgef4
Rapgef4
Rapgef4
Rapgef4
71,820,000
71,840,000
71,860,000
71,880,000
71,900,000
71,920,000
71,940,000
71,960,000
71,980,000
72,000,000
72,020,000
72,040,000
72,060,000
72,080,000

Rgs9bp

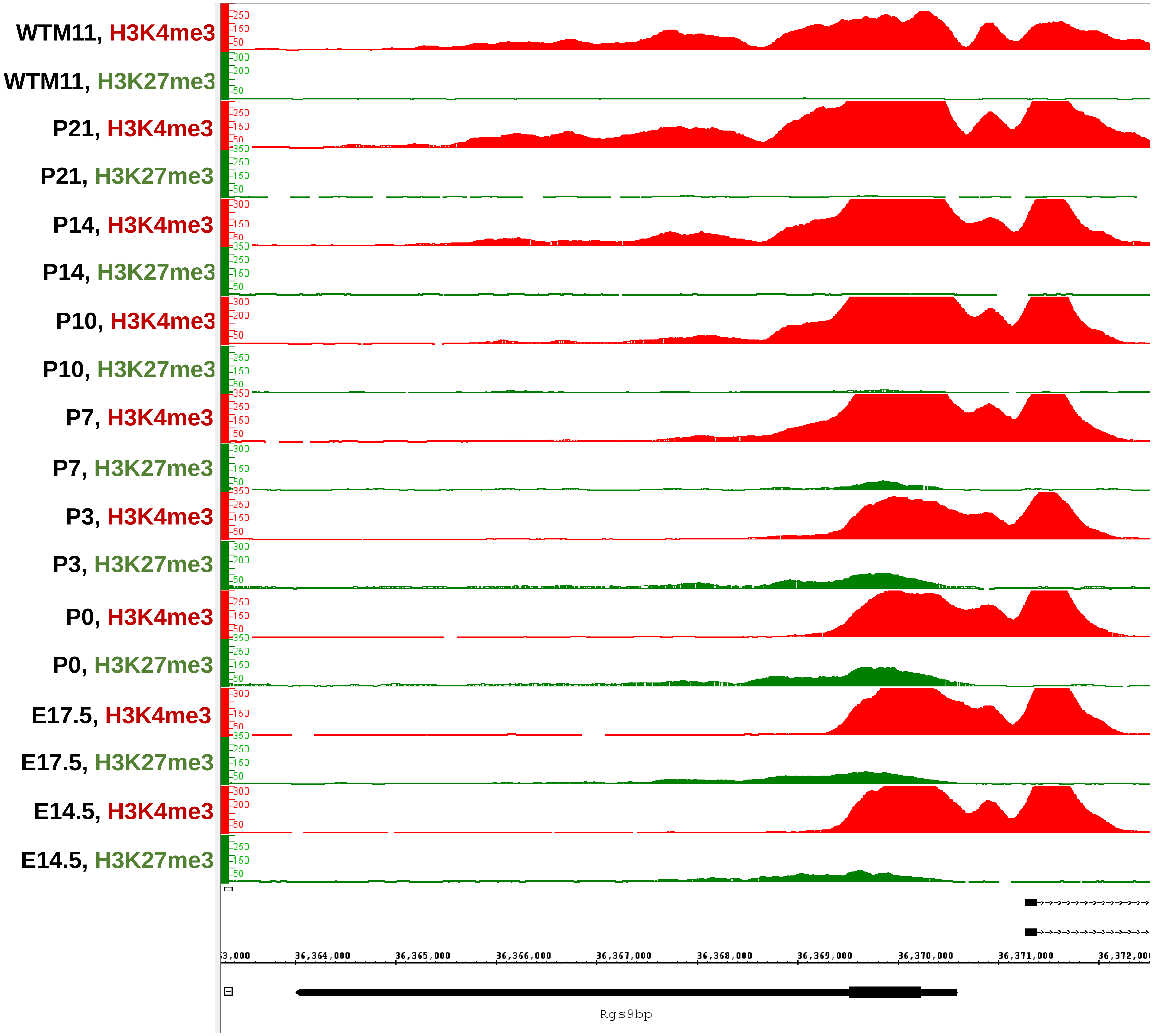
WTM11, H3K4me3
WTM11, H3K27me3
P21, H3K4me3
P21, H3K27me3
P14, H3K4me3
P14, H3K27me3
P10, H3K4me3
P10, H3K27me3
P7, H3K4me3
P7, H3K27me3
P3, H3K4me3
P3, H3K27me3
P0, H3K4me3
P0, H3K27me3
E17.5, H3K4me3
E17.5, H3K27me3
E14.5, H3K4me3
E14.5, H3K27me3
36,364,000
36,365,000
36,366,000
36,367,000
36,368,000
36,369,000
36,370,000
36,371,000
Rgs9bp

Shank2

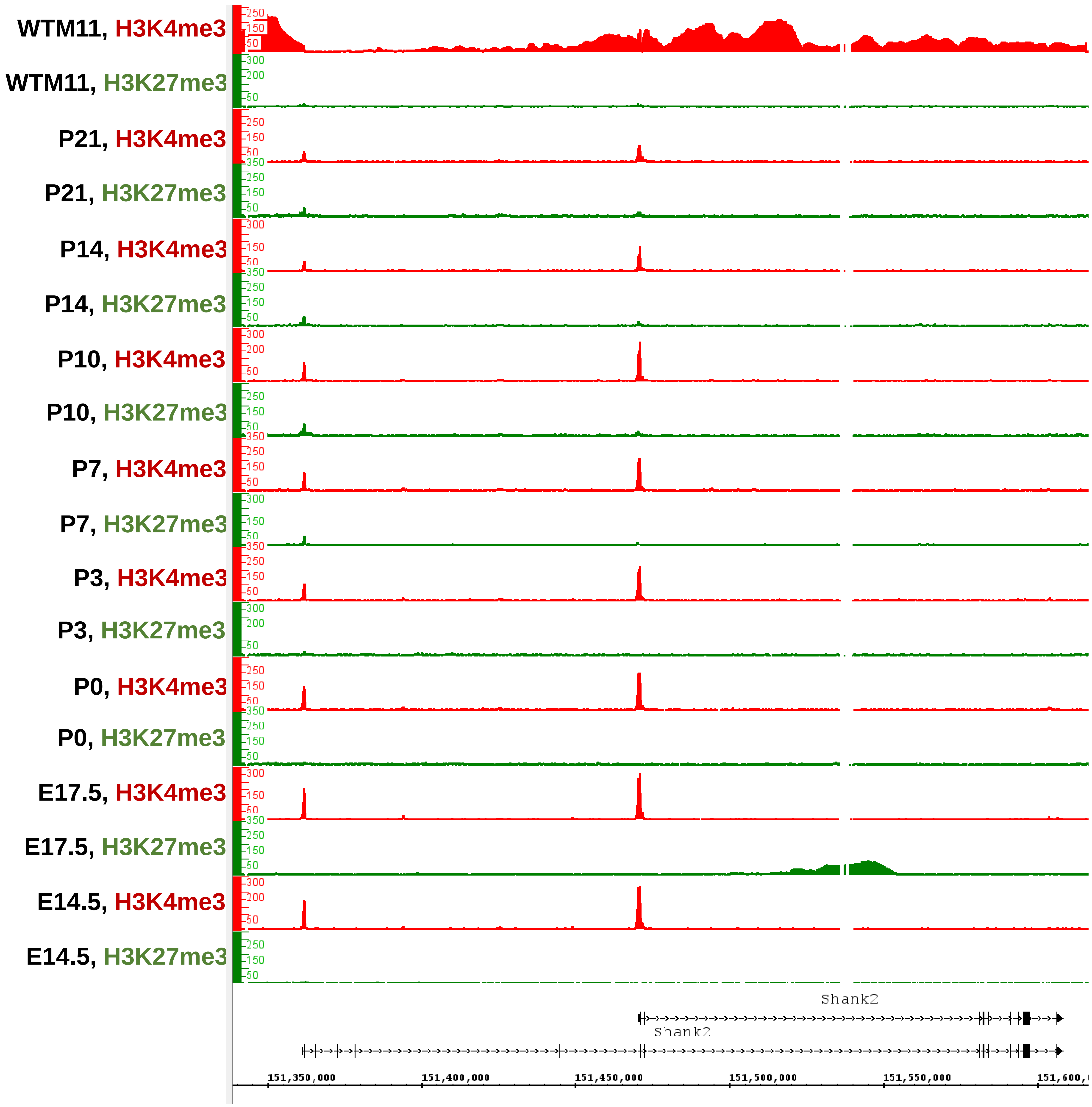
WTM11, H3K4me3
WTM11, H3K27me3
P21, H3K4me3
P21, H3K27me3
P14, H3K4me3
P14, H3K27me3
P10, H3K4me3
P10, H3K27me3
P7, H3K4me3
P7, H3K27me3
P3, H3K4me3
P3, H3K27me3
P0, H3K4me3
P0, H3K27me3
E17.5, H3K4me3
E17.5, H3K27me3
E14.5, H3K4me3
E14.5, H3K27me3
Shank2
Shank2
151,350,000
151,400,000
151,450,000
151,500,000
151,550,000

Slc24a2

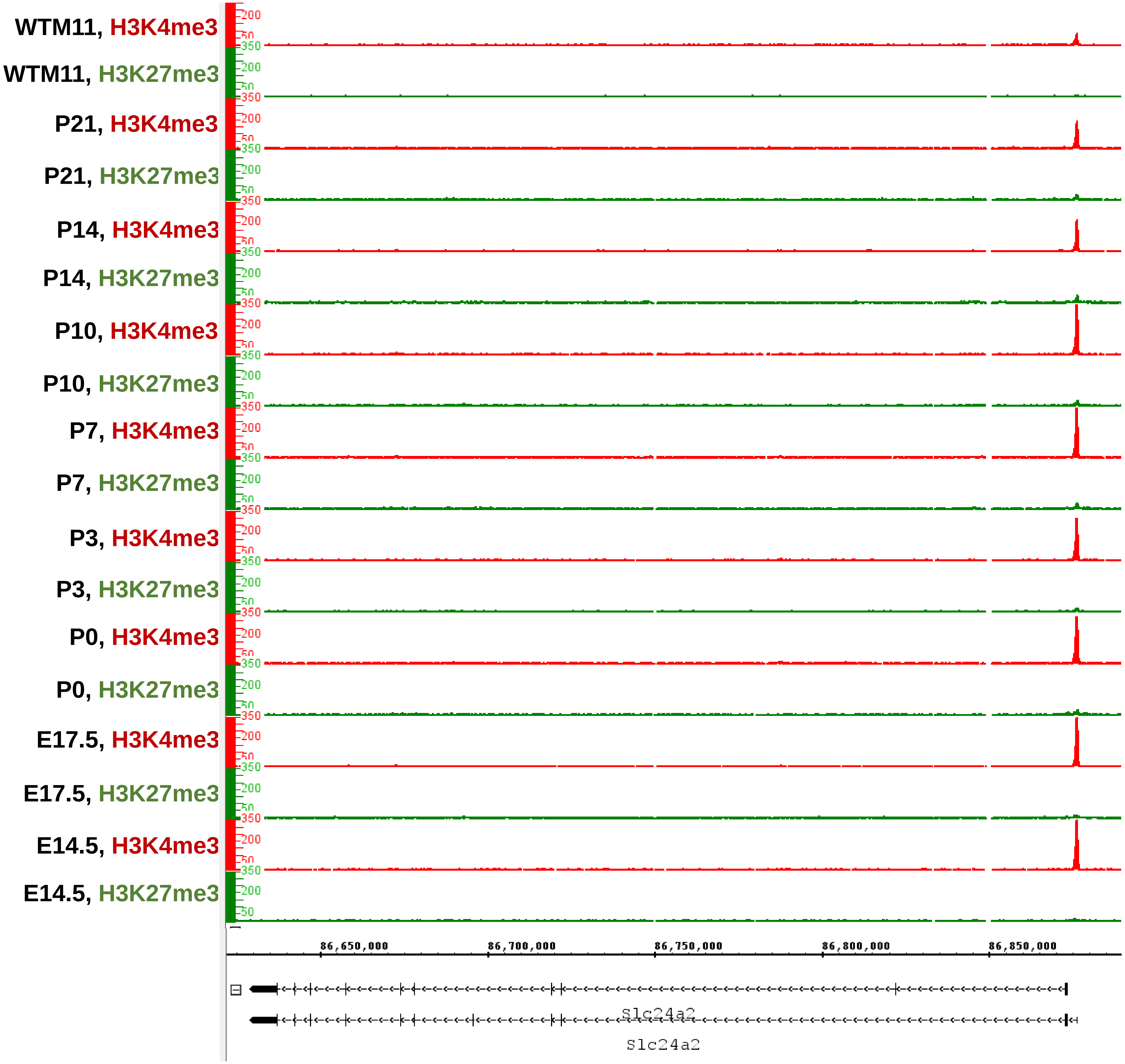
WTM11, H3K4me3
WTM11, H3K27me3
P21, H3K4me3
P21, H3K27me3
P14, H3K4me3
P14, H3K27me3
P10, H3K4me3
P10, H3K27me3
P7, H3K4me3
P7, H3K27me3
P3, H3K4me3
P3, H3K27me3
P0, H3K4me3
P0, H3K27me3
E17.5, H3K4me3
E17.5, H3K27me3
E14.5, H3K4me3
E14.5, H3K27me3
86,650,000
86,700,000
86,750,000
86,800,000
86,850,000
Slc24a2
Slc24a2

Trpc3

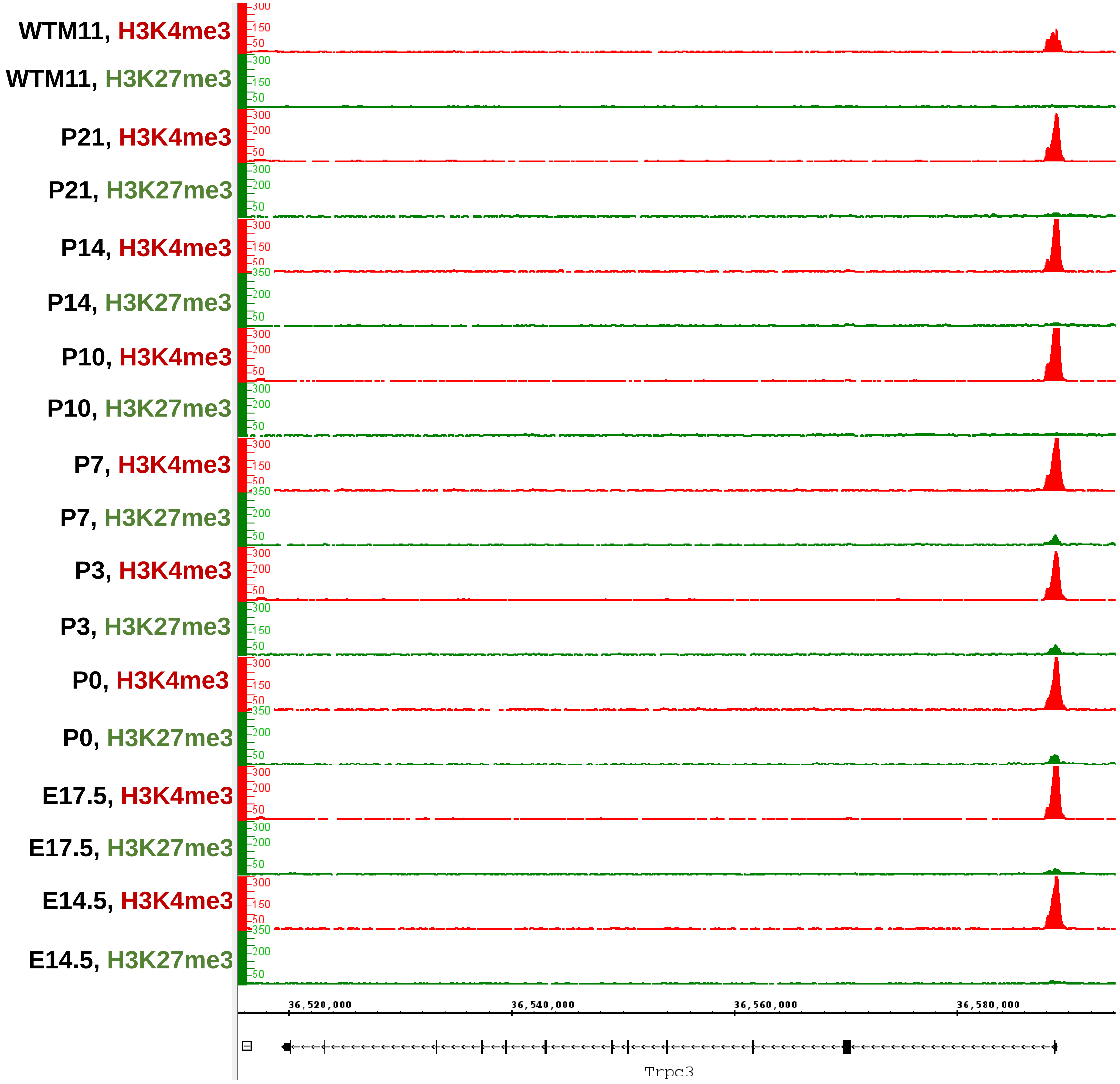

WTM11, H3K4me3
WTM11, H3K27me3
P21, H3K4me3
P21, H3K27me3
P14, H3K4me3
P14, H3K27me3
P10, H3K4me3
P10, H3K27me3
P7, H3K4me3
P7, H3K27me3
P3, H3K4me3
P3, H3K27me3
P0, H3K4me3
P0, H3K27me3
E17.5, H3K4me3
E17.5, H3K27me3
E14.5, H3K4me3
E14.5, H3K27me3
36,520,000
36,540,000
36,560,000
36,580,000
Trpc3

Vcan

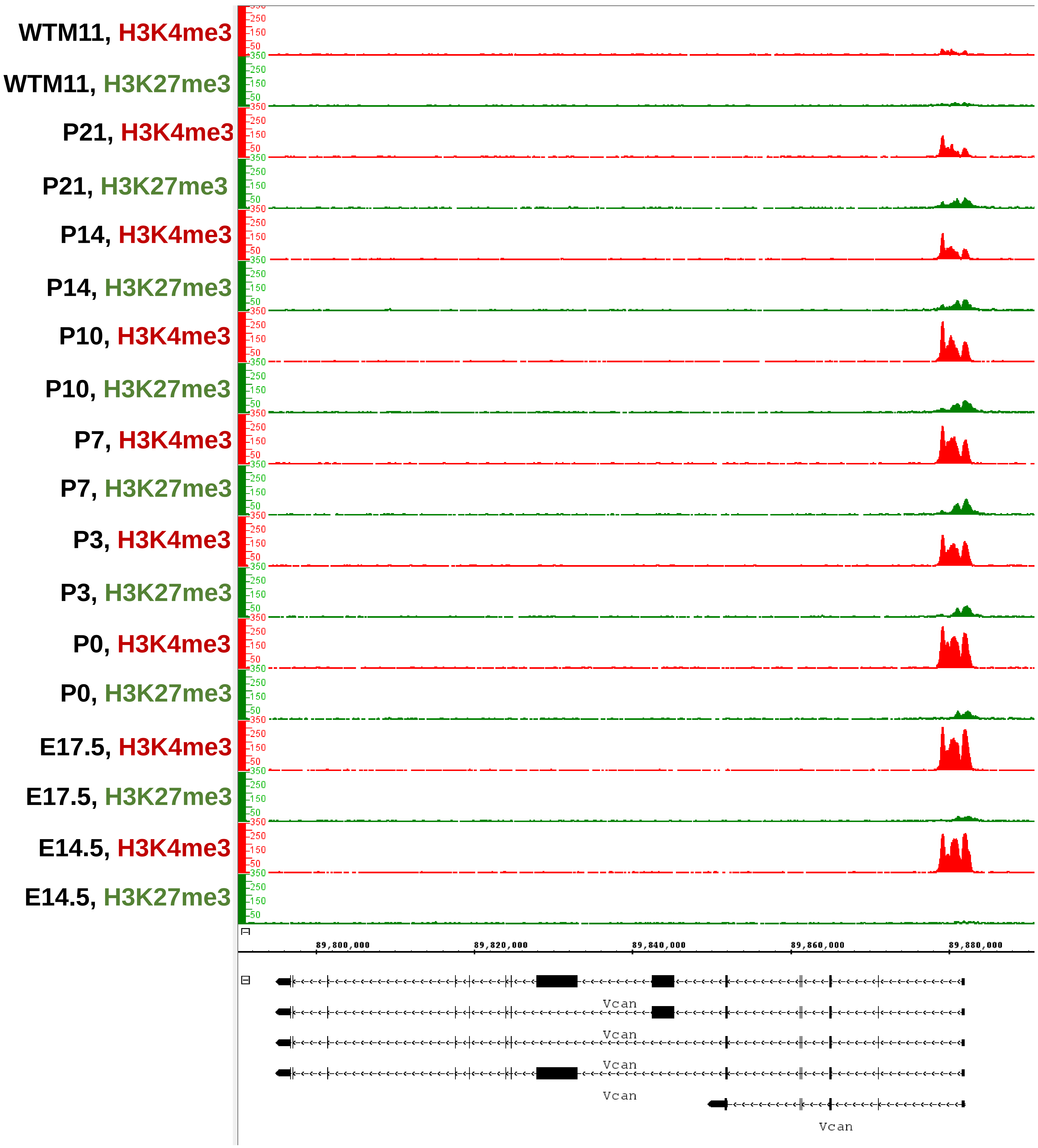
WTM11, H3K4me3
WTM11, H3K27me3
P21, H3K4me3
P21, H3K27me3
P14, H3K4me3
P14, H3K27me3
P10, H3K4me3
P10, H3K27me3
P7, H3K4me3
P7, H3K27me3
P3, H3K4me3
P3, H3K27me3
P0, H3K4me3
P0, H3K27me3
E17.5, H3K4me3
E17.5, H3K27me3
E14.5, H3K4me3
E14.5, H3K27me3
350
250
150
50
89,800,000
89,820,000
89,840,000
89,860,000
89,880,000
Vcan
Vcan
Vcan
Vcan
Vcan

Grm6

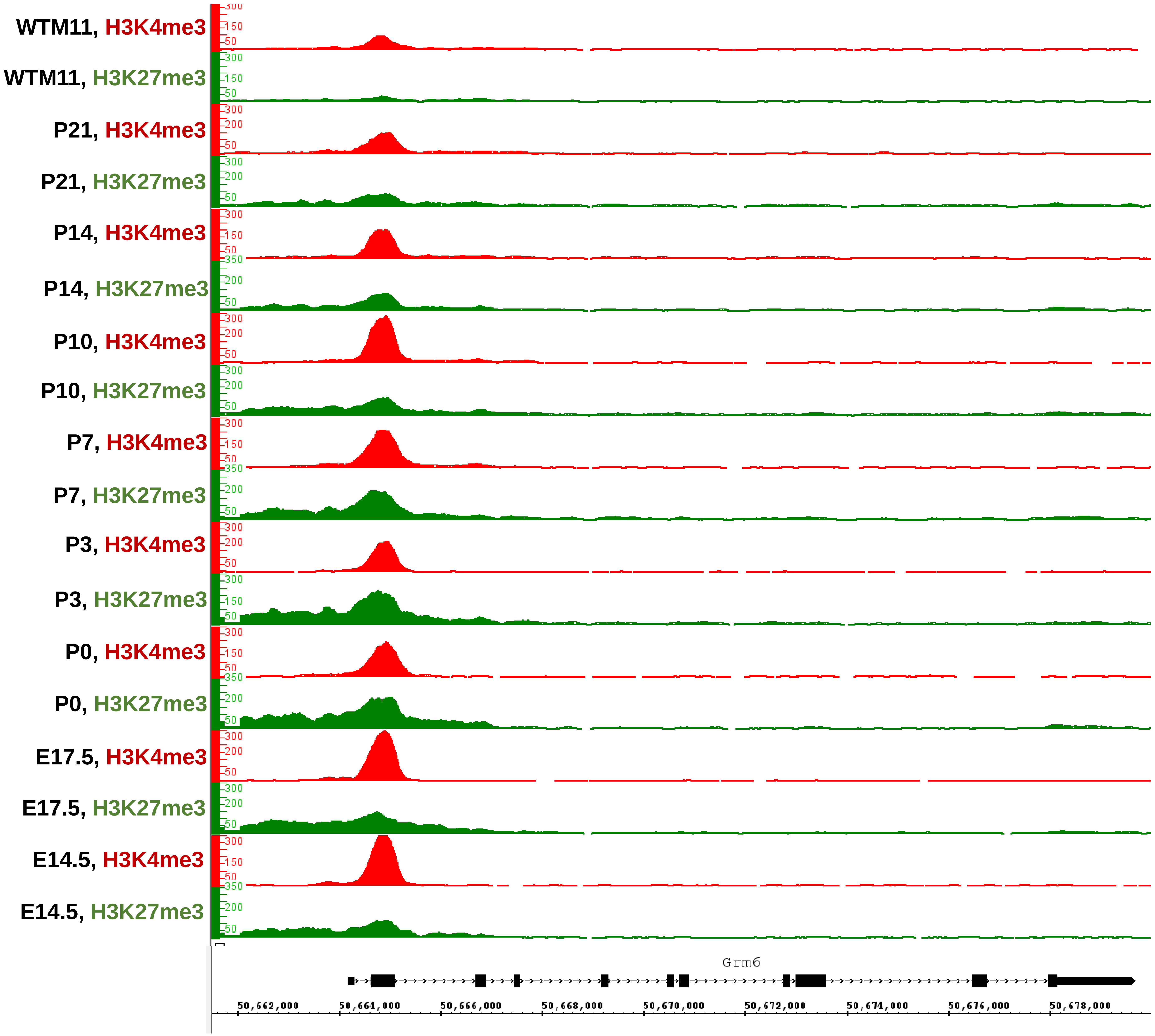
WTM11, H3K4me3
WTM11, H3K27me3
P21, H3K4me3
P21, H3K27me3
P14, H3K4me3
P14, H3K27me3
P10, H3K4me3
P10, H3K27me3
P7, H3K4me3
P7, H3K27me3
P3, H3K4me3
P3, H3K27me3
P0, H3K4me3
P0, H3K27me3
E17.5, H3K4me3
E17.5, H3K27me3
E14.5, H3K4me3
E14.5, H3K27me3
Grm6
50,662,000
50,664,000
50,666,000
50,668,000
50,670,000
50,672,000
50,674,000
50,676,000
50,678,000

Guca1a

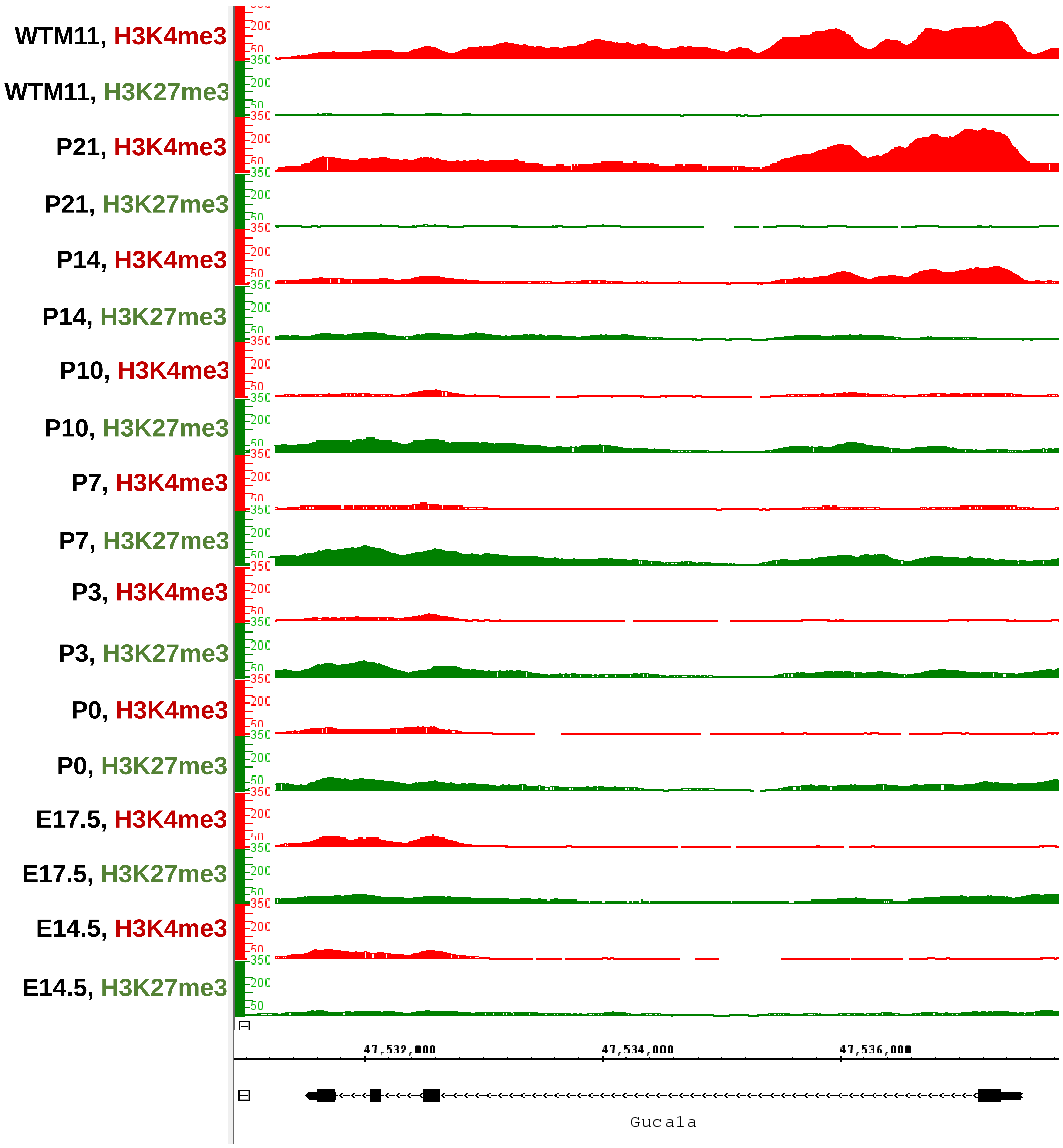

WTM11, H3K4me3
WTM11, H3K27me3
P21, H3K4me3
P21, H3K27me3
P14, H3K4me3
P14, H3K27me3
P10, H3K4me3
P10, H3K27me3
P7, H3K4me3
P7, H3K27me3
P3, H3K4me3
P3, H3K27me3
P0, H3K4me3
P0, H3K27me3
E17.5, H3K4me3
E17.5, H3K27me3
E14.5, H3K4me3
E14.5, H3K27me3
350
200
50
47,532,000
47,534,000
47,536,000
Guca1a

Lrat

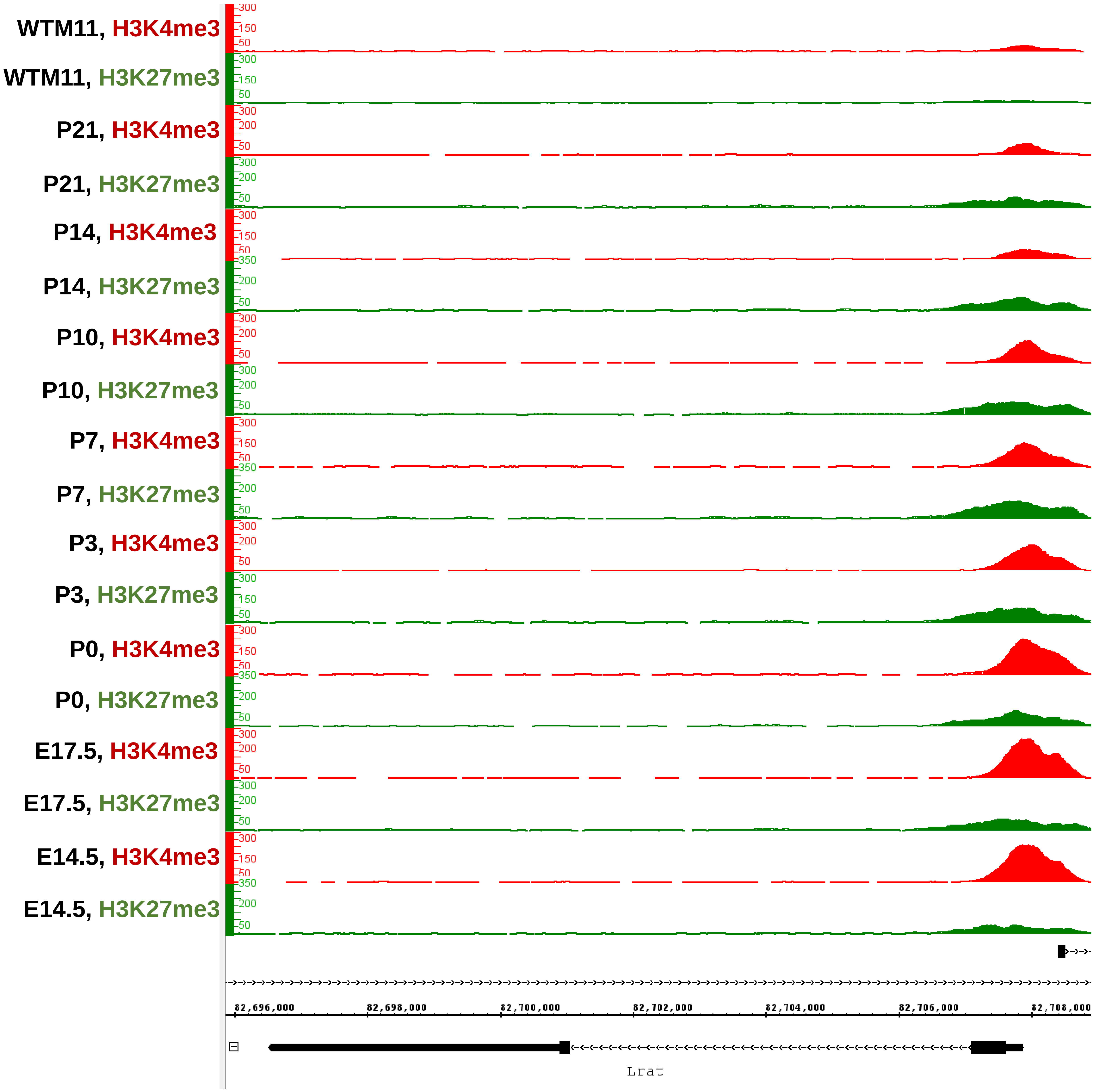
WTM11, H3K4me3
WTM11, H3K27me3
P21, H3K4me3
P21, H3K27me3
P14, H3K4me3
P14, H3K27me3
P10, H3K4me3
P10, H3K27me3
P7, H3K4me3
P7, H3K27me3
P3, H3K4me3
P3, H3K27me3
P0, H3K4me3
P0, H3K27me3
E17.5, H3K4me3
E17.5, H3K27me3
E14.5, H3K4me3
E14.5, H3K27me3
82,696,000
82,698,000
82,700,000
82,702,000
82,704,000
82,706,000
82,708,000
Lrat

Prdm13

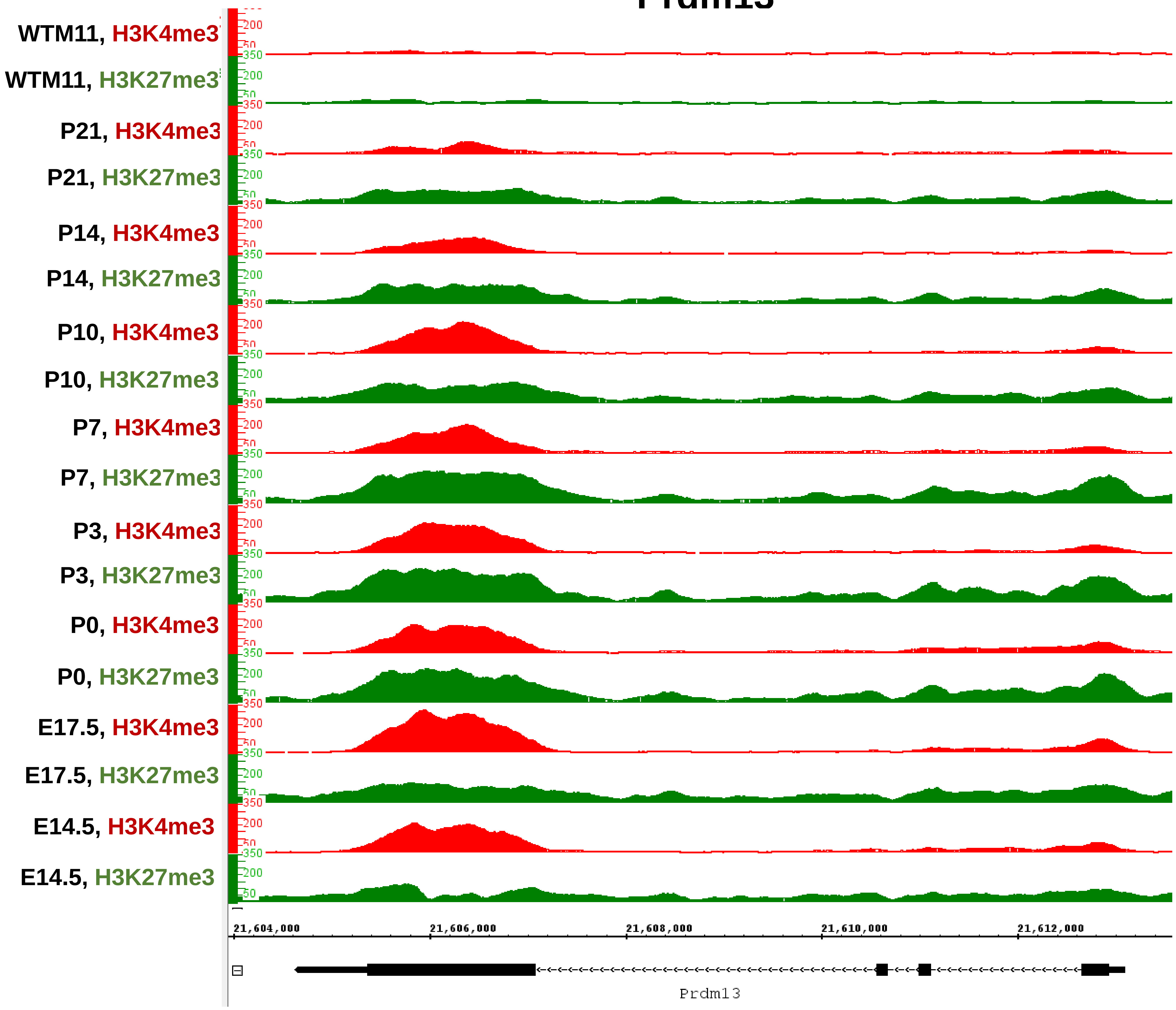
WTM11, H3K4me3
WTM11, H3K27me3
P21, H3K4me3
P21, H3K27me3
P14, H3K4me3
P14, H3K27me3
P10, H3K4me3
P10, H3K27me3
P7, H3K4me3
P7, H3K27me3
P3, H3K4me3
P3, H3K27me3
P0, H3K4me3
P0, H3K27me3
E17.5, H3K4me3
E17.5, H3K27me3
E14.5, H3K4me3
E14.5, H3K27me3
350
200
50
21,604,000
21,606,000
21,608,000
21,610,000
21,612,000
Prdm13

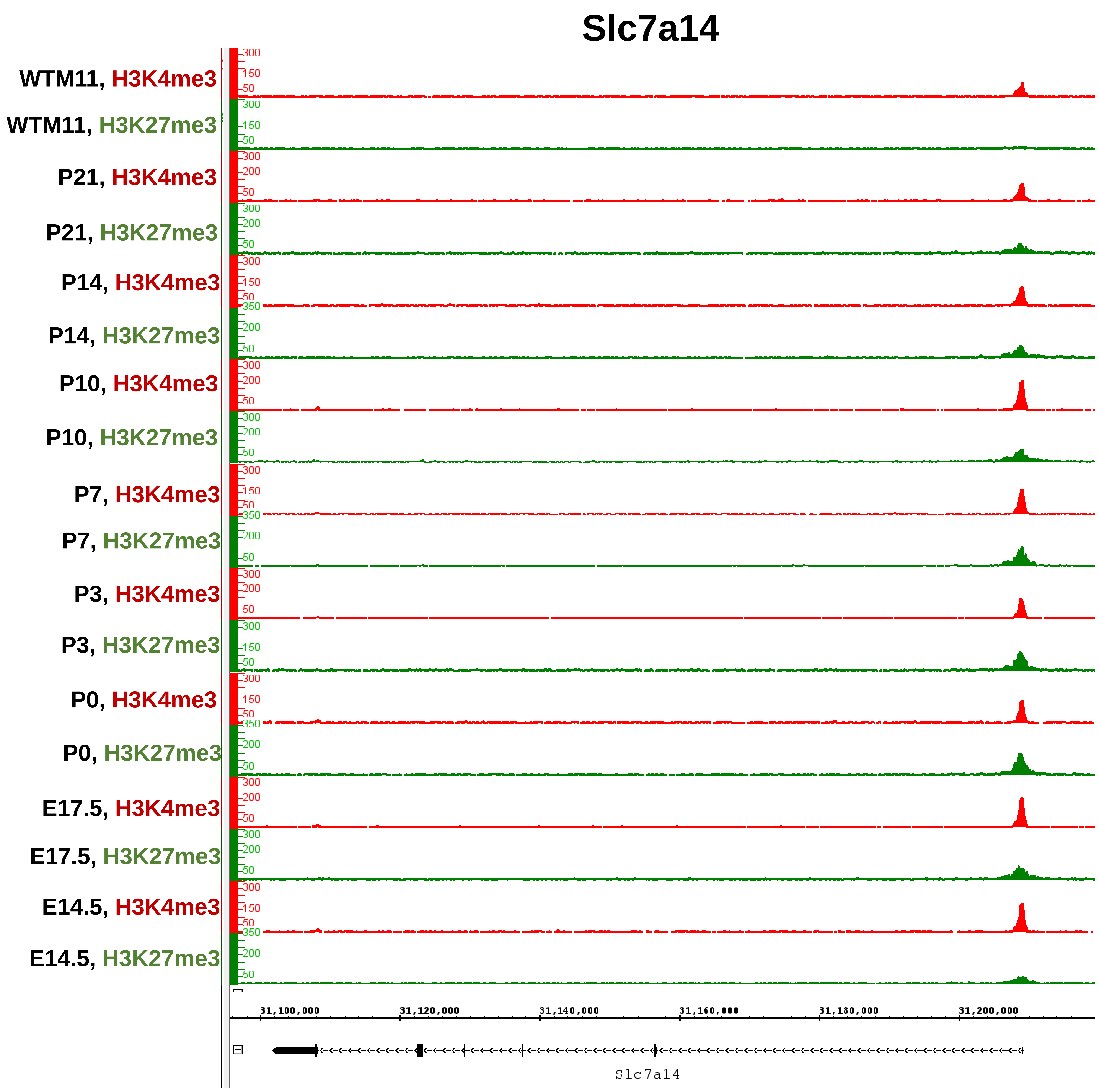

Slc7a14
WTM11, H3K4me3
WTM11, H3K27me3
P21, H3K4me3
P21, H3K27me3
P14, H3K4me3
P14, H3K27me3
P10, H3K4me3
P10, H3K27me3
P7, H3K4me3
P7, H3K27me3
P3, H3K4me3
P3, H3K27me3
P0, H3K4me3
P0, H3K27me3
E17.5, H3K4me3
E17.5, H3K27me3
E14.5, H3K4me3
E14.5, H3K27me3
31,100,000
31,120,000
31,140,000
31,160,000
31,180,000
31,200,000
Slc7a14

Tulp1

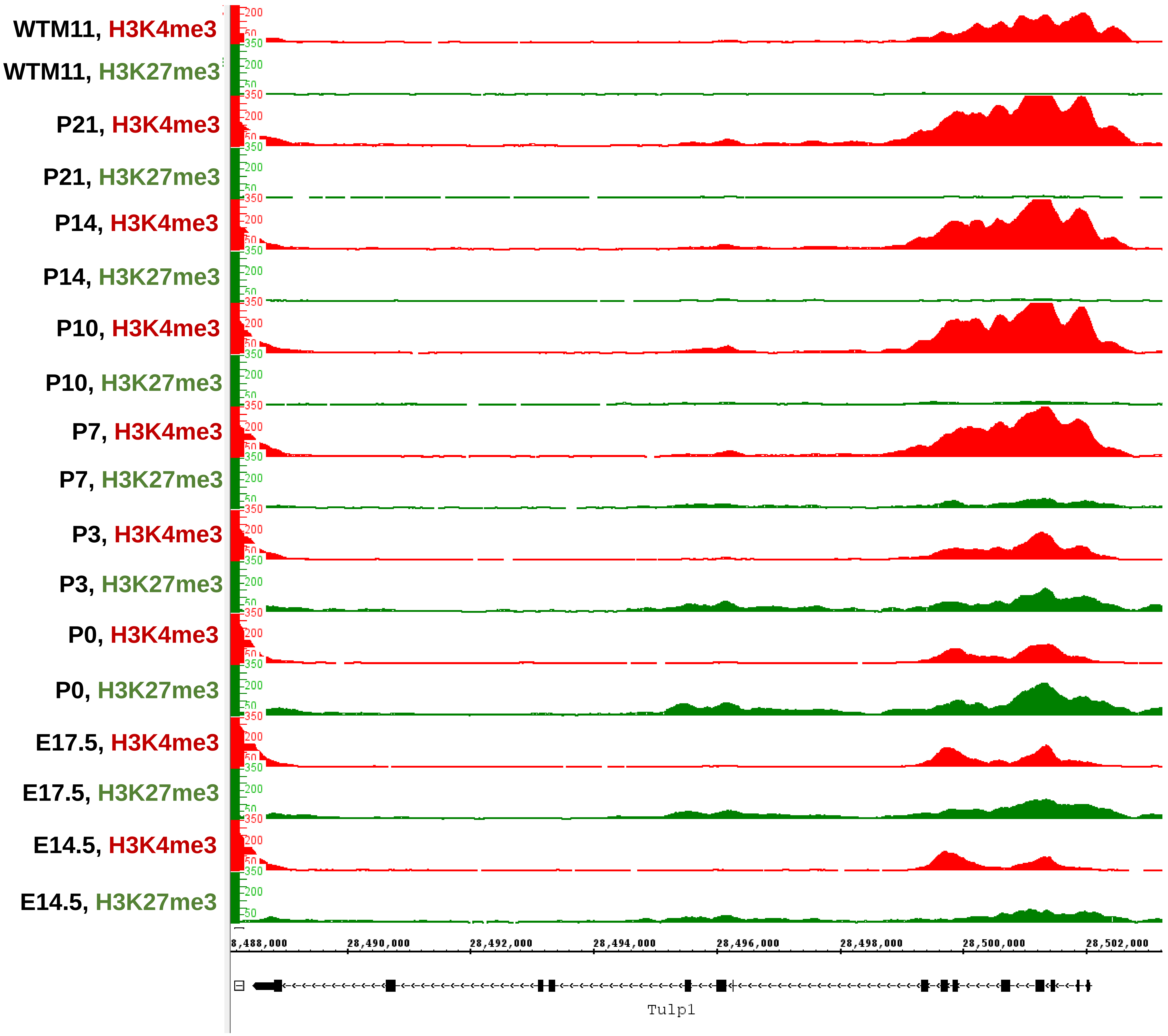

WTM11, H3K4me3
WTM11, H3K27me3
P21, H3K4me3
P21, H3K27me3
P14, H3K4me3
P14, H3K27me3
P10, H3K4me3
P10, H3K27me3
P7, H3K4me3
P7, H3K27me3
P3, H3K4me3
P3, H3K27me3
P0, H3K4me3
P0, H3K27me3
E17.5, H3K4me3
E17.5, H3K27me3
E14.5, H3K4me3
E14.5, H3K27me3
350
200
50
8,488,000
28,490,000
28,492,000
28,494,000
28,496,000
28,498,000
28,500,000
28,502,000
Tulp1

Gpr88

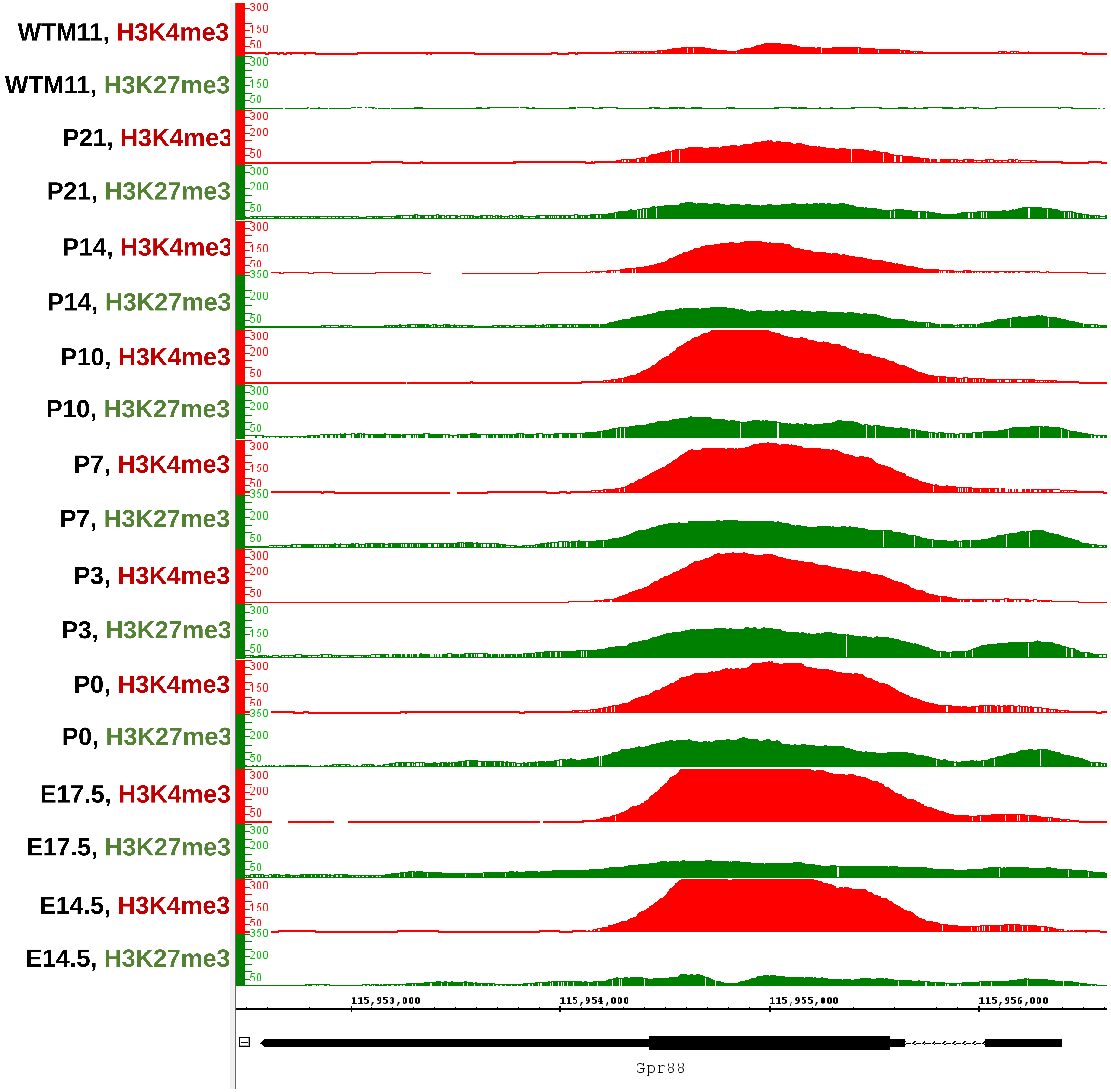
WTM11, H3K4me3
WTM11, H3K27me3
P21, H3K4me3
P21, H3K27me3
P14, H3K4me3
P14, H3K27me3
P10, H3K4me3
P10, H3K27me3
P7, H3K4me3
P7, H3K27me3
P3, H3K4me3
P3, H3K27me3
P0, H3K4me3
P0, H3K27me3
E17.5, H3K4me3
E17.5, H3K27me3
E14.5, H3K4me3
E14.5, H3K27me3
115,953,000
115,954,000
115,955,000
115,956,000
Gpr88

Prph

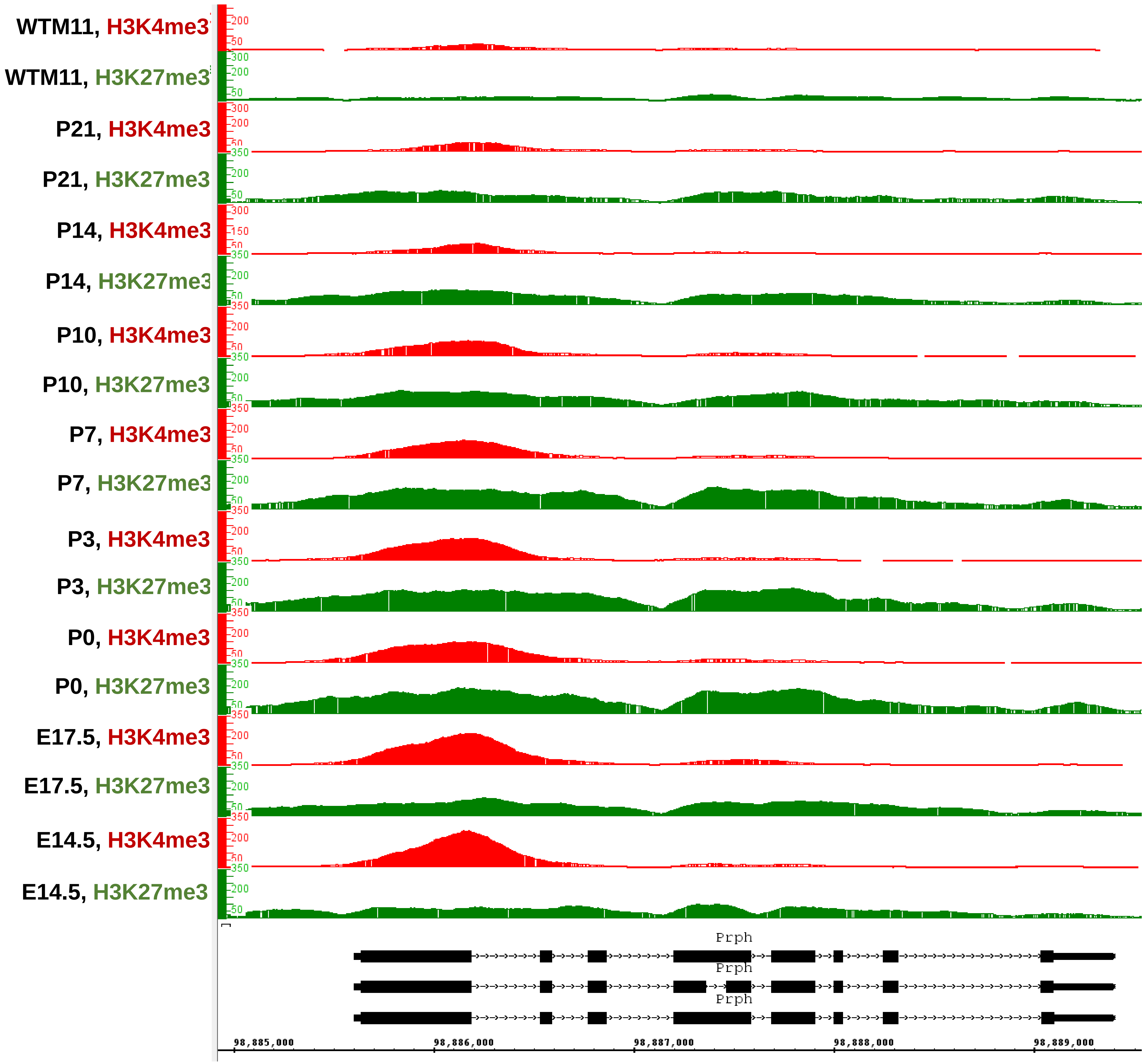
WTM11, H3K4me3
WTM11, H3K27me3
P21, H3K4me3
P21, H3K27me3
P14, H3K4me3
P14, H3K27me3
P10, H3K4me3
P10, H3K27me3
P7, H3K4me3
P7, H3K27me3
P3, H3K4me3
P3, H3K27me3
P0, H3K4me3
P0, H3K27me3
E17.5, H3K4me3
E17.5, H3K27me3
E14.5, H3K4me3
E14.5, H3K27me3
Prph
Prph
Prph
98,885,000
98,886,000
98,887,000
98,888,000
98,889,000

Ush1g

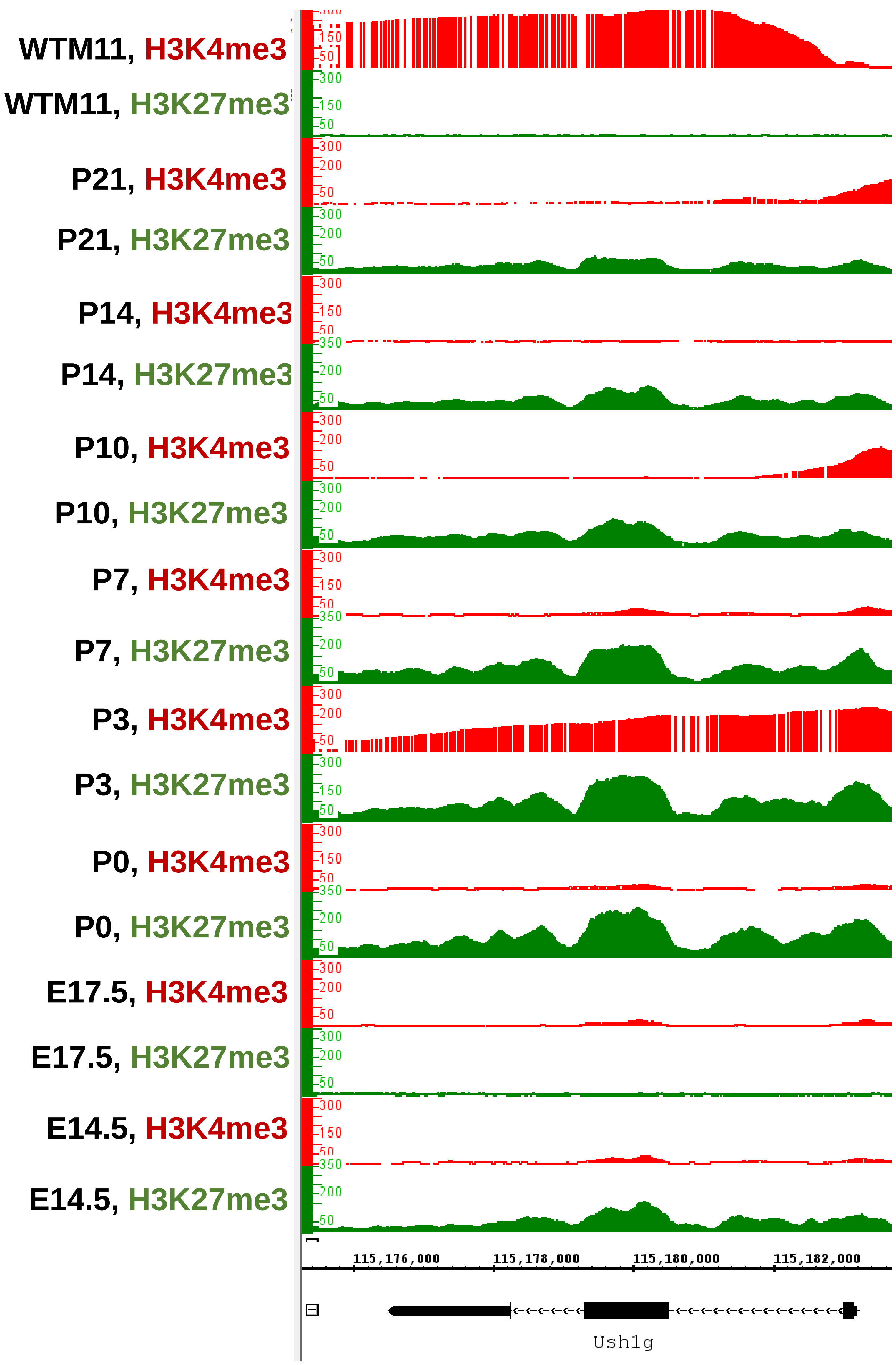
WTM11, H3K4me3
WTM11, H3K27me3
P21, H3K4me3
P21, H3K27me3
P14, H3K4me3
P14, H3K27me3
P10, H3K4me3
P10, H3K27me3
P7, H3K4me3
P7, H3K27me3
P3, H3K4me3
P3, H3K27me3
P0, H3K4me3
P0, H3K27me3
E17.5, H3K4me3
E17.5, H3K27me3
E14.5, H3K4me3
E14.5, H3K27me3
115,176,000
115,178,000
115,180,000
115,182,000
Ush1g
