# Supplementary material for: The Potential Role of Epigenetic Mechanisms in the Development of Retinitis Pigmentosa and Related Photoreceptor Dystrophies
Source: Front Genet. 2022 Mar 11;13:827274. doi: 10.3389/fgene.2022.827274 (PMC8961674; doi:10.3389/fgene.2022.827274)
Supplement: Supplementary file 7 [file DataSheet1.PDF]

Methods

Data Availability

The datasets analyzed during the current study are available in the NCBI Gene Expression Omnibus (GEO) database, accession numbers: GSE87064 (GSE86981- ChIP-Seq_Hs; GSE87037- ChIP-Seq_Mm; GSE87061- WGBS_Hs; GSE87062-WGBS_Mm; GSE87042-RNA-seq_Hs; GSE87043- RNA-Seq_Mm), GSE126474, GSE101986, GSE72550, GSE84589, and GSE80134.

Modeling of chromatin states

To integrate ChIP-seq data and identify the major combinatorial and spatial patterns of marks (chromatin states), we used ChromHMM software (http://compbio.mit.edu/ChromHMM/) according to the manual(Ernst and Kellis 2012). Briefly, downloaded from NCBI-GEO database ChIP-seq data as BAM files were binarized and then used to generate a chromatin state model according to ChromHMM algorithm. To select the chromatin state number, we modelled all human H3K4me1, H3K4me2, H3K4me3, H3K36me3, H3K27Ac, H3K27me3, H3K9-14Ac, H3K9me3, BRD4, CTCF, and RNA PolII ChIP-seq data together from 9 to 14 states. In the same way, we analyzed mouse ChIP-seq data to choose the chromatin state number. Using this computational approach, we picked 11 chromHMM chromatin states for analysis for both species. Annotation of the identified ChromHMM segments was carried out with the R Bioconductor package “Annotatr”(Cavalcante and Sartor 2017). E1-E3, E5-E8 chromatin states in humans and E1, E2, E4-E8 chromatin states in mice were considered as permissive, while E9-E11 chromatin states were considered as repressive in both species.

Modeling of methylome states

The DNA methylation analysis from the WGBS data was performed using R Bioconductor packages MethylSeekR and methylKit according to the software manuals(Akalin et al. 2012, Burger et al. 2013). Annotation of identified segments and regions was carried out with the R Bioconductor package “Annotatr” (Cavalcante and Sartor 2017). The promoter was considered as hypermethylated if it was located in a segmentation class 3 or 4 genomic region (identified by methylKit) and an unmethylated region (UMR; size >500 bp, identified by MethylSeekR) or a low-methylated region (LMR; size >500 bp, identified by MethylSeekR) were not present in the promoter area. To generate the heat maps, we first collected the mean percent of cytosine methylation in promoters of studied genes using methylKit and MethylSeekR R Bioconductor packages. Since genes may have more than one promoter, we selected the promoter with the lowest mean percent of cytosine methylation, providing that the segment (methylKit) or region (UMR or LMR; MethylSeekR) is larger than 500 bp. The mean percent of cytosine methylation was assigned to each relevant gene and used to generate heat maps using Microsoft Excel 2016. To study methylation levels for individual CpGs, we collected data of the percentage of methylation of individual cytosine bases in the promoter region (1000 bp) and first exon (500 bp) of studied genes using NCBI-GEO WGBS data and the R Bioconductor package “Annotatr”.

Statistical analysis

Data were examined for differences using One-way ANOVA or the Student’s t-test. Values of $P < 0.05$ were designated as statistically significant.

References

Akalin A, Kormaksson M, Li S, Garrett-Bakelman FE, Figueroa ME, Melnick A, Mason CE. 2012. methylKit: a comprehensive R package for the analysis of genome-wide DNA methylation profiles. Genome Biol. Oct 3;13:R87. Epub 2012/10/05.

Burger L, Gaidatzis D, Schubeler D, Stadler MB. 2013. Identification of active regulatory regions from DNA methylation data. Nucleic Acids Res. Sep;41:e155. Epub 2013/07/06.

Cavalcante RG, Sartor MA. 2017. annotatr: genomic regions in context. Bioinformatics. Aug 1;33:2381-2383. Epub 2017/04/04.

Ernst J, Kellis M. 2012. ChromHMM: automating chromatin-state discovery and characterization. Nat Methods. Feb 28;9:215-216. Epub 2012/03/01.
